# A Murinized Bimagrumab Analogue Increases Skeletal Muscle Mass and Improves Cardiometabolic Remodelling in Experimental Hypertrophic Cardiomyopathy

**DOI:** 10.1002/jcsm.70420

**Published:** 2026-11-11

**Authors:** Tomas Baka, Sophia M. Mirrione, Fuzhong Qin, Adam C. Gower, Huamei He, Connor Haney, Kuan Ming Su, Simon T. Dillon, Towia A. Libermann, Kylie Heitman, Andrew P. Ryan, Joseph T. Brozinick, Francesca Seta, David R. Pimentel, Flora Sam, Ivan Luptak

**Affiliations:** ^1^ Myocardial Biology Unit Boston University Chobanian & Avedisian School of Medicine Boston Massachusetts USA; ^2^ Section of Cardiovascular Medicine, Department of Medicine Boston Medical Center Boston Massachusetts USA; ^3^ Clinical and Translational Science Institute Boston University Chobanian & Avedisian School of Medicine Boston Massachusetts USA; ^4^ Vascular Biology Section, Department of Medicine Boston University Chobanian & Avedisian School of Medicine Boston Massachusetts USA; ^5^ Genomics, Proteomics, Bioinformatics and Systems Biology Center Beth Israel Deaconess Medical Center Boston Massachusetts USA; ^6^ Division of Interdisciplinary Medicine and Biotechnology, Department of Medicine Beth Israel Deaconess Medical Center Boston Massachusetts USA; ^7^ Harvard Medical School Boston Massachusetts USA; ^8^ Cardiometabolic Research (CMR) Eli Lilly and Company Indianapolis Indiana USA

**Keywords:** bimagrumab, hypertrophic cardiomyopathy, insulin sensitivity, myostatin, skeletal muscle

## Abstract

**Background:**

Hypertrophic cardiomyopathy (HCM) involves left ventricular hypertrophy and metabolic dysfunction. Given the role of skeletal muscle in systemic metabolism, we investigated whether activin type II receptor inhibition with CDD866, a murinized bimagrumab analogue that induces skeletal muscle hypertrophy, improves physical performance and cardiometabolic remodelling in established HCM.

**Methods:**

Twenty‐four‐week‐old male mice harbouring the cardiac myosin R403Q mutation received CDD866 or isotype antibody (20 mg/kg subcutaneously every 3 days) for 4 weeks; wild‐type (WT) littermates received isotype antibody. Assessments included body composition, muscle imaging and histology, grip strength, treadmill exercise, glucose tolerance, echocardiography, cardiac histopathology, myocardial substrate oxidation by ^13^C nuclear magnetic resonance in isolated beating hearts and cardiac transcriptomics and proteomics. Values are mean ± SEM.

**Results:**

Compared with R403Q placebo, CDD866 increased lean mass (22.5 ± 0.6 vs. 18.7 ± 0.5 g; *p* < 0.001), grip strength (135.3 ± 3.4 vs. 99.9 ± 4.3 cN; *p* < 0.0001) and treadmill distance (99.8 ± 1.7 vs. 77.2 ± 3.2 m; *p* < 0.0001). Hindlimb muscle volume increased by 24 ± 8% (*p* < 0.05), and individual muscle weights increased by 44%–87% (all *p* < 0.0001). CDD866 reduced total left ventricular wall thickness (1.80 ± 0.03 vs. 1.98 ± 0.03 mm; *p* < 0.001) and improved E/A ratio (1.31 ± 0.04 vs. 0.99 ± 0.04; *p* < 0.001) and early diastolic mitral annular velocity (18.6 ± 0.7 vs. 14.0 ± 0.7 mm/s; *p* < 0.01). Cardiomyocyte cross‐sectional area and myocardial fibrosis decreased by 32 ± 6% (*p* < 0.0001) and 69 ± 31% (*p* < 0.01), respectively. Glucose tolerance was impaired in R403Q mice versus WT (*p* < 0.01), with a nonsignificant reduction in AUC following CDD866 (*p* = 0.223). CDD866 increased the fractional contribution of fatty acids to myocardial oxidative metabolism (0.66 ± 0.02 vs. 0.38 ± 0.05; *p* < 0.01) and reduced ^13^C‐lactate output (15.3 ± 2.5 vs. 40.2 ± 3.8 nmol/min; *p* < 0.001). Consistent with these measurements, cardiac RNA‐seq GSEA showed HCM‐associated suppression and CDD866‐associated enrichment of fatty acid oxidation, pyruvate metabolism, the tricarboxylic acid cycle, oxidative phosphorylation, ATP synthesis and branched‐chain amino acid catabolism (all FDR *q* < 0.05). Integrated transcriptomic and proteomic upstream regulator predictions provided an exploratory regulatory context for these metabolic findings.

**Conclusions:**

CDD866 increased skeletal muscle mass and performance alongside improvements in cardiac remodelling, diastolic function and myocardial metabolism in established experimental HCM. Activin receptor inhibition offers a promising therapeutic avenue for HCM.

## 1 Introduction

Hypertrophic cardiomyopathy (HCM) is the most common inherited cardiac disease (prevalence of ~1:500 to 1:200) and is caused by pathogenic variants in sarcomeric proteins. The disease is characterized by left ventricular (LV) hypertrophy, hypercontractility and diastolic dysfunction and is associated with major complications including arrhythmias, sudden cardiac death and progression to heart failure [1, 2]. Although HCM is classically considered primarily a myocardial disorder, increasing evidence suggests that systemic metabolic abnormalities may exacerbate disease severity. In particular, impaired insulin sensitivity and dysregulated branched‐chain amino acid (BCAA) metabolism have been reported in HCM patients without overt diabetes or hypertension and correlate with indices of disease severity, including LV hypertrophy and functional impairment [3, 4, 5, 6].

Bimagrumab (BYM338) is a monoclonal antibody that targets activin type II receptors (ActRIIA and ActRIIB), thereby inhibiting myostatin/activin signalling and inducing robust skeletal muscle hypertrophy. In preclinical and clinical studies, bimagrumab improves body composition and skeletal muscle mass in multiple contexts, including muscle wasting and metabolic disease, and has been reported to improve insulin sensitivity in insulin‐resistant individuals [7, 8, 9, 10, 11, 12, 13, 14, 15, 16, 17, 18]. Given that skeletal muscle mediates the majority of postprandial glucose disposal and represents a major determinant of basal metabolic rate [19, 20, 21], we hypothesized that pharmacologically induced skeletal muscle hypertrophy would improve the systemic metabolic phenotype and ameliorate cardiac remodelling and metabolic dysfunction in HCM. To test this, we examined the effects of a murinized bimagrumab analogue (CDD866) in a murine model of established HCM due to the cardiac myosin R403Q mutation.

## 2 Methods

Twenty‐four‐week‐old male mice harbouring cardiac myosin R403Q mutation (R403Q‐mice) and their wild‐type (WT) littermates were treated with CDD866 (murinized bimagrumab analogue) or isotype antibody (20 mg/kg, every 3 days, subcutaneously) for 4 weeks (Figure S1). R403Q mice are an established mouse model of HCM mimicking insidious development of the HCM phenotype in humans from early age to adulthood [22, 23, 24]. Indeed, at 24 weeks of age, R403Q mice had phenotypic hallmarks of developed HCM, including LV hypertrophy, hypercontractility and diastolic dysfunction (Figure S2). Body composition measurement using EchoMRI (quantitative nuclear magnetic resonance), micro‐computed tomography (CT) imaging of lower hindlimb, skeletal muscle weighing, western blot analysis and histopathology were performed to determine skeletal muscle hypertrophy. Muscle strength and exercise tolerance tests were performed to assess skeletal muscle functional capacity. Comprehensive Laboratory Animal Monitoring System metabolic cages (CLAMS), oral glucose tolerance test (oGTT), fasting glucose and plasma insulin levels and the homeostasis model assessment of insulin resistance index (HOMA‐IR) were used to assess whole body metabolism, including insulin sensitivity. Echocardiography, cardiac histopathology, ^13^C substrate oxidation by ^13^C nuclear magnetic resonance (NMR) in isolated beating hearts, cardiac transcriptomics and proteomics were performed to assess cardiac phenotype, function and metabolism (study protocol depicted in Figure S1). Detailed methods, dataset deposition information and supplementary figures and tables are available in the Supporting Information.

## 3 Results

Consistent with prior reports [7, 8, 25], myostatin/activin signalling inhibition by CDD866 induced robust skeletal muscle hypertrophy in R403Q mice. EchoMRI analysis demonstrated a 20 ± 4% (*p* < 0.001) increase in lean mass, accompanied by a 19 ± 4% (*p* < 0.001) increase in total body water with no change in fat mass, resulting in a 12 ± 5% (*p* < 0.05) increase in body weight in CDD866‐treated R403Q mice (Figures 1A and S3). Skeletal muscle hypertrophy was corroborated by in vivo micro‐CT imaging, which showed a 24 ± 8% (*p* < 0.05) increase in lower hindlimb muscle volume (Figures 1B and S4 and 3D micro‐CT renderings in Videos [Link], [Link], [Link]). Consistently, ex vivo weights of pectoralis, triceps, quadriceps and gastrocnemius muscles were increased by 66 ± 6%, 44 ± 6%, 53 ± 5% and 87 ± 7% (all *p* < 0.0001), respectively (Figure 1B). Histological analysis of quadriceps revealed a shift towards larger fibres, with increased frequency in the 4500 μm^2^ (by 117 ± 25%, *p* < 0.01) and 5000 μm^2^ (by 186 ± 37%, *p* < 0.05) cross‐sectional area bins (Figure 1C). Functionally, CDD866 increased forelimb grip strength by 35 ± 5% (*p* < 0.0001) and treadmill exercise tolerance by 29 ± 4% (*p* < 0.0001) (Figure 1D), whereas resistance endurance was unchanged, with no difference in wire or screen hang performance (Figures S5 and S6). Given the dissociation between improved treadmill performance and unchanged resistance endurance, the increase in exercise tolerance is unlikely to be explained solely by skeletal muscle endurance suggesting a contribution from improved cardiovascular performance, as supported by the cardiac findings described below.

**FIGURE 1:**
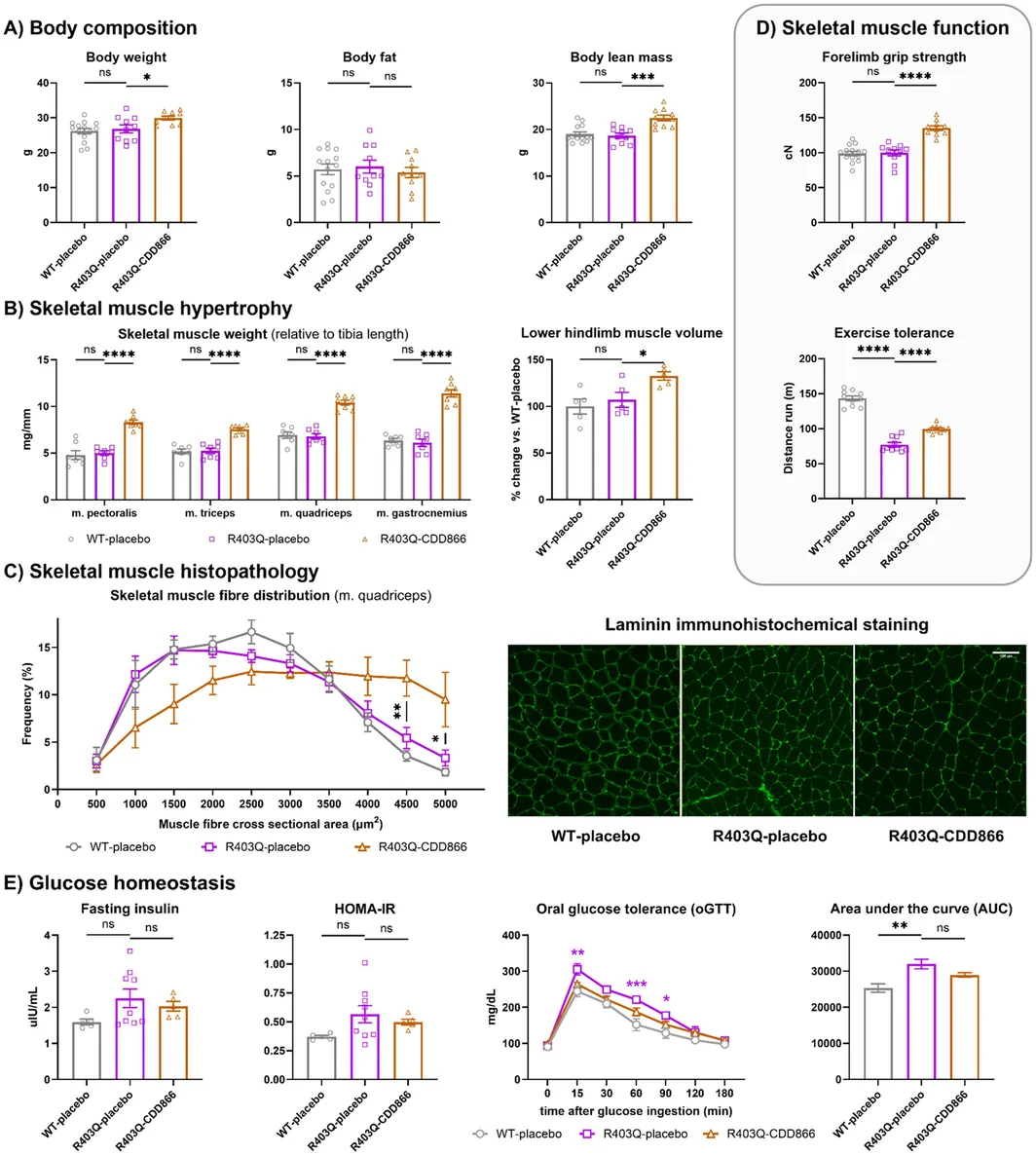
Inhibition of myostatin/activin signalling with CDD866 increases skeletal muscle mass and improves glucose tolerance in R403Q mice with hypertrophic cardiomyopathy. A 4‐week CDD866 treatment induced skeletal muscle hypertrophy in R403Q mice, as evidenced by (A) EchoMRI showing increased body weight and lean mass with no change in fat mass, (B) micro‐CT showing increased lower hindlimb muscle volume and increased ex vivo weights of isolated skeletal muscles (pectoralis, triceps, quadriceps and gastrocnemius) and (C) quadriceps histology showing a shift towards larger myofibres with increased cross‐sectional area. (D) CDD866 increased forelimb grip strength and treadmill exercise tolerance, whereas resistance endurance was unchanged (Figures S5 and S6), suggesting that improved exercise tolerance is not explained solely by skeletal muscle endurance. (E) R403Q mice showed impaired glucose tolerance (oGTT), accompanied by nonsignificant increases in fasting insulin and HOMA‐IR, impaired oGTT was attenuated by CDD866. Data are mean ± SEM; *n* = 3–14. One‐way ANOVA with Bonferroni multiple comparisons tests (adjusted *p* value threshold of < 0.05) (oGTT: mixed‐effects model with Bonferroni multiple comparisons tests). *p < 0.05, **p < 0.01, ***p < 0.001, ****p < 0.0001. Representative laminin immunofluorescence images are shown; scale bar, 100 μm.

Placebo‐treated R403Q mice with established HCM showed evidence of impaired glucose handling with mild‐to‐moderate impairment of glucose tolerance, in line with prior clinical observations [3]. Similarly, fasting plasma insulin, glucose and calculated HOMA‐IR were higher in R403Q mice versus WT littermates (by 41 ± 16%, 7 ± 7% and 53 ± 18%, respectively), although these differences did not reach statistical significance (Figures 1E and S7). However, impaired glucose tolerance in R403Q mice became more apparent during oral glucose tolerance testing (oGTT), with higher glucose levels at 15, 30, 60 and 90 min after glucose ingestion (by 25 ± 8%, *p* < 0.01; 19 ± 6%, *p* = ns; 46 ± 12%, *p* < 0.001; and 38 ± 12%, *p* < 0.05, respectively), resulting in a 26 ± 6% (*p* < 0.01) increase in oGTT area under the curve (AUC) (Figure 1E). Notably, CDD866 attenuated the impaired glucose tolerance in R403Q mice.

To explore whether skeletal muscle metabolic pathways were altered by CDD866, we investigated the regulators of BCAA catabolism in quadriceps muscle. CDD866 treatment was associated with nonsignificant trends towards increased branched‐chain ketoacid dehydrogenase kinase (BCKDK) expression (by 124 ± 37%, *p* = ns) and increased inhibitory phosphorylation of the branched‐chain ketoacid dehydrogenase E1 subunit alpha (BCKDHA; the ratio phosphorylated/total BCKDHA increased by 33 ± 51%, *p* = ns) (Figure S8). Because these changes did not reach statistical significance and BCAA concentrations or flux were not directly measured, these findings should be considered exploratory and do not establish altered skeletal muscle BCAA catabolism. Whole‐body metabolic phenotyping in CLAMS cages revealed no major differences in respiratory exchange ratio (RER) among groups, with preserved circadian oscillation (Figure S9). Importantly, ambulatory activity was unchanged by CDD866 (Figure S10), supporting that increased muscle mass occurred without evidence of increased physical activity.

As expected, the developed HCM phenotype present in 24‐week‐old R403Q mice prior to treatment (Figure S2) remained evident after 4 weeks of placebo. Echocardiography confirmed marked LV hypertrophy, with increased total wall thickness by 21 ± 2% (*p* < 0.0001), accompanied by concentric remodelling, as LV end‐systolic and end‐diastolic diameters were reduced by 32 ± 8% (*p* < 0.0001) and 16 ± 5% (*p* < 0.001), respectively (Figures 2A and S11). R403Q mice also exhibited hypercontractility, with fractional shortening increased by 49 ± 7% (*p* < 0.0001), and diastolic dysfunction, with both E/A ratio and Em reduced by 37 ± 6% and 39 ± 7% (both *p* < 0.0001), respectively (Figure 2A). Consistent with these functional abnormalities, histopathology demonstrated increased cardiomyocyte cross‐sectional area (by 93 ± 5%, *p* < 0.0001) with markedly increased fibrosis (by 509 ± 30%, *p* < 0.001) (Figure 2B). Supporting our hypothesis, CDD866 improved key hallmarks of established HCM in R403Q mice. CDD866 reduced LV remodelling, decreasing total wall thickness by 9 ± 2% (*p* < 0.001), cardiomyocyte cross‐sectional area by 32 ± 6% (*p* < 0.0001) and fibrosis by 69 ± 31% (*p* < 0.01) (Figure 2B). LV end‐systolic and end‐diastolic diameters increased by 15 ± 7% (*p* = ns) and 14 ± 4% (*p* < 0.01), respectively (Figure S11). Importantly, CDD866 improved diastolic function, with E/A ratio and Em increased by 33 ± 6% (*p* < 0.001) and 33 ± 6% (*p* < 0.01), respectively (Figure 2A).

**FIGURE 2:**
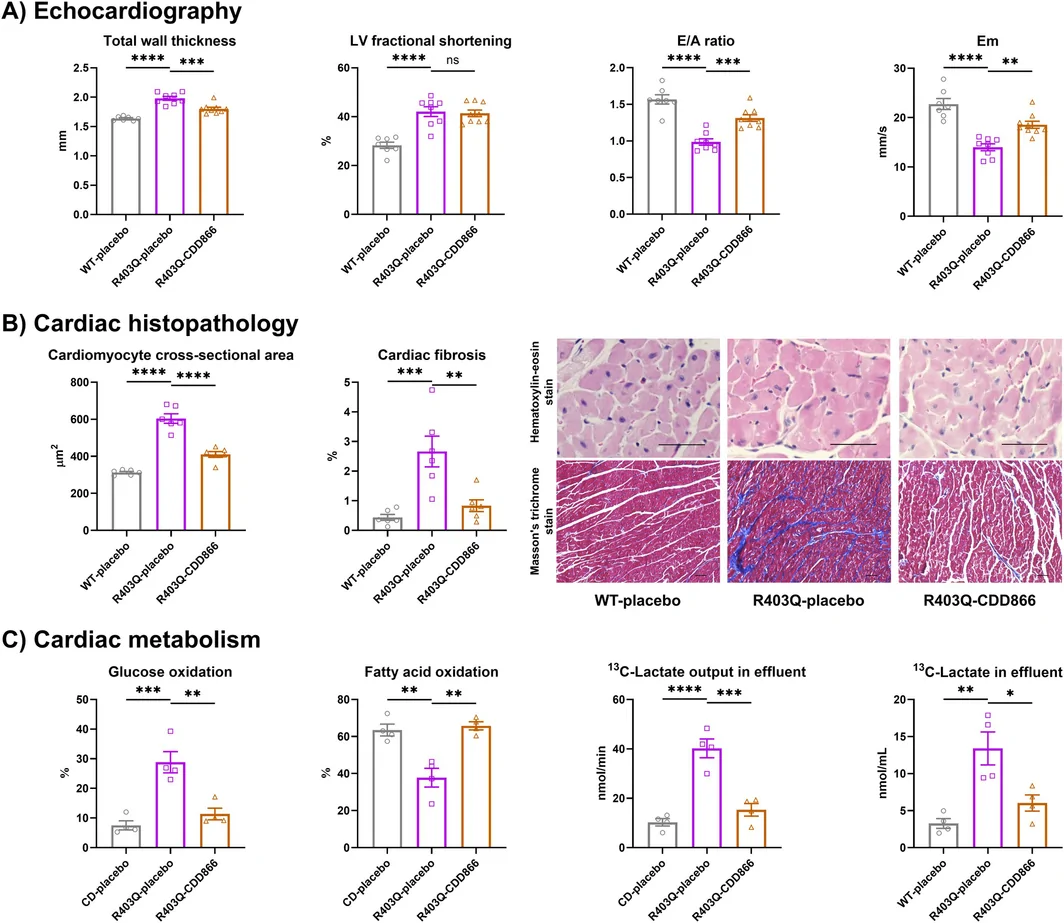
Inhibition of myostatin/activin signalling with CDD866 improves cardiac phenotype and metabolism in R403Q mice with hypertrophic cardiomyopathy. Twenty‐four‐week‐old R403Q mice exhibited established HCM prior to treatment (Figure S2), and these hallmarks persisted after 4 weeks of placebo. (A) Echocardiography demonstrating LV hypertrophy, hypercontractility (fractional shortening) and diastolic dysfunction (E/A ratio and Em) (see Figure S11 for LV geometry indices). (B) Cardiac histopathology showing increased cardiomyocyte cross‐sectional area and fibrosis; both were improved by CDD866. (C) Ex vivo ^13^C substrate oxidation demonstrating a pathological shift in R403Q hearts from fatty acid oxidation towards glucose utilization with increased lactate production; CDD866 normalized substrate utilization and lactate output. Data are mean ± SEM; *n* = 4–9. One‐way ANOVA with Bonferroni multiple comparisons tests (adjusted *p* value threshold of < 0.05). *p < 0.05, **p < 0.01, ***p < 0.001, ****p < 0.0001. Representative images: haematoxylin–eosin (myocyte cross‐sectional area) and Masson's trichrome (fibrosis); scale bar, 50 μm.

In line with our previous findings [24], R403Q hearts exhibited a metabolic shift away from fatty acid oxidation (FAO) towards increased glucose utilization, accompanied by markedly increased lactate production, consistent with impaired coupling between glycolysis and glucose oxidation. In placebo‐treated R403Q hearts, FAO was reduced by 41 ± 14% (*p* < 0.01), whereas glucose oxidation increased by 283 ± 24% (*p* < 0.001); notably, lactate output and lactate concentration in the effluent were increased by 291 ± 17% (*p* < 0.0001) and 310 ± 26% (*p* < 0.01), respectively (Figure 2C). CDD866 normalized substrate oxidation patterns and suppressed excess lactate production in R403Q hearts, supporting improved metabolic flexibility and restored glycolysis–glucose oxidation coupling (Figure 2C).

Principal component analysis (PCA) of cardiac transcriptomes using the first two principal components demonstrated distinct clustering of the study groups. WT‐placebo and R403Q‐placebo separated clearly, whereas R403Q‐CDD866 localized between them, consistent with partial shift of the R403Q transcriptional signature towards WT (Figure 3). Gene set enrichment analysis (GSEA) in placebo‐treated R403Q hearts revealed upregulation of hypertrophic and remodelling pathways, including collagen formation, extracellular matrix organization and epithelial‐to‐mesenchymal transition, together with broad downregulation of metabolic and mitochondrial programmes. These included FAO, pyruvate metabolism, tricarboxylic acid (TCA) cycle, oxidative phosphorylation, ATP biosynthesis and pathways related to mitochondrial structure and dynamics (membrane/matrix/cristae components, fusion/fission, biogenesis and ion transport). In addition, BCAA catabolism and insulin‐related pathways (cellular response to insulin and insulin signalling) were suppressed, and pathways related to cardiac development and regulation of cardiac action potential were altered. Notably, CDD866 mitigated many of these coordinated transcriptional changes in R403Q hearts (Figure 3 and Tables S1 and S2), with several oppositely regulated gene sets ranking among the 20 most upregulated or downregulated pathways across comparisons (Tables S3 and S4).

**FIGURE 3:**
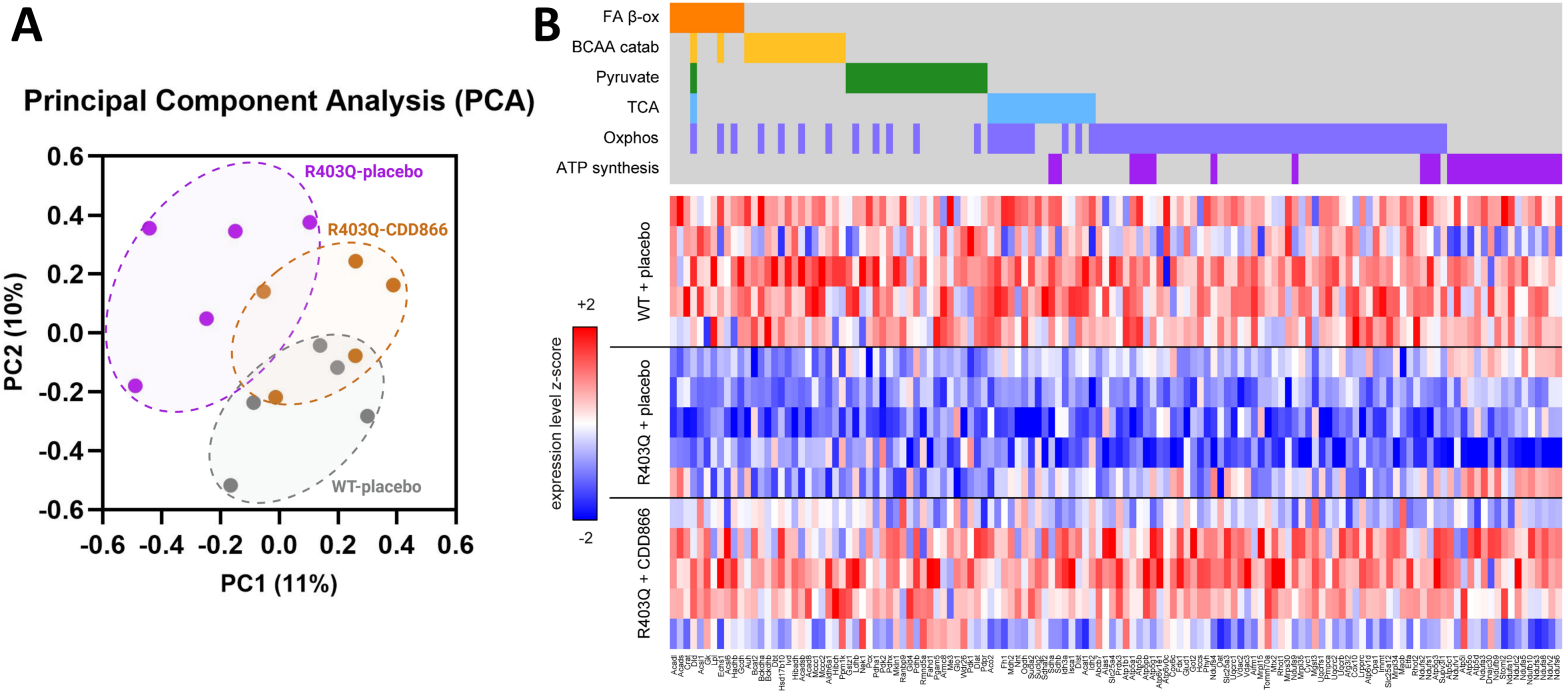
Inhibition of myostatin/activin signalling with CDD866 restores cardiac transcriptomics deregulations in R403Q mice with hypertrophic cardiomyopathy. PCA (PC1 vs. PC2) showing separation of WT‐placebo and R403Q‐placebo, with R403Q‐CDD866 positioned between them, consistent with partial normalization of the R403Q transcriptional signature. Heatmap shows the union of leading‐edge genes from six metabolic gene sets coordinately downregulated in R403Q‐placebo vs. WT‐placebo and restored by CDD866 (FDR *q* < 0.05 for both comparisons): FA β‐ox (WP143), BCAA catabolism (R‐HSA‐70895), pyruvate metabolism (R‐HSA‐70268), TCA cycle (R‐HSA‐71403), oxidative phosphorylation (Hallmark) and ATP synthesis (GO:0015986).

Cardiac proteomic profiling identified molecular changes associated with the R403Q phenotype and CDD866 treatment. At a nominal *p* < 0.05 threshold, 412 proteins differed in abundance between R403Q‐placebo and WT‐placebo hearts. Of these, 119 also differed between R403Q‐CDD866 and R403Q‐placebo hearts, including 62 that changed in opposite directions across the two comparisons (Figure S13A). These oppositely regulated proteins included factors involved in cellular homeostasis and stress responses, cytoskeletal organization, membrane dynamics, immune signalling, mitochondrial function, Ca^2+^ handling and intermediary metabolism, including glucose, fatty acid and BCAA metabolism (Tables S5 and S6). Volcano plots are shown in Figure S12, and the 20 most upregulated or downregulated proteins in each comparison are listed in Tables S7 and S8. Ingenuity Pathway Analysis identified disease/function annotations related to cell survival, viability, apoptosis, inflammation and pathological growth at nominal overlap *p* < 0.05. Several associated activation *z*‐scores showed opposite directions in the disease and treatment comparisons, suggesting counter‐regulation of disease‐associated molecular programmes by CDD866 (Figure S13B). Glucose‐, insulin‐ and AKT‐related upstream regulator predictions likewise showed parallel negative disease‐associated and positive treatmen*t*‐associated *z*‐scores. Although these directional scores were modest, their agreement suggests treatmen*t*‐associated modulation of metabolic signalling. Collectively, these proteomic patterns support a broader picture of cardiometabolic remodelling involving intermediary metabolism and cellular stress responses. No individual proteins remained significant after Benjamini–Hochberg correction for multiple testing, and ingenuity pathway analysis (IPA) overlap *p* values were unadjusted; accordingly, these findings remain exploratory and hypothesis‐generating.

Integration of cardiac transcriptomic and proteomic IPA analyses identified 88 upstream regulators meeting nominal overlap *p* < 0.05 in both disease and treatment comparisons in both omics layers (Table S9). Eight selected regulators with concordant treatmen*t*‐associated directions are shown in Figure 4. PPARG (peroxisome proliferator‐activated receptor gamma), MYC, EGFR (epidermal growth factor receptor), EP300 (histone acetyltransferase p300) and the insulin family showed negative disease‐associated and positive treatmen*t*‐associated activation *z*‐scores across both platforms. HNF4A (hepatocyte nuclear factor 4 alpha) showed the opposite sign pattern, with positive disease‐associated and negative treatmen*t*‐associated scores in both platforms. PPARGC1A (peroxisome proliferator–activated receptor gamma coactivator 1‐alpha) and NFE2L2 (nuclear factor erythroid 2‐related factor 2) also showed concordant positive treatmen*t*‐associated scores, but their disease‐associated directions differed between platforms. The PPARGC1A predictions provide a plausible regulatory connection to the improvement in mitochondrial oxidative metabolism programmes identified by RNA‐seq GSEA, although its treatmen*t*‐associated scores were modest in both RNA‐seq and proteomics (*z* = 0.60 and 1.32, respectively). Shared positive treatmen*t*‐associated predictions for NFE2L2 additionally suggested involvement of cellular stress response programmes. These findings indicate partial convergence of transcriptomic and proteomic regulatory signatures associated with CDD866 treatment, without establishing a mechanism underlying the observed improvements in glucose tolerance or myocardial substrate utilization. The upstream regulator analyses remain exploratory because many directional predictions were modest, MYC, EGFR and EP300 carried IPA directional bias flags in the RNA treatment analysis, and no individual SomaScan proteins survived Benjamini–Hochberg false discovery rate (FDR) correction.

**FIGURE 4:**
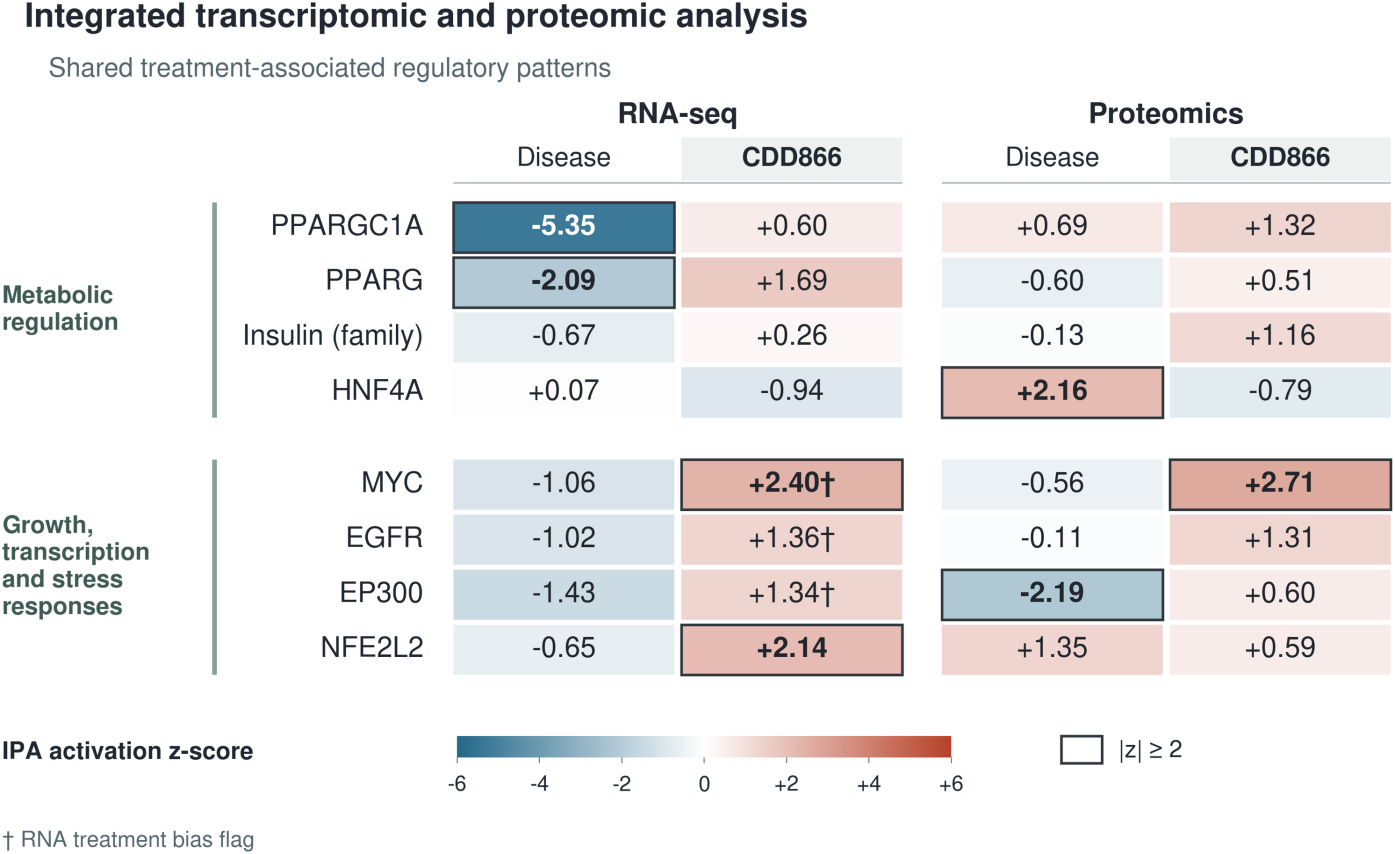
Integrated transcriptomic and proteomic analysis. Heatmap of ingenuity pathway analysis (IPA) activation *z*‐scores for eight selected upstream regulators with concordant treatmen*t*‐associated directions in cardiac RNA‐seq and SomaScan proteomics. Regulators were selected for biological relevance from the 88 entries meeting nominal overlap *p* < 0.05 in both disease and treatment comparisons in each omics layer (Table S9). Regulators are grouped by broad biological role. Disease denotes R403Q‐placebo versus WT‐placebo; CDD866 denotes R403Q‐CDD866 versus R403Q‐placebo. Blue and red indicate negative and positive *z*‐scores, respectively, on a shared linear scale from −6 to +6. Outlined cells with bold numbers indicate |*z*| ≥ 2; smaller absolute scores represent weaker directional predictions. A dagger identifies an IPA directional bias flag in the RNA treatment export. Overlap *p* values were not adjusted for multiple testing. The panel presents selected examples of shared treatment direction, not an exhaustive regulator list or uniform reversal of disease predictions. Regulator activity is inferred from downstream target patterns and was not directly measured. The proteomic component is exploratory because no individual proteins survived Benjamini–Hochberg false discovery rate (FDR) correction.

## 4 Discussion

In this study, we demonstrate that pharmacologic inhibition of myostatin/activin signalling with CDD866 induces robust skeletal muscle hypertrophy and improves skeletal muscle dysfunction in an established murine model of sarcomeric HCM. These systemic changes were accompanied by improved whole‐body metabolic phenotype and were associated with attenuation of LV hypertrophy and fibrosis, improvement of LV diastolic function and normalization of myocardial substrate utilization. Together, these findings support the hypothesis that skeletal muscle may represent a modifiable therapeutic target in HCM and provide proof‐of‐concept evidence that a muscle‐directed pharmacologic intervention can be linked to favourable cardiac remodelling and metabolic reprogramming in HCM.

These findings are notable in the context of emerging clinical and experimental evidence that HCM is associated with a systemic metabolic derangement. Prior work showed that HCM patients without apparent diabetes mellitus or hypertension exhibit insulin resistance as measured by HOMA‐IR, which correlated with disease severity indices including interventricular septal thickness, LV outflow tract pressure gradient and sudden death risk [3]. Elevated circulating levels of BCAAs and their metabolites have also been reported in HCM patients and correlate with LV hypertrophy and impaired cardiac function [4, 5]. Consistent with these observations, cardiac proteomic analyses of HCM myocardium demonstrated accumulation of BCAAs and reduced expression of key enzymes involved in BCAA catabolism [6]. Collectively, these clinical data support the concept that systemic metabolic abnormalities in HCM may extend beyond comorbidities and could modulate phenotype severity. Moreover, peripheral involvement may not be limited to metabolism: Subclinical skeletal muscle abnormalities have been described in HCM, as conventional electromyography and muscle biopsy identified myopathic findings in a subset of patients, suggesting that skeletal muscle involvement may accompany the cardiac phenotype [26].

Our data support the hypothesis that increasing skeletal muscle mass can improve systemic metabolic phenotype in HCM. Skeletal muscle is a dominant site for glucose disposal and contributes substantially to whole‐body energy expenditure [19, 20, 21]. Accordingly, it has been proposed that skeletal muscle hypertrophy can act as a ‘metabolic sink’ for nutrient surplus, including glucose and BCAAs, thereby improving insulin sensitivity and metabolic flexibility [27, 28, 29, 30]. CDD866‐induced skeletal muscle hypertrophy was accompanied by improved glucose tolerance and favourable cardiac remodelling. Skeletal muscle–mediated metabolic crosstalk represents one potential contributor to these effects, although the present study does not establish causality. These observations are consistent with prior human data showing that bimagrumab improves body composition and insulin sensitivity in insulin‐resistant individuals [11]. At the cardiac level, the agreement between functional metabolic measurements and omics analyses supports coordinated metabolic remodelling associated with CDD866 treatment. The shift towards FAO assessed by ^13^C NMR and the reduction in lactate output were consistent with the opposing disease‐ and treatmen*t*‐associated changes in FAO, pyruvate and TCA cycle metabolism and oxidative phosphorylation gene programmes identified by GSEA. Positive treatmen*t*‐associated upstream regulator predictions for PPARG and PPARGC1A in both RNA‐seq and proteomics provided a plausible regulatory connection to these metabolic changes. Together, these findings link the measured myocardial metabolic phenotype with corresponding transcriptional programmes and candidate upstream regulatory signatures, strengthening the biological interpretation of the integrated analyses (Figures 2C, 3 and 4).

ActRII inhibition has also been reported to influence brown adipose tissue (BAT) and thermogenesis. Fournier et al. demonstrated increased BAT‐associated mitochondrial activity and nonshivering thermogenesis following ActRII blockade [31], raising the possibility that adipose tissue could contribute to the systemic metabolic effects observed here. We did not directly assess BAT mass or BAT‐specific thermogenic activity. Although CDD866 did not significantly increase whole‐body heat production or energy expenditure in CLAMS studies performed at 24°C, these measurements cannot exclude tissue‐specific changes in BAT thermogenesis. Thus, adipose tissue represents another potential contributor to the systemic response to ActRII inhibition. Of note, increased circulating or myocardial myostatin and activin levels have been reported in several cardiovascular diseases associated with pathological remodelling, including myocardial infarction, hypertensive heart disease and heart failure [32, 33, 34]. In the present R403Q model, however, cardiac proteomic analysis did not detect significant differences in myostatin, activin A or activin B abundance compared with WT hearts. The absence of statistically significant differences in ligand abundance does not exclude altered receptor activity or downstream signalling. Total and phosphorylated SMAD2/3 were not measured in cardiac tissue; therefore, we could not determine whether cardiac SMAD2/3 signalling was altered in R403Q mice or modulated by CDD866 treatment. Direct effects of ActRII blockade on cardiac cells remain plausible contributors to the observed benefits, alongside indirect metabolic and systemic effects.

Although the present study was performed in a genetic model of HCM, available experimental data suggest that the beneficial effects of myostatin/activin pathway inhibition may extend to non‐genetic forms of cardiac hypertrophy and heart failure. In our preliminary data presented in abstract form, the murinized bimagrumab analogue (CDD866) increased skeletal muscle mass and improved glucose tolerance in a mouse model of obesity‐related diabetic cardiomyopathy, changes that were associated with reduced LV hypertrophy and improved diastolic function [35]. Similar cardiac benefits were observed following skeletal muscle‐specific disruption of myostatin/activin signalling in the same model [36], supporting an important contribution of skeletal muscle hypertrophy to the cardiac benefit in metabolic cardiomyopathy. These findings are also consistent with studies from other groups. Roh et al. reported that activin receptor blockade using either clinical‐stage inhibitors or genetic approaches improved cardiac function in multiple models of heart failure associated with aging and pressure overload [37]. Collectively, these studies suggest that the benefits of activin pathway inhibition are not restricted to a single genetic HCM model and may extend to other forms of pathological cardiac hypertrophy and heart failure. However, the magnitude and mechanisms of benefit are likely to depend on the underlying disease context and will require direct evaluation in additional models.

The similarities between the present findings and our prior work with the SGLT2 inhibitor empagliflozin in the same murine HCM model are striking [24]. In that study, empagliflozin restored mitochondrial oxidative capacity, improved coupling of glycolysis to glucose oxidation, normalized elevated intracellular sodium and improved myocardial energetics; changes that were associated with reduced LV hypertrophy and fibrosis and improved diastolic function and contractile reserve [24]. Here, CDD866 similarly improved cardiac remodelling and the myocardial metabolic phenotype. Empagliflozin has well‐established insulin‐sensitizing effects [38, 39, 40], and in our prior work, it was associated with normalization of cardiac gene sets related to insulin signalling [24]. Taken together, these findings raise the possibility that improved insulin sensitivity and systemic metabolic remodelling may represent a convergent pathway through which distinct therapeutic strategies—including SGLT2 and myostatin/activin inhibition—exert disease‐modifying effects in sarcomeric HCM (Figure 5).

**FIGURE 5:**
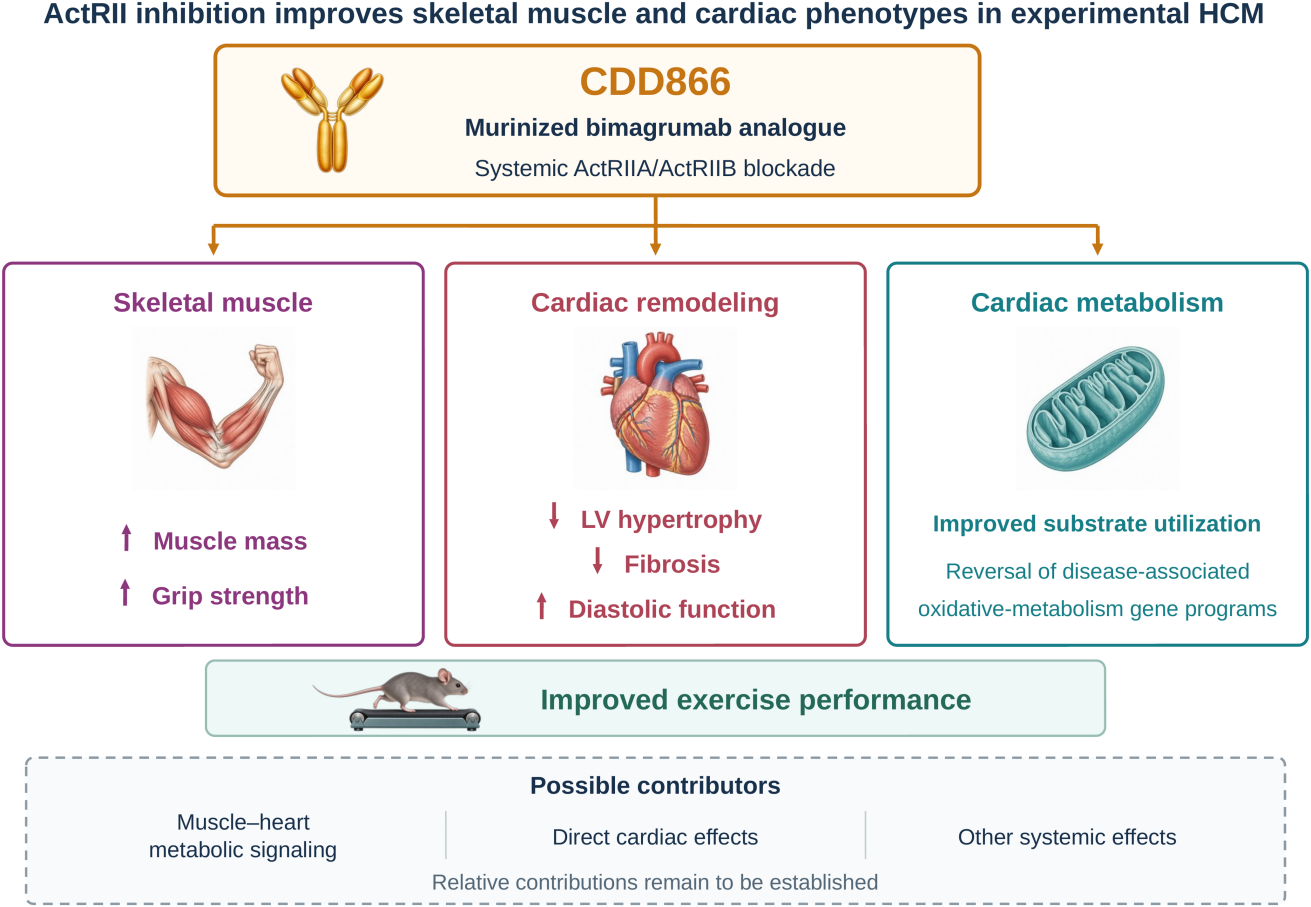
Graphical summary. Activin type II receptors (ActRII) inhibition improves skeletal muscle and cardiac phenotypes in experimental HCM. Four weeks of treatment with CDD866, a murinized bimagrumab analogue targeting ActRIIA/ActRIIB, increased skeletal muscle mass and grip strength and improved exercise performance in male R403Q mice with established hypertrophic cardiomyopathy (HCM). Treatment was accompanied by reduced LV hypertrophy and fibrosis, improved diastolic function and myocardial substrate utilization and opposing disease‐ and treatmen*t*‐associated changes in oxidative metabolic gene programmes identified by cardiac RNA‐seq GSEA. Solid arrows summarize treatmen*t*‐associated findings; they do not establish that skeletal muscle hypertrophy mediates the cardiac effects. The dashed box identifies potential contributors, including muscle–heart metabolic signalling, direct cardiac effects and other systemic effects, whose relative contributions remain unresolved. Other systemic effects may include adipose/BAT, endocrine and inflammatory mechanisms. GSEA, gene set enrichment analysis; HCM, hypertrophic cardiomyopathy; LV, left ventricular; Anatomical illustrations were generated using OpenAI's image generation tool.

From a translational perspective, these findings are particularly relevant as therapeutic options remain limited for many symptomatic patients with HCM, especially those without clear candidates for septal reduction therapy or cardiac myosin inhibitors. Lifestyle interventions including structured exercise are increasingly encouraged after individualized risk assessment, yet high‐intensity training remains restricted to carefully selected patients under expert guidance [41, 42]. Furthermore, genotype‐specific skeletal muscle abnormalities and subclinical myopathy have been reported in subsets of HCM patients and may contribute to muscle weakness and exercise limitation [26, 43, 44]. In this context, pharmacologic strategies that increase skeletal muscle mass without requiring high‐intensity training could have clinical value. Our findings, therefore, support the consideration of muscle‐directed therapies as adjunctive strategies in HCM, particularly for patients with exercise limitation or evidence of peripheral muscle dysfunction.

Bimagrumab has consistently been shown to improve body composition by increasing skeletal muscle mass and reducing fat mass in clinical studies [8, 10]. It has been investigated in muscle‐wasting conditions including sporadic inclusion body myositis [13], sarcopenia [14], casting‐induced muscle atrophy [15] and obesity and has been shown in the phase 2 BELIEVE trial to augment fat loss and attenuate lean‐mass loss when added to the glucagon‐like peptide‐1 (GLP‐1) receptor agonist semaglutide [10]. Across these studies, bimagrumab has generally been well‐tolerated. More recently, cardiac safety has been specifically evaluated. In the randomized, double‐blind, placebo‐controlled phase 2b RESILIENT trial, in inclusion body myositis, no clinically significant adverse effects were detected by electrocardiography or echocardiography [12]. In a separate study evaluating cardiac safety in healthy older adults, bimagrumab increased total lean body mass and reduced fat mass without significant changes in LV mass index or ejection fraction [45]. Although available clinical studies provide reassuring cardiac safety data, bimagrumab has not yet been evaluated in a clinical trial specifically designed to assess therapeutic cardiovascular effects or cardiovascular outcomes.

This study has limitations. The experiments were performed in a single genetic HCM model and in male mice with established disease as male R403Q mice have been shown to demonstrate a more consistent HCM phenotype [22, 46]. Additional work will be required to define the generalizability of these findings across genotypes, sexes and disease stages. The mechanistic link between skeletal muscle remodelling, systemic metabolic phenotype and cardiac remodelling also warrants further investigation, including the relative contributions of insulin signalling, BCAA metabolism and other endocrine or inflammatory pathways. Nonetheless, the coordinated skeletal muscle, systemic metabolic and cardiac responses observed with CDD866 support further investigation of interorgan metabolic mechanisms and ActRII inhibition in HCM. BCAA‐related pathways emerged in both transcriptomic and proteomic analyses; however, BCAA concentrations, branched‐chain ketoacids and BCAA flux were not directly measured. These findings therefore generate a hypothesis that BCAA metabolism may contribute to the observed cardiometabolic remodelling, but they do not establish a mechanistic role. Because CDD866 systemically inhibits ActRIIA/ActRIIB signalling, the present study cannot determine whether the cardiac benefits are mediated primarily by skeletal muscle remodelling or by direct effects on cardiac and other peripheral tissues. Activin/myostatin signalling is active in multiple cell types, including cardiomyocytes and cardiac fibroblasts, and modulation of these pathways could directly influence myocardial remodelling, fibrosis, metabolism or inflammatory signalling. Effects on adipose tissue and other endocrine or metabolic organs may also contribute. Thus, skeletal muscle hypertrophy should be viewed as one plausible component of a broader systemic response to ActRII blockade rather than as the sole established mediator of the cardiac phenotype. Furthermore, no individual SomaScan proteins remained significant after Benjamini–Hochberg correction, and IPA overlap *p* values were unadjusted; therefore, the proteomic and integrated upstream regulator analyses remain exploratory. Their agreement with GSEA and the myocardial metabolic measurements provides a biologically coherent basis for identifying candidate regulatory programmes, but does not establish causal roles for individual regulators. These candidates warrant validation in independent cohorts and targeted mechanistic studies.

In conclusion, myostatin/activin receptor inhibition with CDD866 induced robust skeletal muscle hypertrophy and improved muscle strength and exercise tolerance in a murine model of sarcomeric HCM. These systemic effects were accompanied by improved metabolic phenotype, reversal of LV hypertrophy and fibrosis and improvement in myocardial substrate utilization. These findings support further investigation of ActRII pathway inhibition as a potential adjunctive therapeutic strategy in HCM and motivate studies to define the relative contributions of skeletal muscle, direct cardiac and other systemic effects.

## Funding

This research was supported by the National Heart, Lung, and Blood Institute (NHLBI) at the National Institutes of Health (NIH) grants No. 1R56HL157367‐01A1 (to I.L.) and 1R01HL166606‐01A1 (to I.L.), a Fellow‐to‐Faculty Award from the American Heart Association (AHA) 15FTF25890062 (to I.L.), National Institutes of Health (NIH) Shared Instrumentation Grants (SIG) 1S10OD021652‐01 and 1S10OD030501‐01A1 (to F. Seta) and Clinical Translational Science Award grant UL1TR001430 (to A.C.G.).

## Ethics Statement

The protocol was approved by the Institutional Animal Care and Use Committee at Boston University Chobanian & Avedisian School of Medicine (Boston, MA, USA).

## Conflicts of Interest

I.L. was supported by Amgen Inc. (Thousand Oaks, CA, USA), Avidity Biosciences Inc. (San Diego, CA, USA), Cytokinetics Inc. (South San Francisco, CA, USA), Eli Lilly and Co. (Indianapolis, IN, USA) and Novartis Pharmaceuticals (Cambridge, MA, USA). K.H., A.P.R., J.T.B. and F. Sam are current employees of Eli Lilly and Co. and own company stock. The remaining authors have no disclosures to report.

## Supporting information


**Figure S1:** Study protocol. 24‐week‐old male mice harbouring R403Q mutation in cardiac myosin heavy chain or their wild‐type littermates were randomized by body weight into three groups: (i) wild‐type‐placebo (WT‐placebo); (ii) R403Q‐placebo; and (iii) R403Q‐CDD866. At 24 weeks of age, male mice with the cardiac myosin R403Q mutation recapitulate hallmarks of developed hypertrophic cardiomyopathy (HCM) including left ventricular (LV) hypertrophy, fibrosis and diastolic dysfunction. At this age, mice were treated with the antibody CDD866 (murinized bimagrumab) targeting the myostatin/activin receptor ActRII or isotype antibody serving as placebo (20 mg/kg, subcutaneously, every 3 days) for 4 weeks. At the end of the 4‐week treatment period, mice underwent echocardiography and heart isolation to determine 13C‐substrate oxidation. Hearts from a separate set of animals were used for histopathology and both transcriptomics and proteomics assessments. Skeletal muscle mass was determined in vivo by body composition measurement using EchoMRI and micro‐CT imaging of lower hindlimb; and ex vivo by weighing the isolated muscles and histopathology. Muscle strength and exercise tolerance was also measured. Whole body metabolism was assessed by Comprehensive Laboratory Animal Monitoring System metabolic cages (CLAMS), oral glucose tolerance test (oGTT), fasting glucose and plasma insulin levels and the homeostasis model assessment of insulin resistance index (HOMA‐IR). Created with Biorender.com.
**Figure S2:** 24‐week‐old mice harbouring cardiac myosin R403Q mutation recapitulate hallmarks of developed HCM. Echocardiography of 24‐week‐old R403Q mice show hallmarks of developed HCM, including left ventricular hypertrophy (A) and associated geometry (C and D), hypercontractility (B) and diastolic dysfunction (E, F). Data shown are mean ± SEM *n* = 6 (WT) and 6 (R403Q). *p* values were obtained by unpaired *t*‐test with Welch's correction. *p < 0.05; ***p < 0.001; ****p < 0.0001.
**Figure S3:** Body total and free water content after 4‐week CDD866 treatment as determined by EchoMRI. Body total (A) and free water (B) content did not change in R403Q mice vs. wild‐type (WT) littermates. A 4‐week CDD866 treatment increased body total water content (A) without changing body free water (B) in R403Q mice. Data shown are mean ± SEM. *n* = 14 (WT‐placebo), 10 (R403Q‐placebo) and 10 (R403Q‐CDD866). *p* values were obtained by one‐way ANOVA with Bonferroni multiple comparisons tests (adjusted *p* value threshold of < 0.05). ***p < 0.001; ns, not significant.
**Figure S4:** Representative 2D and 3D images of segmented muscle of lower‐hindlimb acquired by micro‐CT. A 4‐week CDD866 treatment increased skeletal muscle mass in R403Q mice.
**Figure S5:** Wire hang test to determine muscle strength. There were no significant differences in maximum and average latency (A and C) and maximum and average holding impulse (B and D) after 4 weeks of CDD866 or placebo treatment. Data shown are mean ± SEM. *n* = 14 (WT‐placebo), 10 (R403Q‐placebo) and 10 (R403Q‐CDD866). *p* values were obtained by one‐way ANOVA with Bonferroni multiple comparisons tests (adjusted *p* value threshold of < 0.05). ns, not significant.
**Figure S6:** Screen hang test to determine muscle strength. There were no significant differences in maximum and average latency (A and C) and maximum and average holding impulse (B and D) after 4 weeks of CDD866 or placebo treatment. Data shown are mean ± SEM. *n* = 14 (WT‐placebo), 10 (R403Q‐placebo) and 10 (R403Q‐CDD866). *p* values were obtained by one‐way ANOVA with Bonferroni multiple comparisons tests (adjusted *p* value threshold of < 0.05). ns, not significant.
**Figure S7:** Fasting blood glucose levels. Data shown are mean ± SEM. *n* = 5 (WT‐placebo), 9 (R403Q‐placebo) and 5 (R403Q‐CDD866). *p* values were obtained by one‐way ANOVA with Bonferroni multiple comparisons tests (adjusted *p* value threshold of < 0.05). ns, not significant.
**Figure S8:** Western blot analysis of quadriceps muscle. CDD866 nonsignificantly increased the expression of branched‐chain ketoacid dehydrogenase kinase (BCKDK) in R403Q mice (A), which was associated with a trend for increased phosphorylation of the branched‐chain ketoacid dehydrogenase E1 alpha subunit (BCKDHA) a rate‐limiting step in BCAA catabolism, as the ratio of phosphorylated‐to‐total BCKDHA increased (B). Data shown are mean ± SEM. *n* = 5–6. *p* values were obtained by one‐way ANOVA with Bonferroni multiple comparisons tests (adjusted *p* value threshold of < 0.05). ns, not significant.
**Figure S9:** Respiratory exchange ratio (RER) in metabolic cages. No profound differences in the respiratory exchange ratio (RER) (A, B), a proxy for metabolic fuel utilization, flexibility and efficiency, were found among study groups during 4 days in Comprehensive Laboratory Animal Monitoring System metabolic cages (CLAMS), whereas the RER's physiological circadian oscillation was maintained (A). The RER data at each 54‐min mark were averaged across all animals within the group. Percent differences were calculated using the averaged RER of the full 4 days in WT‐placebo as the control. The absolute value of each resulting data point was taken and subtracted from 100% to represent the percentage of RER compared with the WT‐placebo. Data shown are mean ± SEM. *n* = 4. *p* values were obtained by 2‐way ANOVA with Bonferroni multiple comparisons tests (adjusted *p* value threshold of < 0.05). ns, not significant.
**Figure S10:** Ambulatory activity in metabolic cages. CDD866 did not affect the ambulatory activity of R403Q mice during 4 days in Comprehensive Laboratory Animal Monitoring System metabolic cages (CLAMS) (A, B) while maintaining its circadian oscillation (A). This evidence shows that CDD866‐induced skeletal muscle hypertrophy was not accompanied by a detected increase in ambulatory activity. The ambulatory activity data at each 54‐min mark were averaged across all animals within the group. Percent differences were calculated using the averaged ambulatory activity of the full 4 days in WT‐placebo as the control. The absolute value of each resulting data point was taken and subtracted from 100% to represent the percentage of ambulatory activity compared with the WT‐placebo. Data shown are mean ± SEM. *n* = 4. *p* values were obtained by 2‐way ANOVA with Bonferroni multiple comparisons tests (adjusted *p* value threshold of < 0.05). ***p < 0.001; ****p < 0.0001; ns, not significant.
**Figure S11:** Heart dimensions after 4‐week CDD866 treatment as assessed by echocardiography. R403Q hearts showed decreased left ventricular end‐systolic diameter (ESD) (A) and end‐diastolic diameter (EDD) (B). 4‐week CDD866 treatment improved EDD with a trend for ESD improvement. Data shown are mean ± SEM. *n* = 7 (WT‐placebo), 8 (R403Q‐placebo) and 9 (R403Q‐CDD866). *p* values were obtained by one‐way ANOVA with Bonferroni multiple comparisons tests (adjusted *p* value threshold of < 0.05). **p < 0.01; ***p < 0.001; ****p < 0.0001; ns, not significant.
**Figure S12:** Cardiac proteomics volcano plots. Hearts of 28‐week‐old animals after 4 weeks of placebo (A) or CDD866 (B) treatment were used (*n* = 5–6 animals per group). Proteins with significant differential abundance between R403Q‐placebo and WT‐placebo or between R403Q‐CDD866 and R403Q‐placebo were identified using two‐sided independen*t*‐sample Student's *t*‐tests (*p* < 0.05) for each protein. Volcano plots were generated for each comparison, displaying log2 fold change on the x axis and −log10 raw *p* value on the y axis. Proteins meeting both significance thresholds (*p* < 0.05 and |log2 FC| > 0.3) were highlighted, with upregulated proteins shown in red and downregulated proteins in blue. Mean FC, mean fold change; *p* value, nominal *p* value. *p* < 0.05 was considered significant. Highlighted proteins are based on nominal *p* values, not FDR‐significant proteins.
**Figure S13:** Cardiac proteomics. (A) At nominal *p* < 0.05, 412 proteins differed between R403Q‐placebo and WT‐placebo hearts and 1184 differed between R403Q‐CDD866 and R403Q‐placebo hearts; 119 met this threshold in both comparisons, including 62 with opposite directions of change (Tables S5 and S6). (B) Selected IPA disease/function annotations and upstream regulator predictions for the disease and treatment comparisons. Bars show activation z‐scores; positive and negative scores indicate predicted directions, with most scores having |*z*| < 2. The selected entries met nominal overlap *p* < 0.05. Opposite signs suggest potential counter‐regulation and do not demonstrate restored insulin/AKT signalling or reversal of a diabetic phenotype. IPA inputs used nominal protein‐level *p* < 0.05 for disease and *p* < 0.01 for treatment. No individual proteins survived BH FDR correction, and IPA overlap *p* values were unadjusted; these analyses are exploratory.
**Table S1:** Selected cardiac metabolic pathways depicted in Figure 3B. Hearts of 28‐week‐old animals after 4 weeks of placebo or CDD866 treatment were used (*n* = 5 animals per group). Differential expression was assessed using the Wald test implemented in the DESeq2 R package. Gene sets were obtained from the Molecular Signatures Database (MSigDB), Version 2024.1.Mm. FDR q, Benjamini–Hochberg false discovery rate–corrected *p* value. NES, normalized enrichment score. FDR q < 0.05 was considered significant. Gene sets coordinately upregulated are depicted in red; gene sets coordinately downregulated are depicted in blue.
**Table S2:** Selected gene sets oppositely regulated by CDD866 in R403Q hearts. Hearts of 28‐week‐old animals after 4 weeks of placebo or CDD866 treatment were used (*n* = 5 animals per group). Differential expression was assessed using the Wald test implemented in the DESeq2 R package. Gene sets were obtained from the Molecular Signatures Database (MSigDB), Version 2024.1.Mm. FDR q, Benjamini–Hochberg false discovery rate–corrected *p* value. NES, normalized enrichment score. *p*, nominal *p* value. FDR q < 0.05 was considered significant. Gene sets coordinately upregulated are depicted in red; gene sets coordinately downregulated are depicted in blue.
**Table S3:** Top 20 most upregulated or downregulated gene sets in R403Q hearts and their normalization by CDD866. Hearts of 28‐week‐old animals after 4 weeks of placebo or CDD866 treatment were used (*n* = 5 animals per group). Differential expression was assessed using the Wald test implemented in the DESeq2 R package. Gene sets were obtained from the Molecular Signatures Database (MSigDB), Version 2024.1.Mm. FDR q, Benjamini–Hochberg false discovery rate–corrected *p* value. NES, normalized enrichment score. *p*, nominal *p* value. FDR q < 0.05 was considered significant. Gene sets coordinately upregulated are depicted in red; gene sets coordinately downregulated are depicted in blue.
**Table S4:** Top 20 most upregulated or downregulated gene sets by CDD866 in R403Q hearts. Hearts of 28‐week‐old animals after 4 weeks of placebo or CDD866 treatment were used (*n* = 5 animals per group). Differential expression was assessed using the Wald test implemented in the DESeq2 R package. Gene sets were obtained from the Molecular Signatures Database (MSigDB), Version 2024.1.Mm. FDR q, Benjamini–Hochberg false discovery rate–corrected *p* value. NES, normalized enrichment score. *p*, nominal *p* value. FDR q < 0.05 was considered significant. Gene sets coordinately upregulated are depicted in red; gene sets coordinately downregulated are depicted in blue.
**Table S5:** Oppositely regulated proteins (DOWN in R403Q, UP by CDD866) from the Venn diagram in Figure S13A. Of total 119 overlapping proteins between the effects of R403Q mutation in placebo‐treated animals and CDD866 in R403Q hearts (*n* = 5–6 animals per group), 62 proteins were regulated in opposite directions. Of those 62 oppositely regulated proteins, 47 were downregulated by R403Q mutation and upregulated by CDD866. Proteins with significant differential abundance across comparisons were identified using two‐sided independen*t*‐sample Student's *t*‐tests for each protein. Mean FC, mean fold change; *p* value, nominal *p* value; BH p‐val, Benjamini–Hochberg‐corrected *p* value. *p* < 0.05 was considered significant. Proteins coordinately upregulated are depicted in red; Proteins coordinately downregulated are depicted in blue.
**Table S6:** Oppositely regulated proteins (UP in R403Q, DOWN by CDD866) from the Venn diagram in Figure S13A. Of total 119 overlapping proteins between the effects of R403Q mutation in placebo‐treated animals and CDD866 in R403Q hearts (*n* = 5–6 animals per group), 62 proteins were regulated in opposite directions. Of those 62 oppositely regulated proteins, 15 were upregulated by R403Q mutation and downregulated by CDD866. Proteins with significant differential abundance across comparisons were identified using two‐sided independen*t*‐sample Student's *t*‐tests for each protein. Mean FC, mean fold change; *p* value, nominal *p* value; BH p‐val, Benjamini–Hochberg‐corrected *p* value. *p* < 0.05 was considered significant. Proteins coordinately upregulated are depicted in red; Proteins coordinately downregulated are depicted in blue.
**Table S7:** Top 20 proteins upregulated or downregulated by R403Q in placebo‐treated animals. Hearts of 28‐week‐old animals after 4 weeks of placebo or CDD866 treatment were used (*n* = 5–6 animals per group). Proteins with significant differential abundance across comparisons were identified using two‐sided independen*t*‐sample Student's *t*‐tests for each protein. Mean FC, mean fold change; *p* value, nominal *p* value; BH p‐val, Benjamini–Hochberg‐corrected *p* value. *p* < 0.05 was considered significant. Proteins coordinately upregulated are depicted in red; Proteins coordinately downregulated are depicted in blue.
**Table S8:** Top 20 proteins upregulated or downregulated by CDD866 in R403Q hearts. Hearts of 28‐week‐old animals after 4 weeks of placebo or CDD866 treatment were used (*n* = 5–6 animals per groups). Proteins with significant differential abundance across comparisons were identified using two‐sided independen*t*‐sample Student's *t*‐tests for each protein. Mean FC, mean fold change; *p* value, nominal *p* value; BH p‐val, Benjamini–Hochberg‐corrected *p* value. *p* < 0.05 was considered significant. Proteins coordinately upregulated are depicted in red; Proteins coordinately downregulated are depicted in blue.
**Table S9:** Integrated cardiac RNA and protein upstream regulator analysis. Regulator names in bold identify entries included in Figure 4. IPA activation z‐scores for all 88 regulator entries meeting nominal overlap *p* < 0.05 in both disease and treatment comparisons in each omics layer. No z‐score magnitude or directional‐concordance threshold was applied for inclusion. Disease denotes R403Q‐placebo versus WT‐placebo; CDD866 denotes R403Q‐CDD866 versus R403Q‐placebo. RNA denotes cardiac RNA‐seq; protein denotes cardiac SomaScan proteomics. Overlap *p* values were not adjusted for multiple testing. Rows proceed down the left block and then the right, ordered by directional pattern, with concordant disease‐to‐treatment sign reversals first. Red and blue indicate positive and negative z‐scores, respectively. Bold scores have |*z*| ≥ 2; smaller absolute scores represent weaker directional predictions. NA indicates that the regulator was listed but no activation z‐score was reported. A dagger (†) identifies an IPA directional bias flag in the RNA treatment export. Entries include genes, families, complexes and chemical/perturbation signatures; chemical entries do not imply that the compound was administered or measured. Opposite disease and treatment signs indicate potential counter‐regulation, not complete normalization or causal mediation. No individual SomaScan proteins survived Benjamini–Hochberg FDR correction; the proteomic component and integrated regulator comparisons are exploratory.

**Video S1:** A representative 3D micro‐CT rendering of lower hindlimb muscle volume in WT‐placebo.

**Video S2:** A representative 3D micro‐CT rendering of lower hindlimb muscle volume in R403Q‐placebo.

**Video S3:** A representative 3D micro‐CT rendering of lower hindlimb muscle volume in R403Q‐CDD866.

## Data Availability

Data supporting the findings of this study are available from the corresponding author upon reasonable request. The RNA sequencing and SomaScan proteomics datasets have been deposited in Gene Expression Omnibus (GEO) with the following Series IDs: GSE317212 (RNA sequencing datasets) and GSE326015 (SomaScan proteomics datasets).
