# Supplementary material for: A Murinized Bimagrumab Analogue Increases Skeletal Muscle Mass and Improves Cardiometabolic Remodelling in Experimental Hypertrophic Cardiomyopathy
Source: J Cachexia Sarcopenia Muscle. 2026 Nov 11;17(6):e70420. doi: 10.1002/jcsm.70420 (PMC13752875; doi:10.1002/jcsm.70420)
Supplement: Supplementary file 1 [file JCSM-17-e70420-s001.pdf]

**A murinized bimagrumab analogue increases skeletal muscle mass and improves cardiometabolic remodeling in experimental hypertrophic cardiomyopathy**

Tomas Baka, Sophia M Mirrione, Fuzhong Qin, Adam C Gower, Huamei He, Connor Haney, Kuan Ming Su, Simon T Dillon, Towia A Libermann, Kylie Heitman, Andrew P Ryan, Joseph T Brozinick, Francesca Seta, David R Pimentel, Flora Sam, Ivan Luptak

**Supplementary material**

**Supplementary Methods** pages 2-14

**Supplementary Figures** pages 15-27

**Supplementary Tables** pages 28-38

**Supplementary Videos** page 39

## Supplementary Methods

### Body composition measurement and indirect calorimetry

Body composition was determined by noninvasive quantitative magnetic resonance imaging by using EchoMRI-700 (EchoMRI, Houston, TX, USA). Animals were placed in a thin-walled plastic cylinder and briefly subjected to a low-intensity electromagnetic field to measure body fat, lean mass, free water, and total water [1,2]. Oxygen consumption, carbon dioxide production, respiratory exchange ratio and locomotor activity were measured by a Comprehensive Laboratory Animal Monitoring System (CLAMS; Columbus Instruments, Columbus, OH, USA) consisting of an open-circuit calorimeter and motion detectors. Animals were individually placed in metabolic cages for four consecutive days within a light- and temperature-controlled enclosure and monitored daily for animal welfare [1]. Both body composition (EchoMRI) and indirect calorimetry (CLAMS) were performed in collaboration with the Metabolic Phenotyping Core at Boston University Chobanian and Avedisian School of Medicine, Boston, MA, USA.

### In vivo micro-CT imaging of lower hindlimb to determine skeletal muscle mass

At the end of the study, in vivo micro-CT imaging of the mouse lower hindlimb was performed to determine muscle mass using the Quantum GX2 microCT Imaging System (Revvity, Waltham, MA, USA), as described previously [3,4]. Mice were anesthetized using 1-3% isoflurane. Heart rate and respiration rate were monitored throughout the imaging session. Anesthetized mice were placed prone to the micro-CT instrument leaving the hind limbs in a natural position (with foot-tibia angle of approx. 40°). A region of interest (ROI) depicting the hind limb in the natural position was set with live fluoroscopy at a 36 µm spatial resolution. The ROI of the hind limb was scanned at 90 kV voltage and 88 µA current for 2 minutes. The acquired images were processed and evaluated using Analyze 14.0 software (AnalyzeDirect, Overland Park, KS, USA). To determine skeletal muscle mass, the lower hindlimb muscle

complex was segmented and threshold for the appropriate voxel grey assigned to muscle tissue. The in vivo micro-CT imaging was performed in collaboration with the In Vivo Imaging Core Facility at Boston University Chobanian and Avedisian School of Medicine, Boston, MA, USA.

**Skeletal muscle strength determination**

To measure muscle strength, mice underwent three different tests as follows:

A) Grip strength test: The forelimb grip strength was measured using a grip strength meter, as described previously [5,6]. A mouse was lifted and held by the tail in order to allow the mouse to grasp a wire grid attached to the grip strength meter with the forepaws. Thereafter, the mouse was gently pulled away from the instrument until it released its grip. The maximum force exerted by the mouse before losing its grip was recorded as a measure of grip strength. There were three repetitions of the measurement in one session. The averaged value of the three repetition is presented as the grip strength of the mouse.

B) Screen hang test: The mouse was placed on a metal mesh (1 mm diameter wires with 1 cm × 1 cm spacing) and allowed to grip the mesh with all four paws [5,7]. Thereafter, the metal mesh was slowly turned upside down and held horizontally 35 cm above a thick layer of soft bedding (such as foam or cotton balls). The time until the mouse falls from the metal mesh served as a measure of muscle strength. After each fall, the mouse was allowed to recover for 1 minute. There were three repetitions of the measurement in one session. The averaged value of the three repetition is presented as the grip strength of the mouse. Maximum and average latency (time-to-fall) were measured and maximum and average holding impulses were calculated as: latency x body weight.

C) Wire hang test: The mice were allowed to grasp by their two forelimbs a 2-mm diameter metal wire maintained horizontally 35 cm above a thick layer of soft bedding (such as foam or cotton balls) [8,9]. The time until the mouse falls from the wire served as a measure

of muscle strength. After each fall, the mouse was allowed to recover for 1 minute. There were three repetitions of the measurement in one session. The averaged value of the three repetition is presented as the grip strength of the mouse. Maximum and average latency (time-to-fall) were measured and maximum and average holding impulses were calculated as: latency x body weight.

**Exercise tolerance test**

Exercise tolerance was measured using a rodent treadmill equipped with an air puff motivator (Columbus Instruments, Columbus, OH, USA), as described previously [10,11]. The treadmill was set at a constant incline of 10° and the initial speed of the treadmill belt was 15 m/min and was increased by 3 m/min every 2 minutes. Animals were run to exhaustion and the total exercise time was converted to distance. At exhaustion, the animal could jump off the moving belt onto a stationary platform. Exhaustion was defined as the point when the animal couldn't keep pace with the treadmill and no longer avoided the air puff motivator (>5 sec in air stream). The exhaustion was determined by an observer blinded to group identity who controls the speed and the air puff motivator manually. Each animal was run separately. Mice underwent acclimation sessions in order to acclimate to treadmill environment and handling. Mice that failed to acclimate or did not run were excluded from the experiment.

**Blood glucose, insulin and HOMA-IR determination**

Fasting blood glucose was measured by a handheld AimStrip Plus glucometer (Germaine Laboratories, San Antonio, TX, USA) after 12-h fasting, using specific test strips conforming to the manufacturer's instructions and as described previously [12,13]. Fasting plasma insulin was measured with an Ultra-Sensitive Mouse Insulin ELISA Kit (Catalog No. 90080, Crystal Chem, Elk Grove Village, IL, USA). To obtain plasma, whole blood was collected to lithium heparin blood collection tubes (Catalog No. 365965, BD MicroTainer,

Becton Dickinson, Franklin Lakes, NJ, USA) and centrifuged at 3000g and 4°C for 15 min. Subsequently, the homeostasis model assessment of insulin resistance index (HOMA-IR) was calculated using the formula: (fasting blood glucose [mg/dL]×fasting plasma insulin [μU/mL])÷405, as described previously [12].

**Oral glucose tolerance test (oGTT)**

After 12-h fasting, a bolus of 2 g glucose (Glucose, G8270, Sigma-Aldrich, St. Louis, MO, USA) per 1 kg body weight was administered by oral gavage, as described previously [14,15]. Blood glucose, was measured serially at 0, 15, 30, 60, 90, 120 and 180 minutes after glucose bolus by a handheld AimStrip Plus glucometer (Germaine Laboratories, San Antonio, TX, USA). Repeated blood sampling was performed by gently removing the clot/scab after tail vein nicking. Subsequently, the area under the curve (AUC) was calculated using GraphPad Prism 10 (GraphPad Software, La Jolla, CA).

**Echocardiography**

For data capture and analysis, a VisualSonics Vevo 3100 high-resolution imaging system equipped with a 22- to 55-MHz MS550D transducer and VevoLab offline analytic software (Toronto, Canada) were used. Mice were anesthetized with isoflurane at a concentration of 2.5% for anaesthesia induction and then 1.5% for anaesthesia maintenance. To assess LV dimensions and systolic function, the heart was imaged in the 2-D parasternal short-axis view, and an M-mode echocardiogram of the mid-ventricle was recorded at the level of papillary muscles. LV anterior (AWT) and posterior wall thickness (PWT), LV end-diastolic (EDD) and end-systolic (ESD) dimensions were measured, and total wall thickness and LV fractional shortening (FS = (EDD–ESD)/EDD × 100) were calculated. To assess LV diastolic function, pulsed-wave Doppler images were collected in the apical four-chamber view to record the mitral Doppler flow spectra and measure peak early (E) and late (A) mitral inflow

velocities and calculate E/A ratio; tissue Doppler images were collected in the parasternal short-axis view to measure myocardial peak early diastolic velocity (Em) [12,13,16].

### Determination of $^{13}$C-substrate oxidation isolated beating hearts

To determine substrate oxidation rates, hearts were perfused with $^{13}$C-enriched fatty acids (U-$^{13}$C labeled fatty acid mix, Cambridge Isotope, CLM-8455, 0.4 mM) and 1-$^{13}$C labeled glucose (Cambridge Isotope, CLM-420, 5.5 mM) [13,17]. Proton-decoupled $^{13}$C NMR spectra of cardiac tissue extract were acquired with a 600 MHz magnet at Harvard Medical School Bio-Molecular MR Facility. The contributions of each substrate to the oxidative metabolism and the rate of anaplerosis were determined using the $^{13}$C isotopomers peak areas of the C3 and C4 of glutamate by modeling the tricarboxylic acid cycle fluxes as previously described [13,18–20]. $^{13}$C-labelled lactate was measured in the perfusate to determine the fate of pyruvate using proton-decoupled $^{13}$C NMR.

### Muscle weight, and heart and skeletal muscle histology and western blot analyses

Mice were euthanized; organs (including heart) and skeletal muscles (m. quadriceps, gastrocnemius and soleus) were harvested and weighed at the end of the study.

LV samples were fixed in fixed in 10% buffered formalin, embedded in paraffin, sectioned, and stained. Myocyte cross-sectional area, and fibrosis were measured and analyzed, as previously described [12,13,16]. To assess myocyte cross-sectional area, sections were stained with hematoxylin and eosin and examined under a light microscope. Five random fields from two sections per animal were analyzed and 50 myocytes per animal were quantified. The cardiomyocyte cross-sectional area was calculated using ImageJ software (National Institutes of Health, Bethesda, MD, USA). To assess fibrosis, sections were

stained with Masson's trichrome and examined under a light microscope. Mean percentage of fibrosis was determined from 10 random fields per heart and quantified using ImageJ software.

OCT-embedded m. quadriceps tissue samples were sectioned transversely at 10–12 µm using a Leica CM3050S cryostat at −20°C and mounted on glass slides (USDeRd #12550400). Sections were air-dried at room temperature for 30 minutes, circled with a PAP pen, and washed twice for 5 minutes in PBS with 0.1% Triton X-100. Blocking was performed for 1 hour in 5% goat serum with Mouse-on-Mouse blocking reagent (Vector Laboratories #2213) in PBS, followed by two 2-minute washes in PBS. Sections were incubated overnight at 4°C with laminin (Sigma-Aldrich #L9393, 1:1000 dilution), diluted in 0.5% BSA in PBS. The following day, sections were washed three times in PBS and incubated for 1 hour at room temperature with secondary antibody goat anti-rabbit IgG-Alexa647 (Thermo Fisher). After final washes, slides were mounted using VECTASHIELD Plus Antifade Mounting Medium (Vector #H1400) and cover slip (Surgipath #3800145ACS). Slides were dried overnight at room temperature before imaging. Fluorescent Images were acquired using a 3dhistech panoramic 250 slide scanner (Version 4.10) and Plan-Apochromat 20x Olympus lens with spatial resolution of 0.325 micrometer/pixel. Tile Scanned Images were acquired in sequence using Cy5Q (Quad filter), TRITC or TxRed, FITC, and DAPI filter minimizing saturated pixels and optimized for sCMOS (Scientific Complementary Metal-Oxide Semiconductor) camera dynamic range. Z focus positions were determined using the Cy5Q/laminin image prior to initial tile scan. Raw proprietary files MRXS were converted using slide master (2.6.0) to individual Tiff format for each channel. Tiff images were then converted in Zeiss arivis pro SIS converter 4.1.0 for cell segmentation using the Cy5Q/laminin channel. Sis Files were opened and batch segmented using Arivis Pro 4.2.2. Generalist Cellpose-based segmentation model Cyto 2 was utilized, and parameters were adjusted 166 µm Cell Diameter, Mask threshold .1. Total Individual Fiber Area measurements were exported and XY plotted Percent Frequency (Relative as Percentages) versus Area in 500 µm bins using Column Analysis for each

treatment group, Frequency Distribution in GraphPad Prism Version 10.4.2. All imaging and quantification were performed by a blinded investigator [21].

For western blot analysis, frozen quadriceps muscle samples were homogenized in buffer containing 50 mM Tris HCl, pH 7.5, 1 mM EDTA, 1 mM EGTA, 10% glycerol, 1% Triton-X, 50 mM sodium fluoride, and 5 mM sodium pyrophosphate with Halt protease and phosphatase inhibitor cocktail (Thermo Scientific). Protein content was determined using a BCA protein assay kit (Pierce, Rockford, IL, USA). Equal amounts of protein (10-20 µg) were separated by SDS-PAGE using precast stain-free gels (Bio-Rad) then transferred to nitrocellulose membranes. Primary and secondary antibody details are listed in a table below. Bands of interest were detected using fluorescent secondary antibodies on a LiCor Odyssey M (LiCor, Lincoln, Nebraska USA) and band intensities were normalized to loading control of beta-actin using Empiria Studio Software.

| Primary Antibody | Manuf-acturer | Product Number | Host | Primary Dilution | Secondary Antibody | Product Number | Secondary Detection |
|---|---|---|---|---|---|---|---|
| BCKDK | Abnova | H00010295-A01 | Mouse | 1:1000 | IRDye® 680LT Goat anti-Mouse IgG | 926-68020 | 1:10000 |
| BCKDH | Affinity Bio-reagent | DF13663 | Rabbit | 1:1000 | IRDye® 800CW Goat anti-Rabbit IgG | 926-32211 | 1:10000 |
| pBCKDH | Cell Signal | 40368 | Rabbit | 1:1000 | IRDye® 800CW Goat anti-Rabbit IgG | 926-32211 | 1:10000 |

## Cardiac RNA isolation, RNA sequencing, and sequencing analysis

RNA isolation, RNA sequencing, and sequencing analysis were performed as described previously [12,13]. Briefly, frozen LV tissue was homogenized, and mRNA was extracted using the RNeasy Universal Mini Kit (Catalog No. 73404, Qiagen, Hilden, Germany)

with DNase digestion using the RNase-Free DNase Set (Catalog No. 79254, Qiagen). The Boston University Microarray and Sequencing Resource Core Facility performed RNA quantity and quality assessment, RNA sequencing, library preparation, and analyses including Gene set enrichment analysis. Sequencing libraries were generated using Illumina mRNA with poly(A) selection and run on an Illumina NextSeq 2000 P3 platform (1.1B reads) with 100x100bp paired-end reads. FASTQ files were aligned to mouse genome build mm10 using STAR (version 2.7.9a). Ensembl-Gene-level counts for non-mitochondrial genes were generated using featureCounts (Subread package, version 2.0.3) and Ensembl annotation build 100 (uniquely aligned reads, same strand). FASTQ quality was assessed using FastQC (version 0.11.7), and alignment quality was assessed using RSeQC (version 3.0.0).

Variance-stabilizing transformation (VST) was accomplished using the varianceStabilizingTransformation function in the DESeq2 R package (version 1.32.0). Principal Component Analysis (PCA) was performed using the prcomp R function with VST expression values that were z-normalized (set to a mean of zero and a standard deviation of one) across all samples within each gene. Differential expression was assessed using the Wald test implemented in the DESeq2 R package. Correction for multiple hypothesis testing was accomplished using the Benjamini-Hochberg false discovery rate (FDR), with FDR $q < 0.05$ considered significant. All sequencing analyses were performed using the R environment for statistical computing (version 4.1.2).

Gene Set Enrichment Analysis (GSEA) (version 2.2.1) was used to identify biological terms, pathways, and processes that are coordinately upregulated or downregulated within each pairwise comparison. The Entrez Gene identifiers of all genes in the Ensembl Gene annotation were ranked by the Wald statistic computed for each pairwise comparison. Each ranked list was then used to perform pre-ranked GSEA analyses (default parameters with random seed 1234) using the Entrez Gene versions of the MH (Hallmark), M2 CP (Biocarta, Reactome, WikiPathways), and M5 (Gene Ontology, GO) gene sets obtained from the Molecular Signatures Database (MSigDB), version 2024.1.Mm.

To identify candidate regulatory programs associated with the R403Q phenotype and CDD866 treatment, cardiac RNA-seq differential-expression results were analyzed using the Upstream Regulator Analysis module of Ingenuity Pathway Analysis (IPA; QIAGEN), version 1.24.1. Analyses were performed separately for R403Q-placebo versus WT-placebo and R403Q-CDD866 versus R403Q-placebo. For both comparisons, gene selection was performed using an FDR (q-value) threshold of 0.05; no fold-change cutoff was applied. Expression fold change was used as the measurement for analysis to calculate directionality (z-scores). The Ingenuity Knowledge Base (Genes Only) was used as the reference set (i.e., population of genes to consider for p-value calculations). Both direct and indirect relationships were considered. All other pre-analysis filtering was performed according to default IPA settings. IPA evaluated the overlap between input genes and known targets of each regulator using a right-tailed Fisher's exact test and calculated activation z-scores based on agreement between observed target-gene expression changes and curated regulatory relationships. Regulator overlap p-values were not adjusted for multiple testing; nominal $p<0.05$ was used for exploratory interpretation. Positive and negative z-scores indicated predicted directions toward activation and inhibition, respectively. Absolute z-scores ≥2 were considered stronger directional predictions, whereas smaller values were interpreted as directional trends. IPA predictions carrying a directional-bias flag were identified and interpreted cautiously. For integration with cardiac proteomics, regulator overlap p-values and activation z-scores were compared across the corresponding disease and treatment contrasts. Selected shared regulators and related regulatory programs were summarized in Figure 4 to illustrate concordant patterns across platforms. Opposite z-score signs between disease and treatment comparisons were interpreted as potential counter-regulation rather than evidence of complete normalization. These analyses were considered exploratory and hypothesis-generating.

**Cardiac SomaScan proteomic analysis**

Whole cell lysates from tissue samples obtained from isolated frozen mouse hearts were generated in T-PER (Thermo) supplemented with 1x HALT protease inhibitor cocktail using a TissueLyser II (Qiagen). 2.4 µg of soluble heart tissue lysate per sample were then analyzed by SomaScan proteomics analysis in the standard 96-well plate assay format on a Tecan Fluent 780 liquid handling robot at the BIDMC Genomics, Proteomics, Bioinformatics and Systems Biology Center. The 11k SomaScan v5.0 Assay-Human Cell & Tissue Kit (Product Code 900-0057) that measures expression of 10,778 proteins using highly selective single-stranded modified Slow Off-rate Modified DNA Aptamers (SOMAmer) was employed according to the manufacturer's standard protocol (SomaLogic; Boulder, CO). The details of the protocol and of the SomaScan platform are described elsewhere [22,23]. Three kit provided pooled plasma Calibrator replicates and three no protein background controls were used to normalize and calibrate the samples on a single assay plate run. Twelve hybridization controls and 296 non-human SOMAmers were added alongside the 10,778 SOMAmers to control for readout variability for each set of test samples. Sample to sample variability was further controlled by several hybridization spike-in controls. Hybridization normalization, plate scaling, median normalization, and calibration of the SomaScan data, performed according to the standard quality control (QC) protocols at SomaLogic, demonstrated that all samples passed the established QC criteria and were fit for further analysis.

Identification of Proteomic Signatures: Proteins with significant differential abundance between R403Q-placebo and WT-placebo or between R403Q-CDD866 and R403Q-placebo were identified using two-sided independent-samples Student's t-tests ($p<0.05$) for each protein [24]. Correction for multiple hypothesis testing was performed using the Benjamini-Hochberg false discovery rate (FDR) method. Due to the limited sample size, no individual proteins remained significant at FDR-adjusted $p<0.05$. Therefore, nominal p-values were used for exploratory visualization and downstream analyses. Mean and median fold change (FC) of a protein between R403Q-placebo and WT-placebo and R403Q-CDD866 and R403Q-placebo

was calculated [25]. If a protein exhibited a lower signal value in the treatment group, then the corresponding FC was represented using a negative sign. For example, a 2-fold downregulation in the treatment group, i.e., a calculated FC of 0.5, is represented as -2.

Volcano plots were generated for each comparison, displaying log2 fold change on the x-axis and −log10 raw p-value on the y-axis. Proteins meeting both significance thresholds ($p < 0.05$ and $|\log2\ FC| > 0.3$) were highlighted, with upregulated proteins shown in red and downregulated proteins in blue. The top 20 most significant proteins passing both thresholds were labeled by gene symbol. Venn diagrams were constructed to visualize the overlap of significant proteins between the two comparisons at the raw p-value ($p<0.05$) level using InteractiVenn (https://www.interactivenn.net/) [26].

Software: All analyses were conducted in Python 3 using pandas for data manipulation, SciPy for statistical testing, statsmodels for FDR correction, and matplotlib for visualization.

Systems Biology Analysis: To assess for potential pathophysiological pathways underlying the differentially expressed mouse heart protein signatures and to more precisely understand the complex interactions between the differentially expressed proteins, we performed functional category, canonical pathway, interactive network, and upstream regulator analyses of all dysregulated proteins upon treatment, with a P value <0.05 for the R403Q-placebo vs WT-placebo and with a P value <0.01 for R403Q-CDD866 vs R403Q-placebo, using the Ingenuity Pathway Analysis (IPA) software tool (QIAGEN, Redwood City, CA), a repository of biologic interactions and functions created from millions of individually modeled relationships that range from the molecular (proteins, genes) to organism (diseases) level.

## Statistical analysis

Results are presented as mean±SEM (unless stated otherwise in figure legend). One-way ANOVA with Bonferroni multiple comparisons test (adjusted *p*-value threshold <0.05) was used to analyze all data except for transcriptomics, proteomics, oral glucose tolerance test

and metabolic cage data. Specific statistical tests and n per group are indicated in figure legends. The statistical analyses were performed using GraphPad Prism 10 (GraphPad Software, La Jolla, CA). $P<0.05$ was considered significant. In the Results section, the percent changes between groups were calculated from group means, with SEM derived using standard propagation formulas.

The oral glucose tolerance test data were analysed using a mixed-effects analysis with Bonferroni multiple comparisons test (adjusted *p*-value threshold $<0.05$). The area under the curve (AUC) was calculated using GraphPad Prism 10 (GraphPad Software, La Jolla, CA) and analysed using one-way ANOVA with Bonferroni multiple comparisons test (adjusted *p*-value threshold $<0.05$).

The CLAMS metabolic cage data were analyzed as follows: The RER and ambulatory activity data at each 54-minute mark were averaged across all animals within the group. Percent differences were calculated using the averaged RER or ambulatory activity data of the full 4 days in WT-placebo as the control. The absolute value of each resulting data point was taken, and subtracted from 100% to represent the percentage of RER or ambulatory activity compared to the WT-placebo. *p*-values were obtained by 2-way ANOVA with Bonferroni multiple comparisons tests (adjusted *p*-value threshold $<0.05$).

**Dataset deposition**

The RNA sequencing and SomaScan proteomics datasets have been deposited in Gene Expression Omnibus (GEO) with Series ID GSE317212 for RNAseq data; and GSE326015 for SomaScan proteomics data. The data that support the findings of this study are available from the corresponding author upon reasonable request.

## Statement on the use of artificial intelligence

In the graphical summary (Figure 5), the schematic illustrations of skeletal muscle, heart, mitochondrion, antibody and treadmill mouse were generated using OpenAI's image-generation tool (September 2026) under author direction. The authors reviewed the illustrations for accuracy and take responsibility for the final figure. These illustrations are conceptual and do not represent experimental images.

## Supplementary Figures

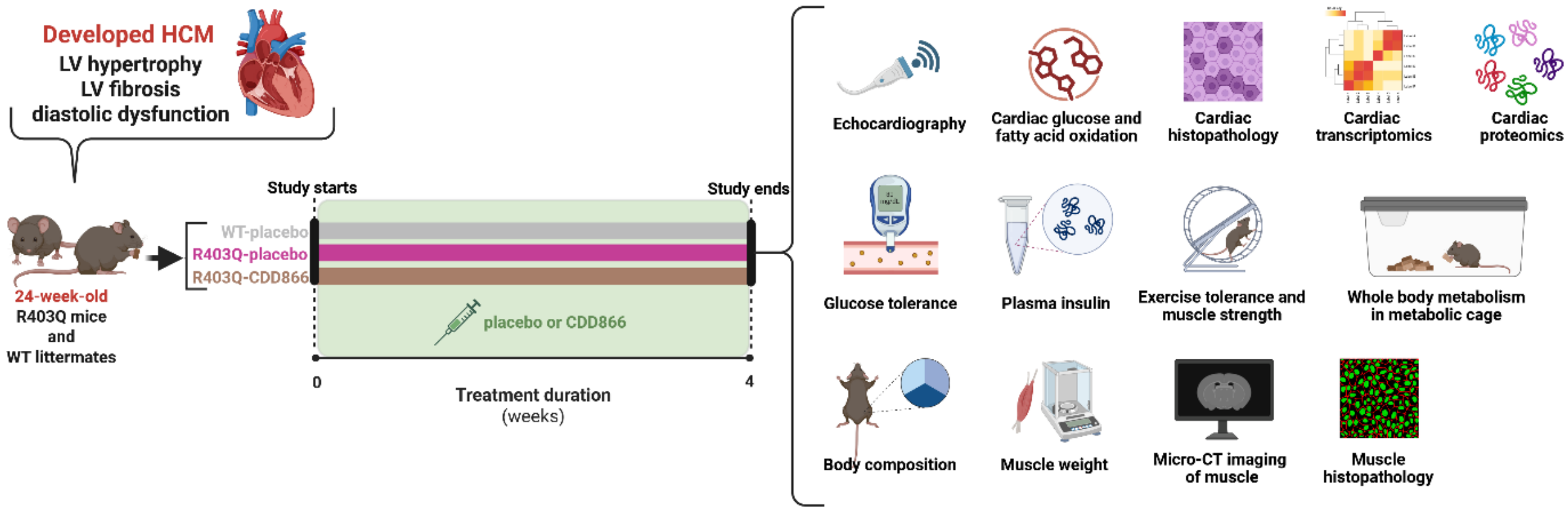


**Figure S1. Study protocol.** 24-week-old male mice harbouring R403Q mutation in cardiac myosin heavy chain or their wild-type littermates were randomized by body weight into three groups: (i) wild-type-placebo (WT-placebo); (ii) R403Q-placebo; and (iii) R403Q-CDD866. At 24 weeks of age, male mice with the cardiac myosin R403Q mutation recapitulate hallmarks of developed hypertrophic cardiomyopathy (HCM) including left ventricular (LV) hypertrophy, fibrosis and diastolic dysfunction. At this age, mice were treated with the antibody CDD866 (murinized bimagrumab) targeting the myostatin/activin receptor ActRII or isotype antibody serving as placebo (20 mg/kg, subcutaneously, every 3 days) for 4 weeks. At the end of the 4-week treatment period, mice underwent echocardiography and heart isolation to determine $^{13}C$-substrate oxidation. Hearts from a separate set of animals were used for histopathology, and both transcriptomics and proteomics assessments. Skeletal muscle mass was determined *in vivo* by body composition measurement using EchoMRI and micro-CT imaging of lower hindlimb; and *ex vivo* by weighing the isolated muscles and histopathology. Muscle strength and exercise tolerance was also measured. Whole body metabolism was assessed by Comprehensive Laboratory Animal Monitoring System metabolic cages (CLAMS), oral glucose tolerance test (oGTT), fasting glucose and plasma insulin levels and the homeostasis model assessment of insulin resistance index (HOMA-IR). Created with Biorender.com.

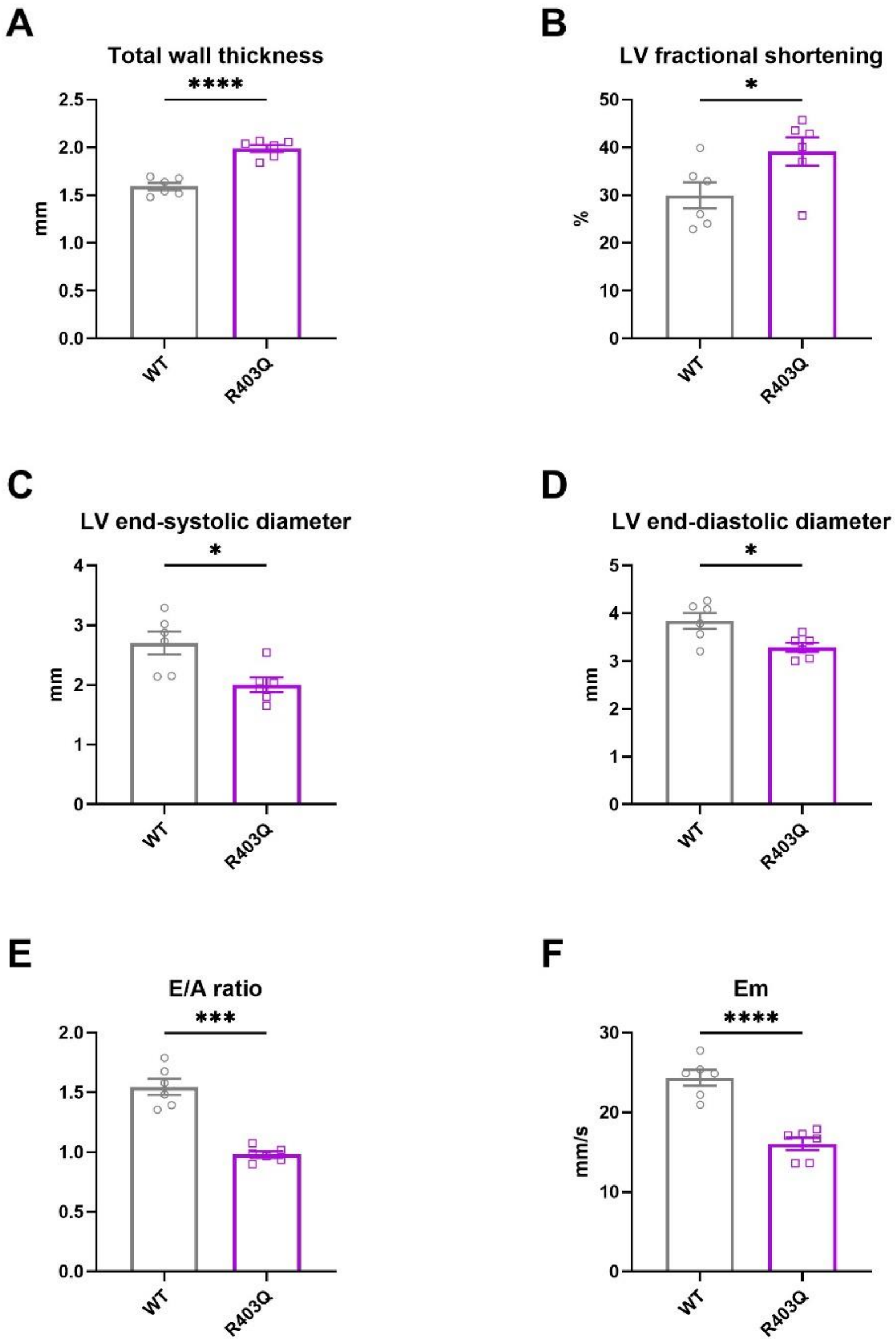


**Figure S2. 24-week-old mice harbouring cardiac myosin R403Q mutation recapitulate hallmarks of developed HCM.** Echocardiography of 24-week-old R403Q mice show hallmarks of developed HCM, including left ventricular hypertrophy (**A**) and associated geometry (**C** and **D**), hypercontractility (**B**) and diastolic dysfunction (**E** and **F**). Data shown are mean±SEM. n = 6 (WT) and 6 (R403Q). *p*-values were obtained by unpaired t test with Welch's correction. **p*<0.05; ****p*<0.001; *****p*<0.0001.

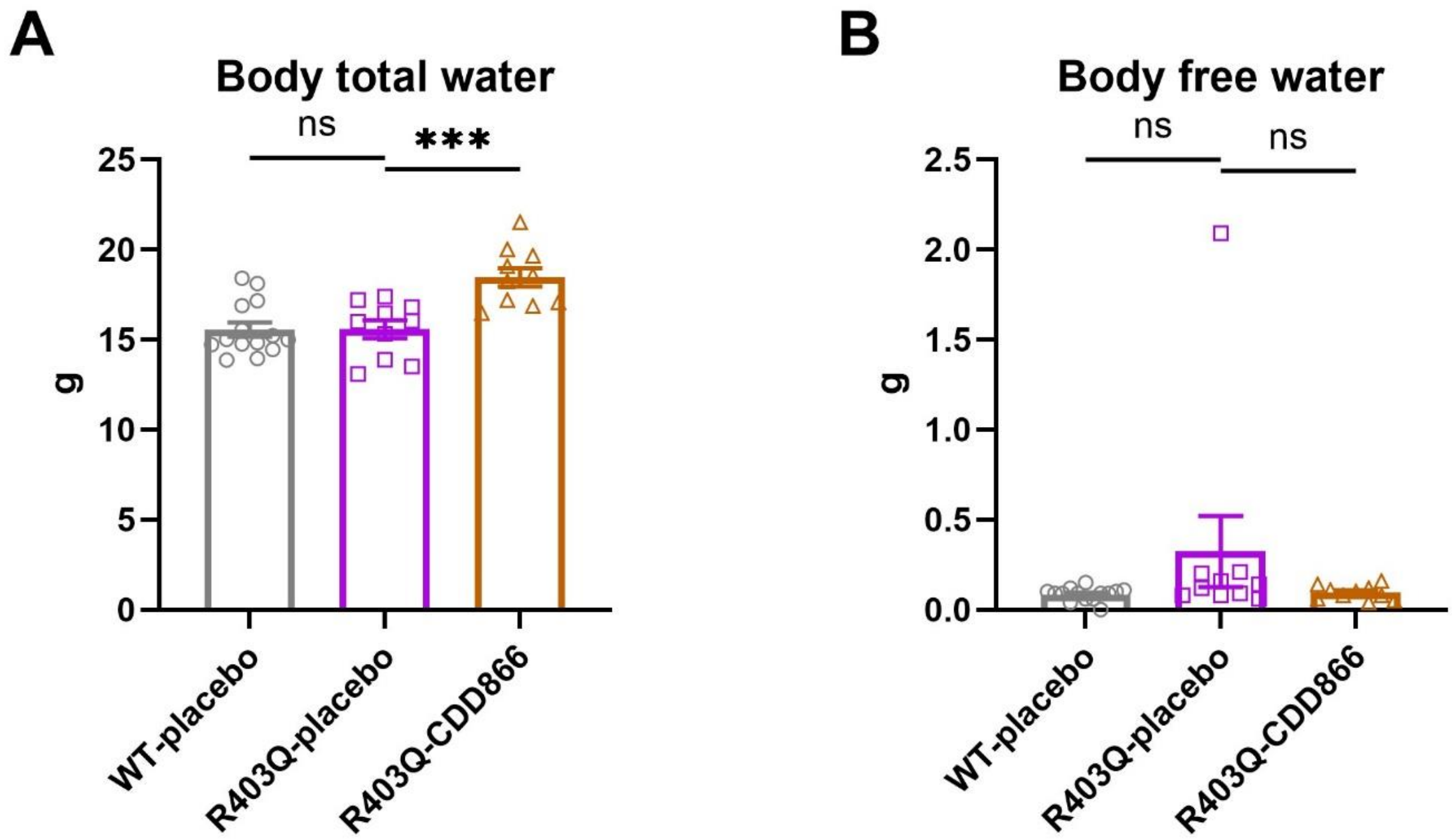


**Figure S3. Body total and free water content after 4-week CDD866 treatment as determined by EchoMRI.** Body total (**A**) and free water (**B**) content did not change in R403Q mice vs wild-type (WT) littermates. A 4-week CDD866 treatment increased body total water content (**A**) without changing body free water (**B**) in R403Q mice. Data shown are mean±SEM. n = 14 (WT-placebo), 10 (R403Q-placebo), and 10 (R403Q-CDD866). *p*-values were obtained by one-way ANOVA with Bonferroni multiple comparisons tests (adjusted *p*-value threshold <0.05). ***$p$<0.001; ns, not significant.

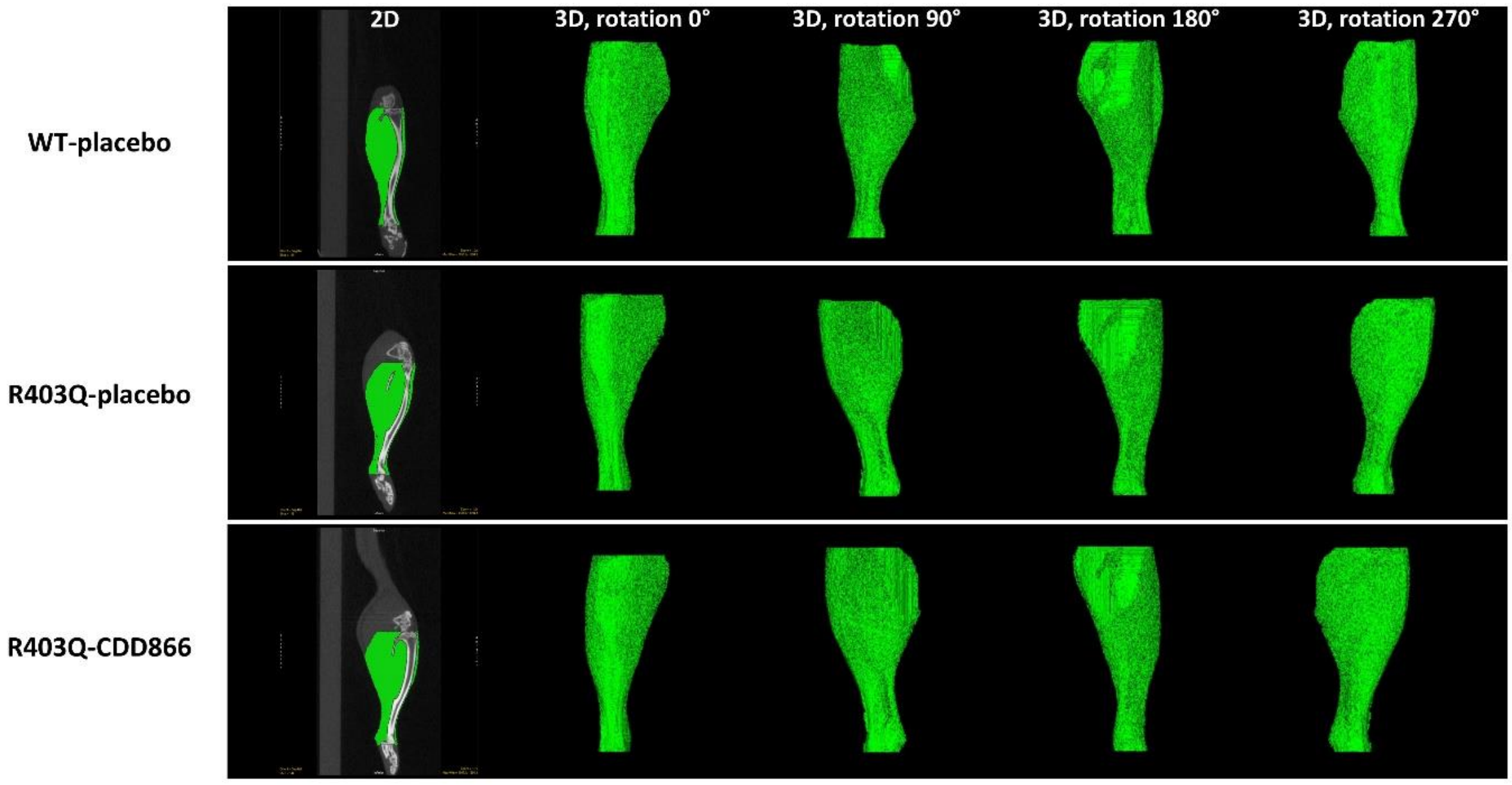


**Figure S4. Representative 2D and 3D images of segmented muscle of lower-hindlimb acquired by micro-CT.** A 4-week CDD866 treatment increased skeletal muscle mass in R403Q mice.

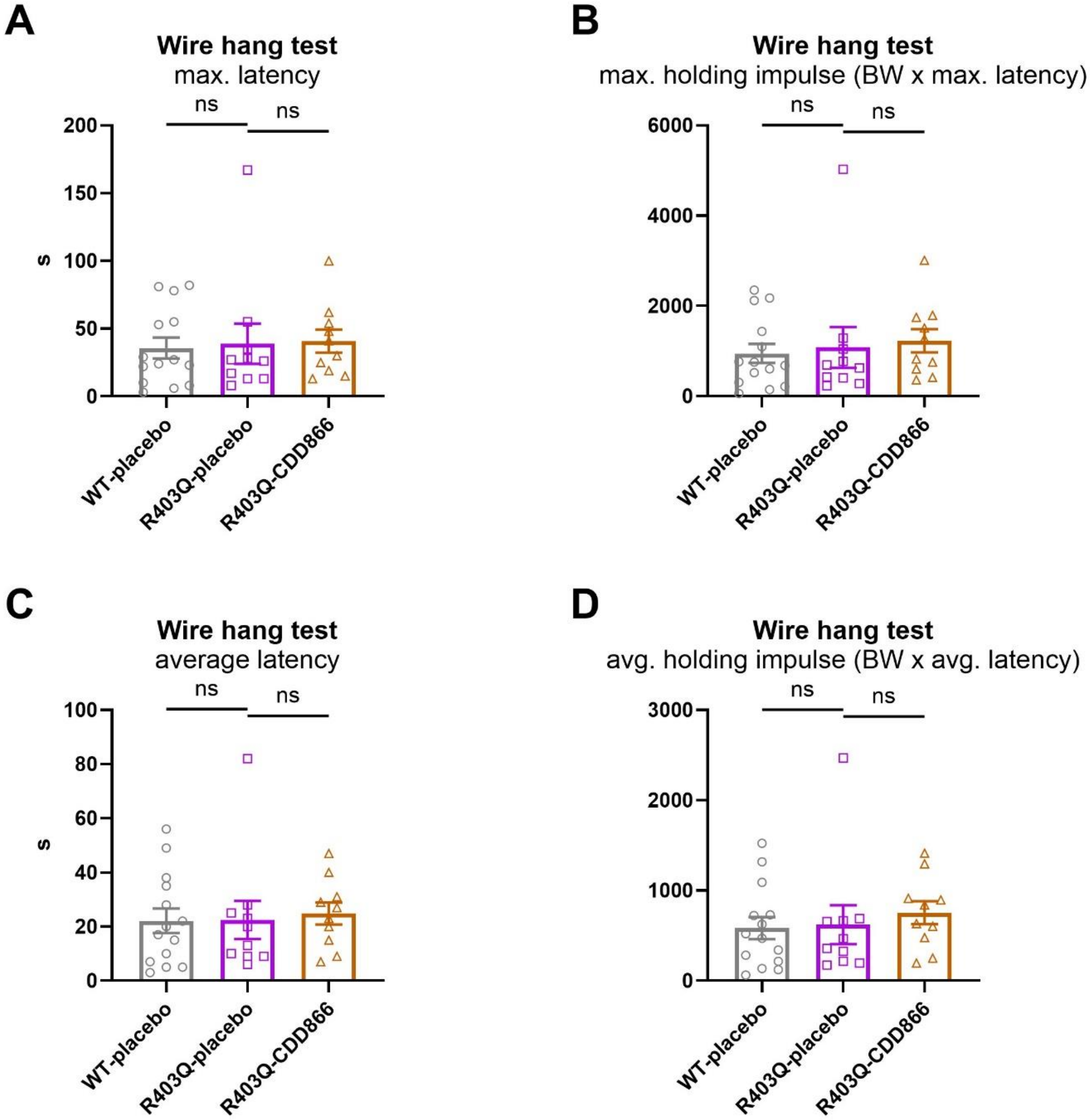


**Figure S5. Wire hang test to determine muscle strength.** There were no significant differences in maximum and average latency (**A** and **C**), and maximum and average holding impulse (**B** and **D**) after 4 weeks of CDD866 or placebo treatment. Data shown are mean±SEM. n = 14 (WT-placebo), 10 (R403Q-placebo), and 10 (R403Q-CDD866). *p*-values were obtained by one-way ANOVA with Bonferroni multiple comparisons tests (adjusted *p*-value threshold <0.05). ns, not significant.

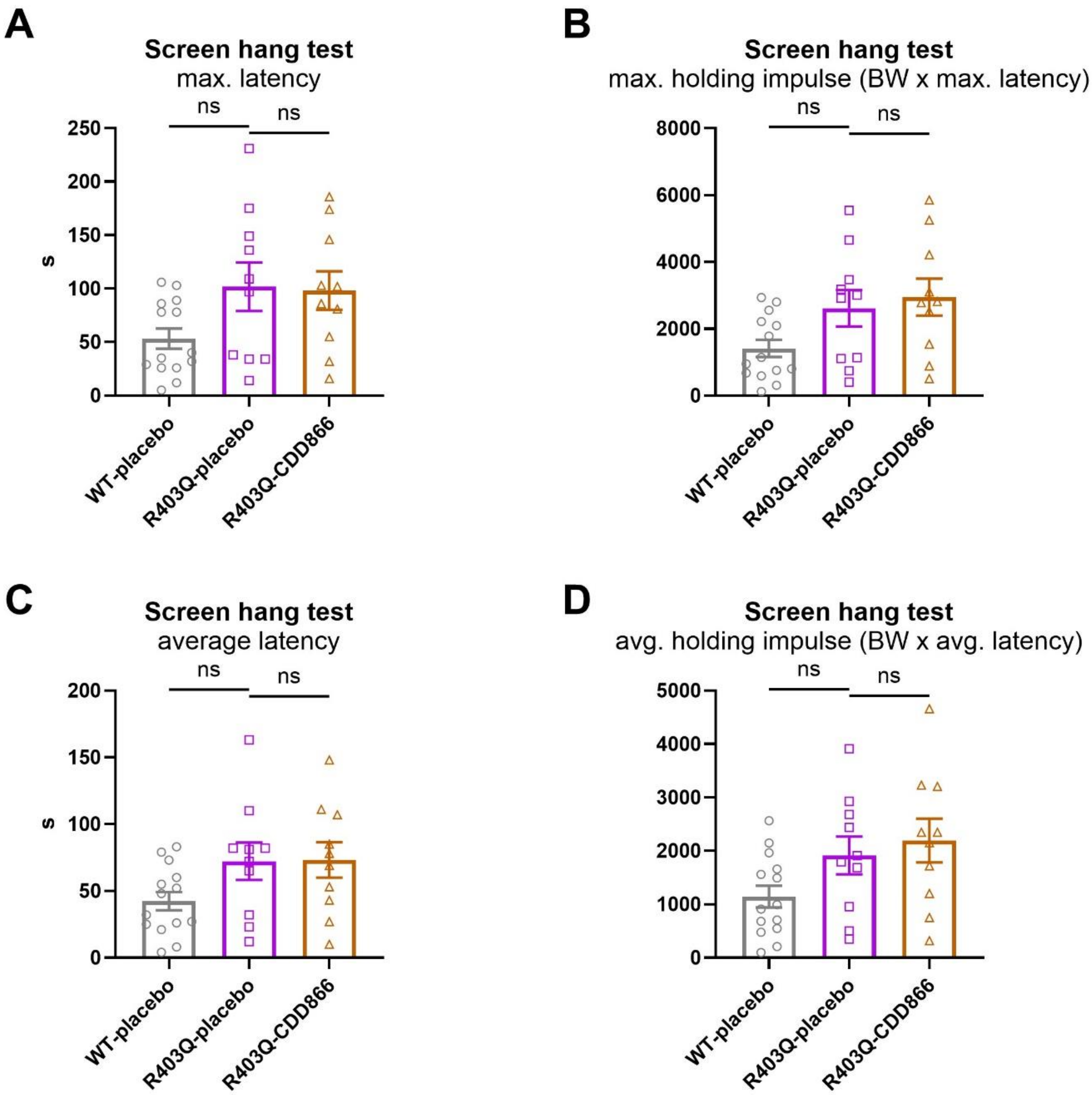


**Figure S6. Screen hang test to determine muscle strength.** There were no significant differences in maximum and average latency (**A** and **C**), and maximum and average holding impulse (**B** and **D**) after 4 weeks of CDD866 or placebo treatment. Data shown are mean±SEM. n = 14 (WT-placebo), 10 (R403Q-placebo), and 10 (R403Q-CDD866). *p*-values were obtained by one-way ANOVA with Bonferroni multiple comparisons tests (adjusted *p*-value threshold <0.05). ns, not significant.

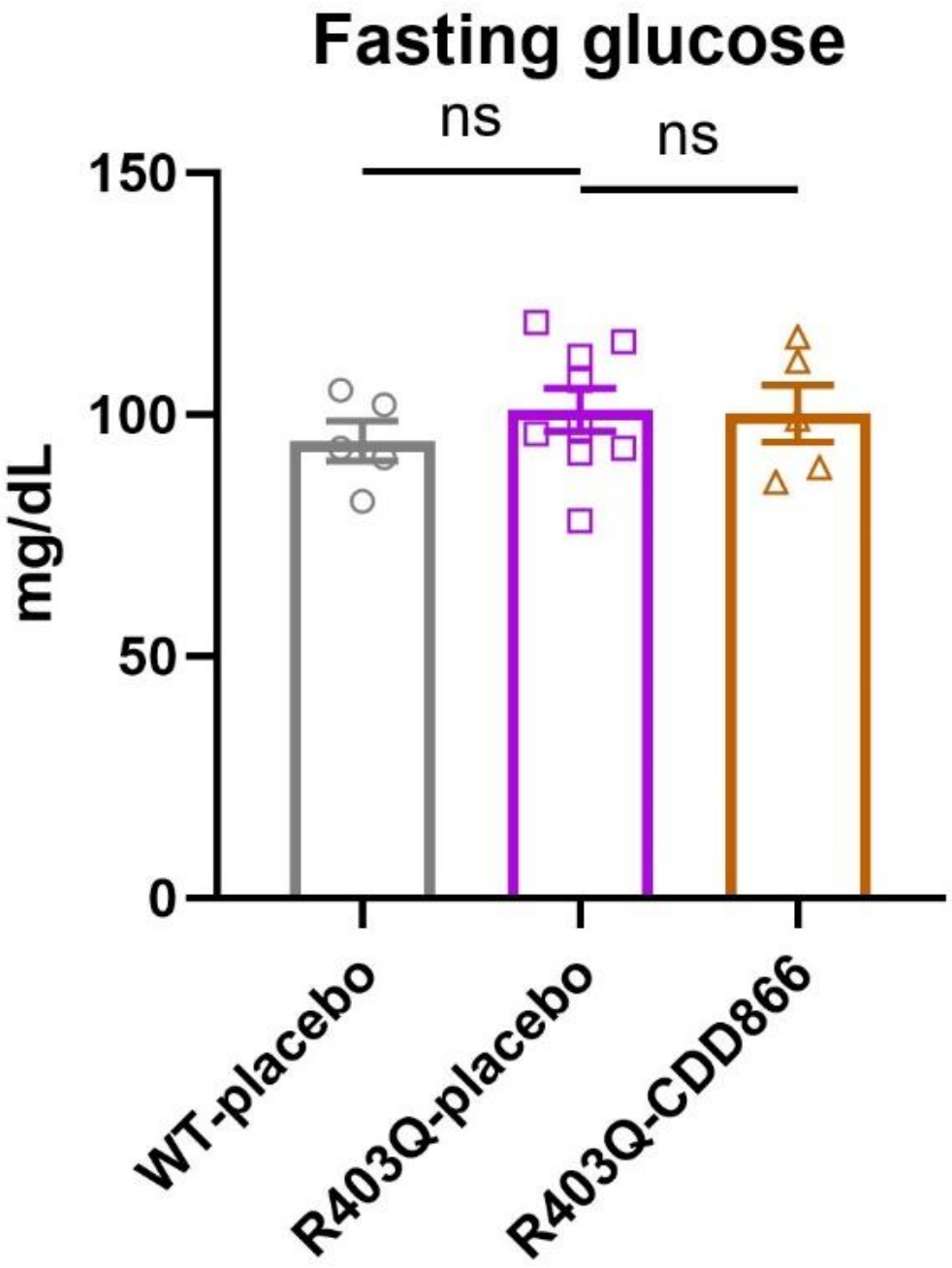


**Figure S7. Fasting blood glucose levels.** Data shown are mean±SEM. n = 5 (WT-placebo), 9 (R403Q-placebo), and 5 (R403Q-CDD866). *p*-values were obtained by one-way ANOVA with Bonferroni multiple comparisons tests (adjusted *p*-value threshold <0.05). ns, not significant.

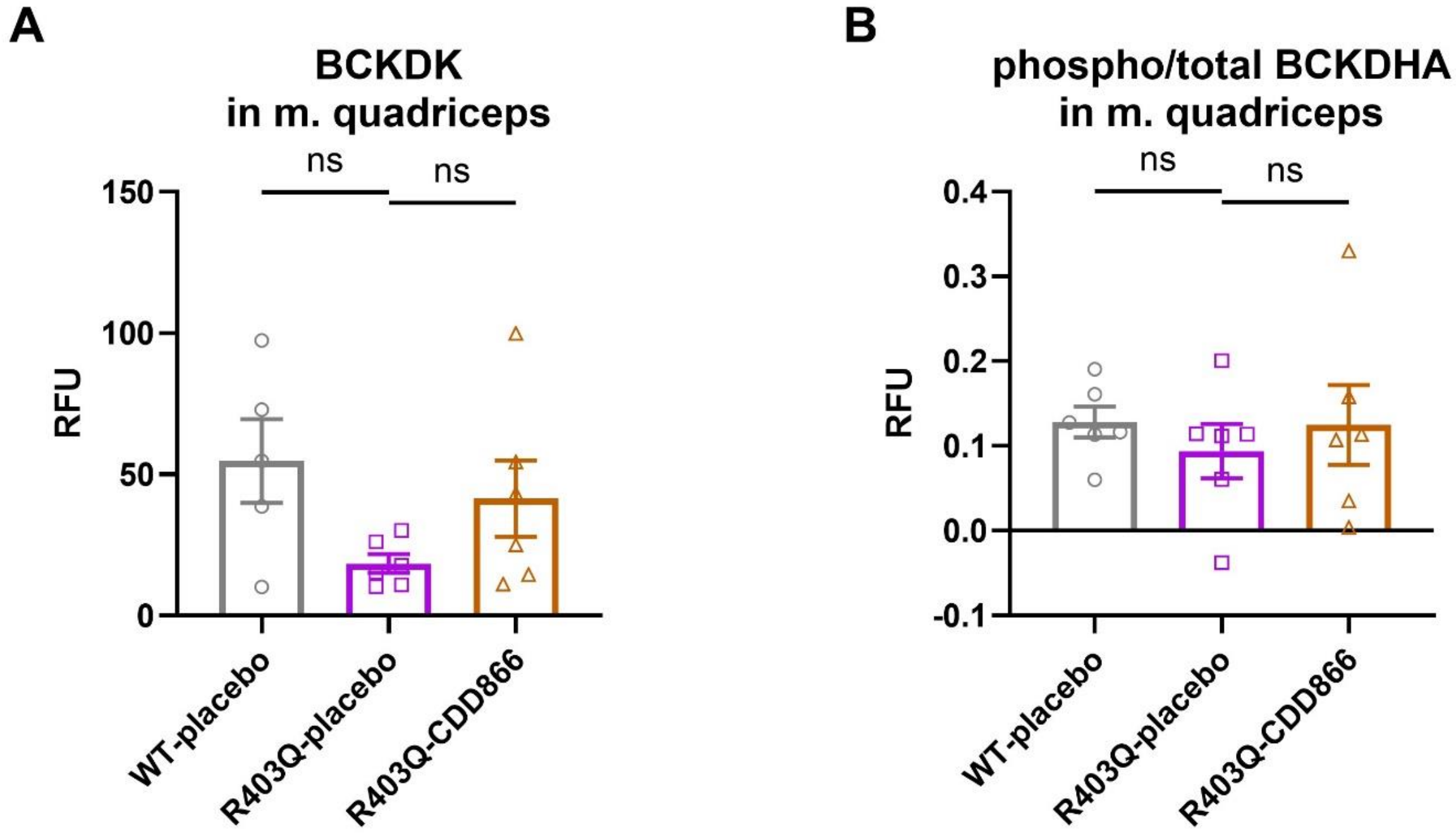


**Figure S8. Western blot analysis of quadriceps muscle.** CDD866 nonsignificantly increased the expression of branched-chain ketoacid dehydrogenase kinase (BCKDK) in R403Q mice (**A**), which was associated with a trend for increased phosphorylation of the branched-chain ketoacid dehydrogenase E1 alpha subunit (BCKDHA) a rate-limiting step in BCAA catabolism, as the ratio of phosphorylated-to-total BCKDHA increased (**B**). Data shown are mean±SEM. n = 5-6. *p*-values were obtained by one-way ANOVA with Bonferroni multiple comparisons tests (adjusted *p*-value threshold <0.05). ns, not significant.

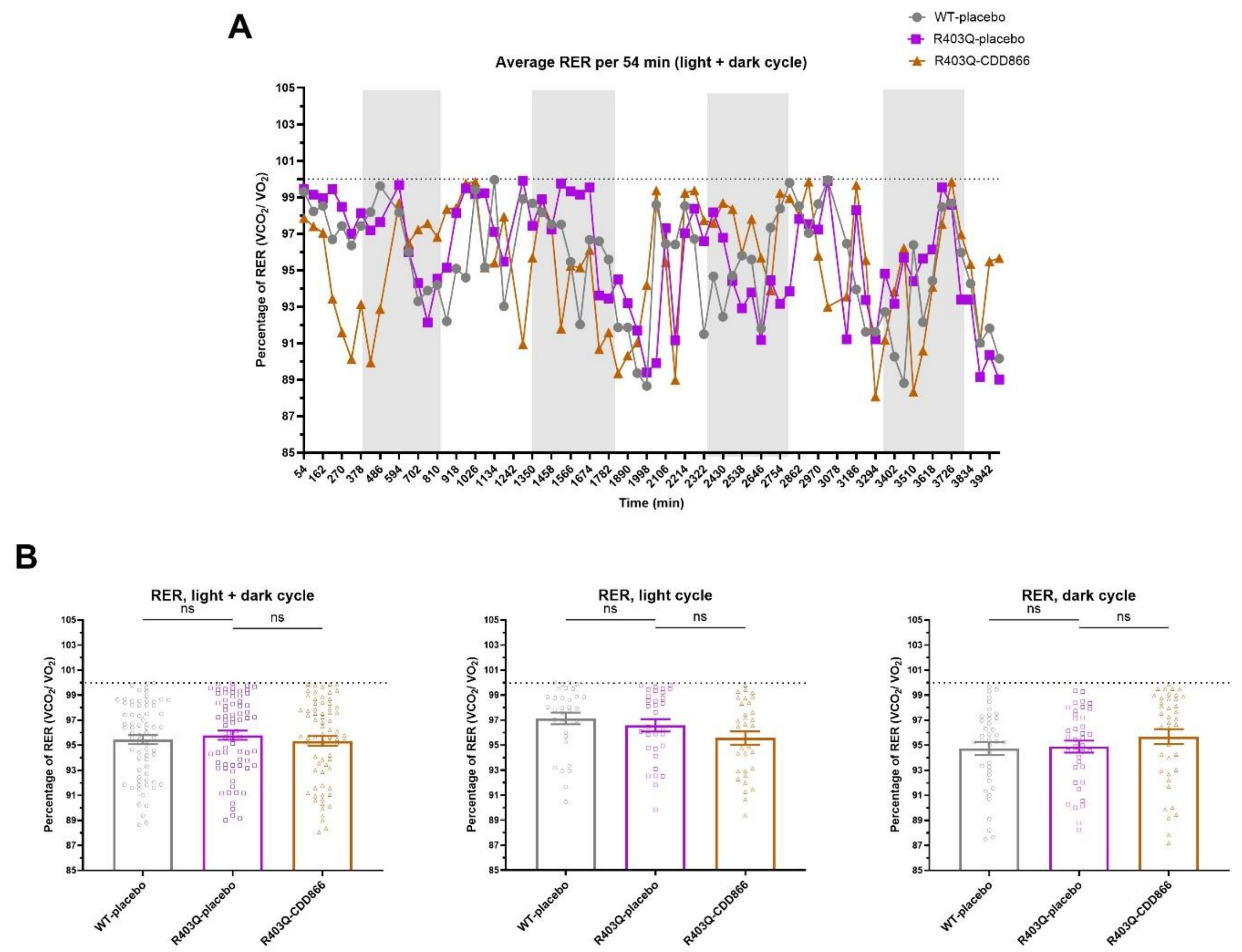


**Figure S9. Respiratory exchange ratio (RER) in metabolic cages.** No profound differences in the respiratory exchange ratio (RER) (**A** and **B**), a proxy for metabolic fuel utilization, flexibility and efficiency, were found amongst study groups during 4 days in Comprehensive Laboratory Animal Monitoring System metabolic cages (CLAMS), while the RER's physiological circadian oscillation was maintained (**A**). The RER data at each 54-minute mark were averaged across all animals within the group. Percent differences were calculated using the averaged RER of the full 4 days in WT-placebo as the control. The absolute value of each resulting data point was taken, and subtracted from 100% to represent the percentage of RER compared to the WT-placebo. Data shown are mean±SEM. n = 4. *p*-values were obtained by 2-way ANOVA with Bonferroni multiple comparisons tests (adjusted *p*-value threshold <0.05). ns, not significant.

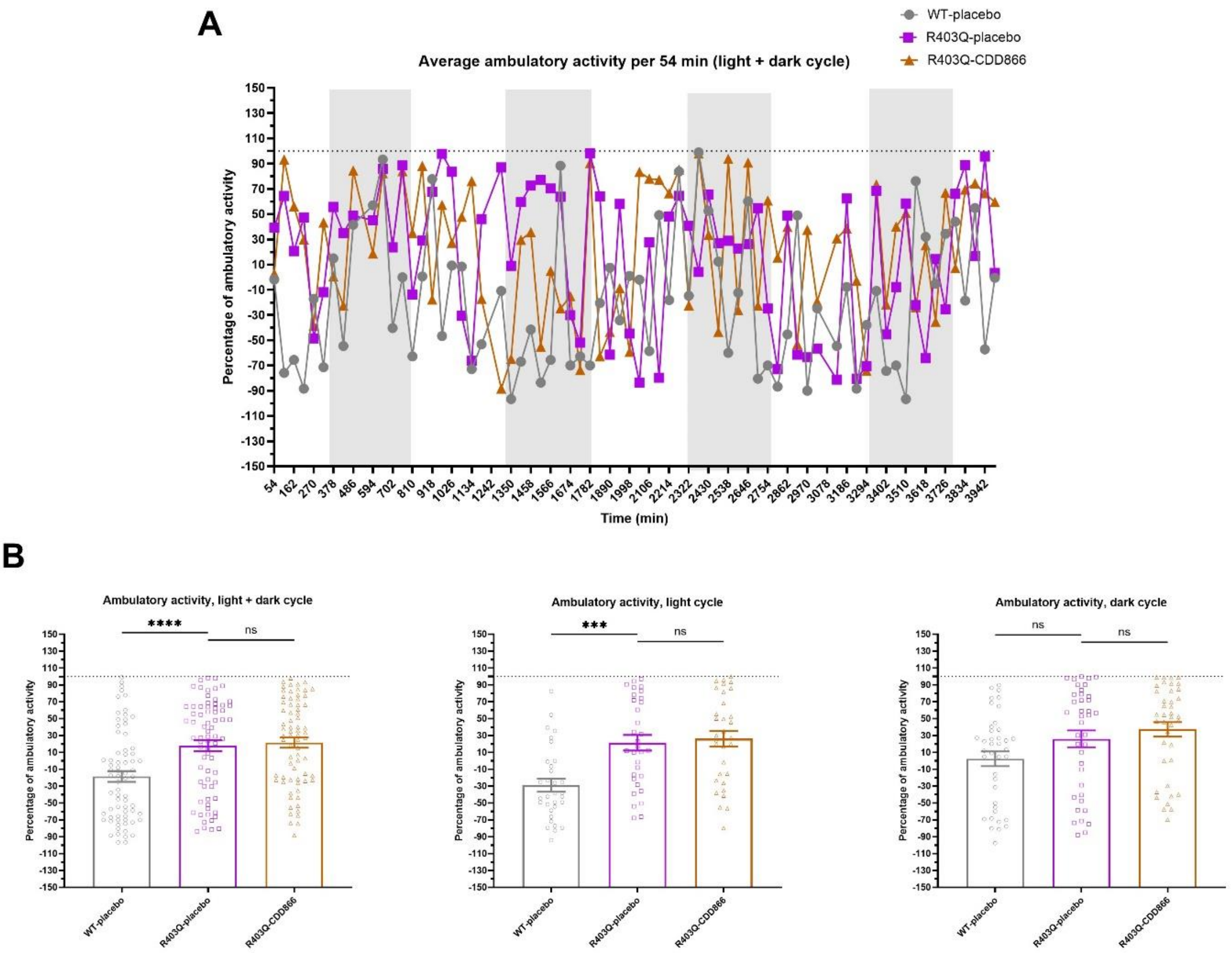


**Figure S10. Ambulatory activity in metabolic cages.** CDD866 did not affect the ambulatory activity of R403Q mice during 4 days in Comprehensive Laboratory Animal Monitoring System metabolic cages (CLAMS) (**A** and **B**), while maintaining its circadian oscillation (**A**). This evidence shows that CDD866-induced skeletal muscle hypertrophy was not accompanied by a detected increase in ambulatory activity. The ambulatory activity data at each 54-minute mark were averaged across all animals within the group. Percent differences were calculated using the averaged ambulatory activity of the full 4 days in WT-placebo as the control. The absolute value of each resulting data point was taken, and subtracted from 100% to represent the percentage of ambulatory activity compared to the WT-placebo. Data shown are mean±SEM. n = 4. *p*-values were obtained by 2-way ANOVA with Bonferroni multiple comparisons tests (adjusted *p*-value threshold <0.05). ***$p<0.001$; ****$p<0.0001$; ns, not significant.

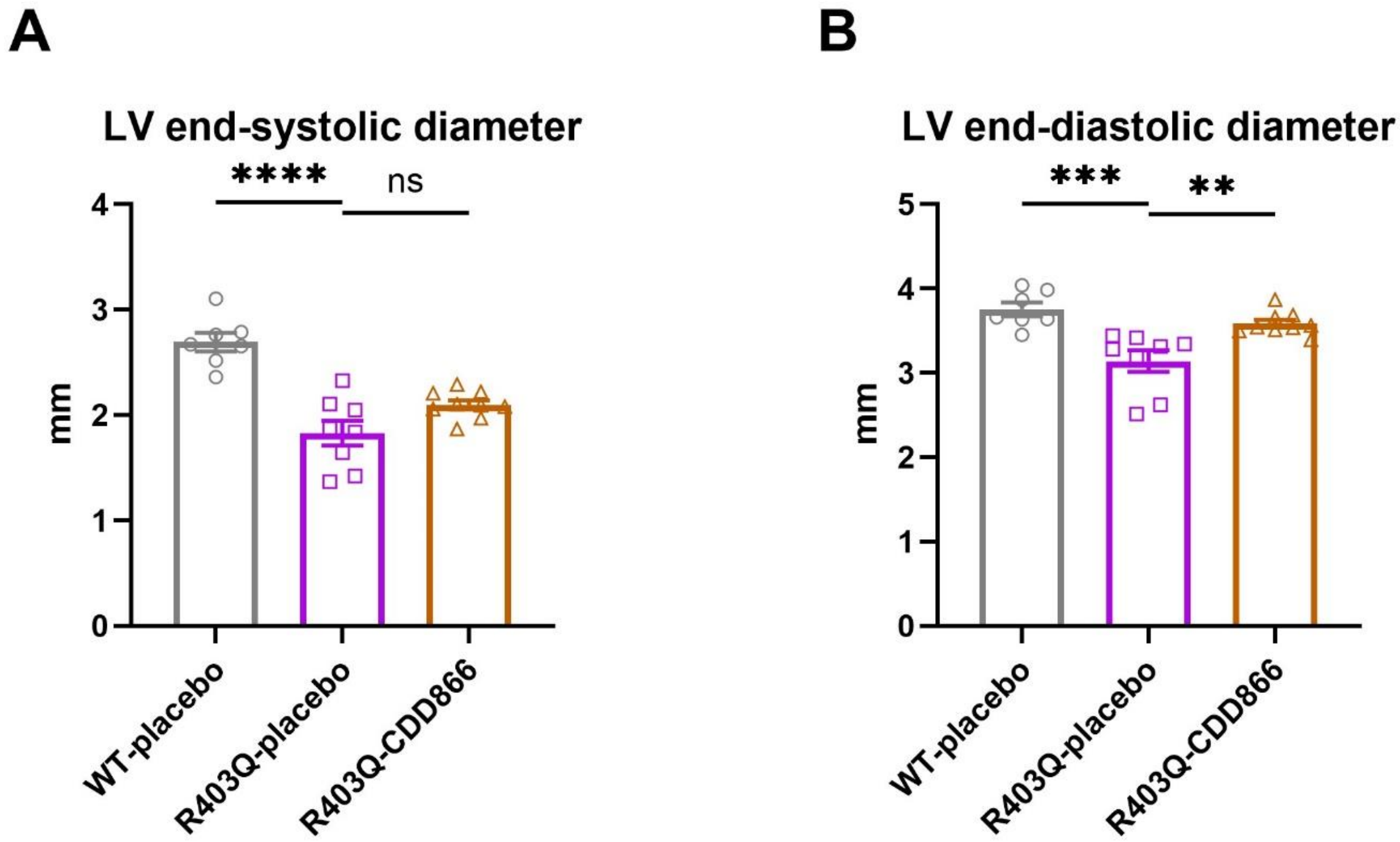


**Figure S11. Heart dimensions after 4-week CDD866 treatment as assessed by echocardiography.** R403Q hearts showed decreased left ventricular end-systolic diameter (ESD) (**A**) and end-diastolic diameter (EDD) (**B**). 4-week CDD866 treatment improved EDD with a trend for ESD improvement. Data shown are mean±SEM. n = 7 (WT-placebo), 8 (R403Q-placebo), and 9 (R403Q-CDD866). *p*-values were obtained by one-way ANOVA with Bonferroni multiple comparisons tests (adjusted *p*-value threshold $<0.05$). **$p<0.01$; ***$p<0.001$; ****$p<0.0001$; ns, not significant.

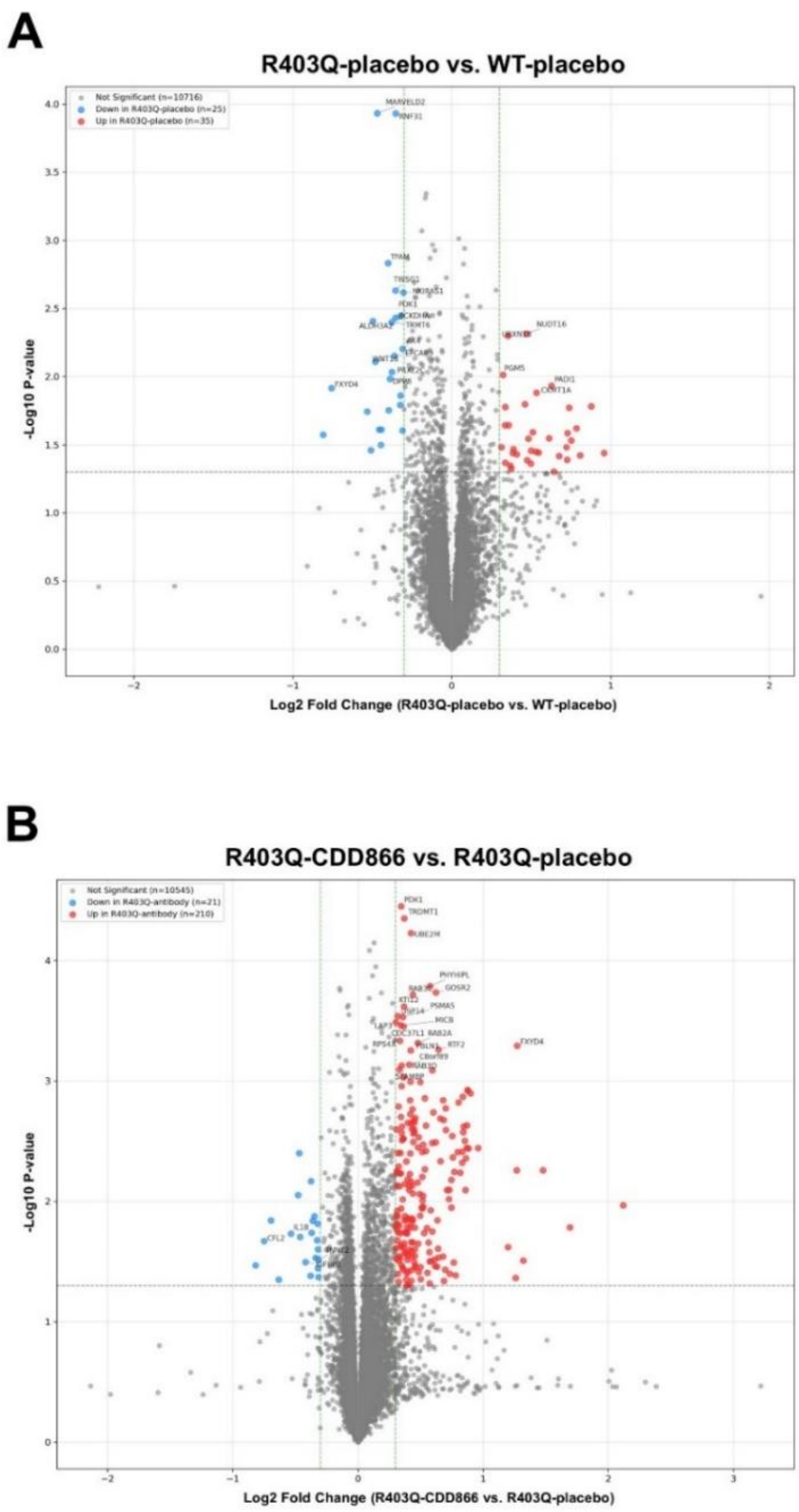


**Figure S12. Cardiac proteomics volcano plots.** Hearts of 28-week-old animals after 4 weeks of placebo (**A**) or CDD866 (**B**) treatment were used (n = 5-6 animals per group). Proteins with significant differential abundance between R403Q-placebo and WT-placebo or between R403Q-CDD866 and R403Q-placebo were identified using two-sided independent-samples Student's t-tests ($p<0.05$) for each protein. Volcano plots were generated for each comparison, displaying log2 fold change on the x-axis and −log10 raw p-value on the y-axis. Proteins meeting both significance thresholds ($p < 0.05$ and $|\log2 \text{FC}| > 0.3$) were highlighted, with upregulated proteins shown in red and downregulated proteins in blue. mean FC, mean fold change; p-value, nominal *p*-value. $p<0.05$ was considered significant. Highlighted proteins are based on nominal p-values, not FDR-significant proteins.

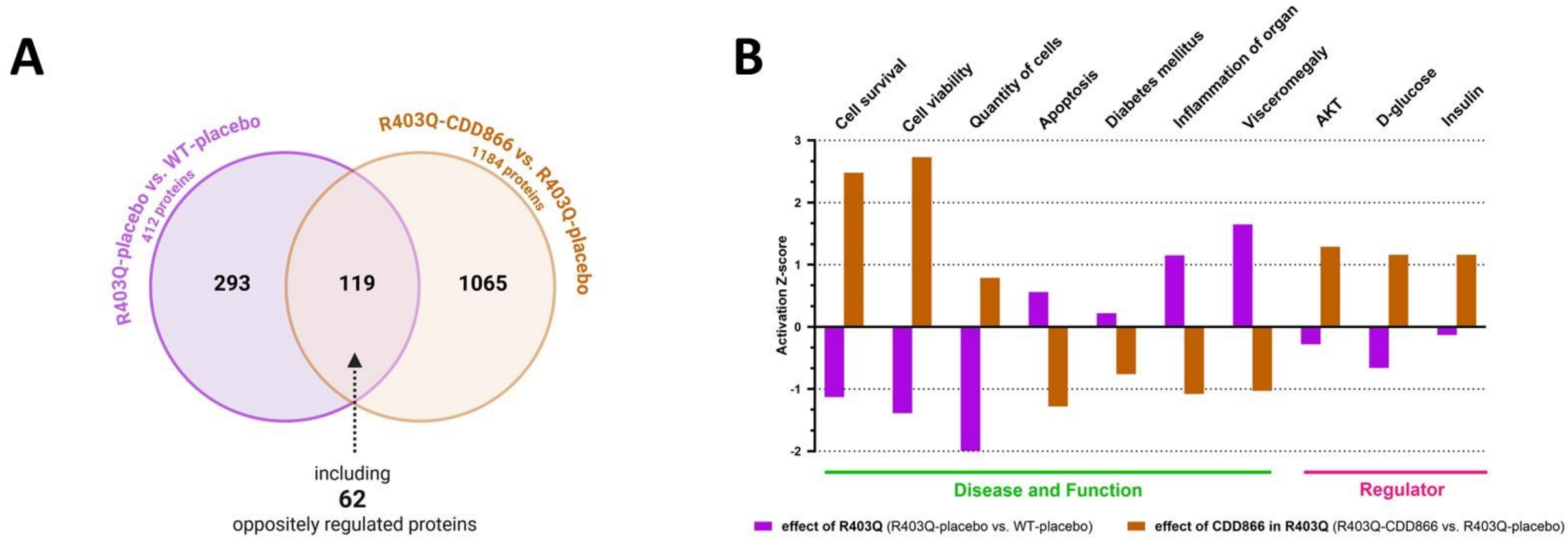


**Figure S13. Cardiac proteomics.** (A) At nominal $p < 0.05$, 412 proteins differed between R403Q-placebo and WT-placebo hearts and 1,184 differed between R403Q-CDD866 and R403Q-placebo hearts; 119 met this threshold in both comparisons, including 62 with opposite directions of change (Tables S5 and S6). (B) Selected IPA disease/function annotations and upstream-regulator predictions for the disease and treatment comparisons. Bars show activation z-scores; positive and negative scores indicate predicted directions, with most scores having $|z| < 2$. The selected entries met nominal overlap $p < 0.05$. Opposite signs suggest potential counter-regulation and do not demonstrate restored insulin/AKT signaling or reversal of a diabetic phenotype. IPA inputs used nominal protein-level $p < 0.05$ for disease and $p < 0.01$ for treatment. No individual proteins survived BH FDR correction, and IPA overlap p-values were unadjusted; these analyses are exploratory.

## Supplementary Tables

**Table S1. Selected cardiac metabolic pathways depicted in Figure 3B.** Hearts of 28-week-old animals after 4 weeks of placebo or CDD866 treatment were used (n = 5 animals per group). Differential expression was assessed using the Wald test implemented in the DESeq2 R package. Gene sets were obtained from the Molecular Signatures Database (MSigDB), version 2024.1.Mm. FDR *q*, Benjamini-Hochberg false discovery rate-corrected *p*-value. NES, Normalized Enrichment Score. FDR $q < 0.05$ was considered significant. Gene sets coordinately upregulated are depicted in red; gene sets coordinately downregulated are depicted in blue.

| Selected cardiac metabolic pathways | | | R403Q-placebo vs WT-placebo | | R403Q-CDD866 vs R403Q-placebo | |
|---|---|---|---|---|---|---|
| **Group** | **Gene Set Name** | **Size** | **NES** | **FDR *q*** | **NES** | **FDR *q*** |
| WikiPathways | FATTY ACID BETAOXIDATION | 33 | -2.37 | ***<0.0001*** | 1.85 | ***0.0325*** |
| Reactome | BRANCHED-CHAIN AMINO ACID CATABOLISM | 20 | -2.49 | ***<0.0001*** | 2.34 | ***0.0003*** |
| Reactome | PYRUVATE METABOLISM | 42 | -2.35 | ***<0.0001*** | 2.28 | ***0.0002*** |
| Reactome | CITRIC ACID CYCLE (TCA CYCLE) | 33 | -2.45 | ***<0.0001*** | 2.13 | ***0.0034*** |
| Hallmark | OXIDATIVE PHOSPHORYLATION | 193 | -2.86 | ***<0.0001*** | 2.47 | ***<0.0001*** |
| GOBP | PROTON MOTIVE FORCE DRIVEN ATP SYNTHESIS | 58 | -2.10 | ***0.0014*** | 1.82 | ***0.0427*** |

**Table S2. Selected gene sets oppositely regulated by CDD866 in R403Q hearts.** Hearts of 28-week-old animals after 4 weeks of placebo or CDD866 treatment were used (n = 5 animals per group). Differential expression was assessed using the Wald test implemented in the DESeq2 R package. Gene sets were obtained from the Molecular Signatures Database (MSigDB), version 2024.1.Mm. FDR *q*, Benjamini-Hochberg false discovery rate-corrected p-value. NES, Normalized Enrichment Score. *p*, nominal *p*-value. FDR *q* <0.05 was considered significant. Gene sets coordinately upregulated are depicted in red; gene sets coordinately downregulated are depicted in blue.

| Selected pathways by keywords | | | | R403Q-placebo vs WT-placebo | | | R403Q-CDD866 vs R403Q-placebo | | |
|---|---|---|---|---|---|---|---|---|---|
| **Keyword** | **Group** | **Gene Set Name** | **Size** | **NES** | ***p*** | **FDR *q*** | **NES** | ***p*** | **FDR *q*** |
| **Fatty acid** | M2 WikiPathways pathway | FATTY ACID BETAOXIDATION | 33 | -2.37 | <0.0001 | <0.0001 | 1.85 | <0.0001 | 0.0325 |
| | M5 GO Biological Process | FATTY ACID BETA OXIDATION | 75 | -2.21 | <0.0001 | 0.0002 | 1.44 | 0.0314 | 0.2331 |
| | M5 GO Biological Process | FATTY ACID CATABOLIC PROCESS | 101 | -2.06 | <0.0001 | 0.0023 | 1.36 | 0.0477 | 0.2989 |
| | MH Hallmark | FATTY ACID METABOLISM | 149 | -1.99 | <0.0001 | 0.0061 | 1.63 | <0.0001 | 0.1148 |
| | M2 Reactome pathway | FATTY ACID METABOLISM | 153 | -1.97 | <0.0001 | 0.0071 | 1.79 | <0.0001 | 0.0470 |
| | M5 GO Biological Process | FATTY ACID DERIVATIVE METABOLIC PROCESS | 41 | -1.83 | <0.0001 | 0.0241 | 1.48 | 0.0476 | 0.1944 |
| | M2 WikiPathways pathway | FATTY ACID BIOSYNTHESIS | 22 | -1.62 | 0.0135 | 0.0940 | 1.59 | 0.0219 | 0.1342 |
| | M5 GO Biological Process | VERY LONG CHAIN FATTY ACID METABOLIC PROCESS | 32 | -1.58 | 0.0290 | 0.1205 | 1.72 | 0.0068 | 0.0733 |
| | M5 GO Biological Process | FATTY ACID METABOLIC PROCESS | 389 | -1.55 | <0.0001 | 0.1358 | 1.48 | 0.0012 | 0.1942 |
| | M5 GO Biological Process | FATTY ACID BIOSYNTHETIC PROCESS | 145 | -1.42 | 0.0108 | 0.2329 | 1.51 | 0.0088 | 0.1770 |
| **Glucose** | M5 GO Biological Process | GLUCOSE METABOLIC PROCESS | 206 | -1.92 | <0.0001 | 0.0111 | 1.29 | 0.0504 | 0.3682 |
| | M5 GO Biological Process | POSITIVE REGULATION OF GLUCOSE IMPORT | 40 | -1.42 | 0.0481 | 0.2284 | 1.52 | 0.0271 | 0.1736 |
| **Pyruvate** | M2 Reactome pathway | PYRUVATE METABOLISM | 42 | -2.35 | <0.0001 | <0.0001 | 2.28 | <0.0001 | 0.0002 |
| | M2 Reactome pathway | REGULATION OF PYRUVATE METABOLISM | 32 | -2.30 | <0.0001 | <0.0001 | 2.12 | <0.0001 | 0.0042 |
| | M5 GO Biological Process | PYRUVATE METABOLIC PROCESS | 129 | -2.00 | <0.0001 | 0.0051 | 1.35 | 0.0339 | 0.2990 |
| **TCA** | M2 Reactome pathway | CITRIC ACID CYCLE TCA CYCLE | 33 | -2.45 | <0.0001 | <0.0001 | 2.13 | <0.0001 | 0.0034 |
| | M2 WikiPathways pathway | TCA CYCLE | 30 | -2.50 | <0.0001 | <0.0001 | 2.21 | <0.0001 | 0.0006 |
| | M2 Reactome pathway | MATURATION OF TCA ENZYMES AND REGULATION OF TCA CYCLE | 18 | -1.99 | 0.0019 | 0.0060 | 1.85 | <0.0001 | 0.0330 |
| | M5 GO Biological Process | TRICARBOXYLIC ACID CYCLE | 34 | -2.29 | <0.0001 | <0.0001 | 1.95 | <0.0001 | 0.0218 |
| | M5 GO Cellular Component | TRICARBOXYLIC ACID CYCLE HETEROMERIC ENZYME COMPLEX | 20 | -2.21 | <0.0001 | 0.0002 | 1.89 | 0.0018 | 0.0278 |
| **Oxidative phosphorylation** | MH Hallmark | OXIDATIVE PHOSPHORYLATION | 193 | -2.86 | <0.0001 | <0.0001 | 2.47 | <0.0001 | <0.0001 |
| | M5 GO Biological Process | OXIDATIVE PHOSPHORYLATION | 126 | -2.10 | <0.0001 | 0.0014 | 1.69 | <0.0001 | 0.0896 |
| | M2 WikiPathways pathway | OXIDATIVE PHOSPHORYLATION | 45 | -1.95 | <0.0001 | 0.0087 | 1.91 | <0.0001 | 0.0246 |
| **ATP** | M5 GO Biological Process | ATP BIOSYNTHETIC PROCESS | 85 | -2.14 | <0.0001 | 0.0008 | 1.70 | <0.0001 | 0.0840 |
| | M5 GO Biological Process | ATP SYNTHESIS COUPLED ELECTRON TRANSPORT | 66 | -2.13 | <0.0001 | 0.0009 | 1.74 | 0.0032 | 0.0696 |
| | M5 GO Biological Process | ATP METABOLIC PROCESS | 214 | -2.12 | <0.0001 | 0.0010 | 1.33 | 0.0309 | 0.3225 |
| | M5 GO Biological Process | PROTON MOTIVE FORCE DRIVEN ATP SYNTHESIS | 58 | -2.10 | <0.0001 | 0.0014 | 1.82 | <0.0001 | 0.0427 |
| | M5 GO Cellular Component | PROTON TRANSPORTING TWO SECTOR ATPASE COMPLEX CATALYTIC DOMAIN | 17 | -1.75 | 0.0038 | 0.0424 | 1.85 | 0.0073 | 0.0327 |
| | M5 GO Molecular Function | ATPASE REGULATOR ACTIVITY | 47 | -1.67 | 0.0038 | 0.0753 | 1.59 | 0.0083 | 0.1352 |
| | M2 Reactome pathway | FORMATION OF ATP BY CHEMIOSMOTIC COUPLING | 18 | -1.65 | 0.0095 | 0.0834 | 1.84 | 0.0034 | 0.0343 |
| | M5 GO Cellular Component | PROTON TRANSPORTING ATP SYNTHASE COMPLEX | 18 | -1.64 | 0.0096 | 0.0861 | 1.80 | <0.0001 | 0.0450 |
| | M5 GO Molecular Function | ATPASE ACTIVATOR ACTIVITY | 27 | -1.54 | 0.0294 | 0.1417 | 1.43 | 0.0592 | 0.2385 |
| | M5 GO Cellular Component | PROTON TRANSPORTING TWO SECTOR ATPASE COMPLEX | 46 | -1.48 | 0.0285 | 0.1883 | 1.66 | 0.0083 | 0.1023 |
| **Cardiac** | M5 GO Biological Process | REGULATION OF CARDIAC MUSCLE CELL ACTION POTENTIAL | 27 | -1.91 | <0.0001 | 0.0127 | 1.49 | 0.0389 | 0.1902 |
| | M5 GO Biological Process | CARDIAC EPITHELIAL TO MESENCHYMAL TRANSITION | 35 | 1.92 | <0.0001 | 0.0174 | -1.57 | 0.0102 | 0.2610 |
| | M5 GO Biological Process | CARDIAC CELL DEVELOPMENT | 120 | -1.78 | <0.0001 | 0.0361 | 1.37 | 0.0400 | 0.2894 |
| **Collagen** | M5 GO Cellular Component | COLLAGEN CONTAINING EXTRACELLULAR MATRIX | 362 | 2.86 | <0.0001 | <0.0001 | -2.01 | <0.0001 | 0.0375 |
| | M5 GO Molecular Function | COLLAGEN BINDING | 70 | 2.40 | <0.0001 | <0.0001 | -1.59 | 0.0055 | 0.2466 |
| | M2 Reactome pathway | COLLAGEN CHAIN TRIMERIZATION | 41 | 2.48 | <0.0001 | <0.0001 | -1.65 | 0.0080 | 0.2152 |
| | M2 Reactome pathway | COLLAGEN FORMATION | 73 | 2.25 | <0.0001 | <0.0001 | -1.75 | <0.0001 | 0.1640 |
| | M2 Reactome pathway | COLLAGEN BIOSYNTHESIS AND MODIFYING ENZYMES | 61 | 2.32 | <0.0001 | 0.0001 | -1.93 | <0.0001 | 0.0827 |
| | M5 GO Biological Process | COLLAGEN METABOLIC PROCESS | 117 | 1.95 | <0.0001 | 0.0141 | -1.47 | <0.0001 | 0.3163 |

## Table S2. Continued.

| Selected pathways by keywords | | | | R403Q-placebo vs WT-placebo | | | R403Q-CDD866 vs R403Q-placebo | | |
|---|---|---|---|---|---|---|---|---|---|
| Keyword | Group | Gene Set Name | Size | NES | *p* | FDR *q* | NES | *p* | FDR *q* |
| Insulin | M2 BioCarta pathway | INSULIN PATHWAY | 22 | -1.48 | 0.0370 | 0.1823 | 1.76 | 0.0109 | 0.0602 |
| | M5 GO Biological Process | POSITIVE REGULATION OF CELLULAR RESPONSE TO INSULIN STIMULUS | 35 | -1.44 | 0.0422 | 0.2172 | 1.73 | 0.0034 | 0.0732 |
| | M5 GO Biological Process | CELLULAR RESPONSE TO INSULIN STIMULUS | 212 | -1.43 | 0.0036 | 0.2204 | 1.63 | 0.0013 | 0.1127 |
| | M5 GO Biological Process | RESPONSE TO INSULIN | 252 | -1.38 | 0.0087 | 0.2620 | 1.54 | 0.0013 | 0.1596 |
| | M2 WikiPathways pathway | INSULIN SIGNALING | 158 | -1.37 | 0.0127 | 0.2694 | 1.42 | 0.0185 | 0.2461 |
| | M5 GO Biological Process | REGULATION OF CELLULAR RESPONSE TO INSULIN STIMULUS | 88 | -1.28 | 0.0824 | 0.3453 | 1.55 | 0.0062 | 0.1565 |
| | M5 GO Biological Process | INSULIN RECEPTOR SIGNALING PATHWAY | 135 | -1.28 | 0.0655 | 0.3464 | 1.60 | 0.0043 | 0.1262 |
| BCAA | M5 GO Biological Process | BRANCHED-CHAIN AMINO ACID CATABOLIC PROCESS | 21 | -2.47 | <0.0001 | <0.0001 | 2.25 | <0.0001 | 0.0003 |
| | M5 GO Biological Process | BRANCHED-CHAIN AMINO ACID METABOLIC PROCESS | 25 | -2.47 | <0.0001 | <0.0001 | 2.20 | <0.0001 | 0.0008 |
| | M2 Reactome pathway | BRANCHED-CHAIN AMINO ACID CATABOLISM | 20 | -2.49 | <0.0001 | <0.0001 | 2.34 | <0.0001 | 0.0003 |
| Mitochondrial | M5 GO Biological Process | MITOCHONDRIAL GENE EXPRESSION | 165 | -2.44 | <0.0001 | <0.0001 | 1.96 | <0.0001 | 0.0208 |
| | M5 GO Biological Process | MITOCHONDRIAL TRANSLATION | 128 | -2.41 | <0.0001 | <0.0001 | 1.81 | <0.0001 | 0.0439 |
| | M5 GO Cellular Component | MITOCHONDRIAL MATRIX | 289 | -2.78 | <0.0001 | <0.0001 | 2.09 | <0.0001 | 0.0057 |
| | M5 GO Cellular Component | MITOCHONDRIAL PROTEIN CONTAINING COMPLEX | 259 | -2.57 | <0.0001 | <0.0001 | 2.24 | <0.0001 | 0.0005 |
| | M2 Reactome pathway | MITOCHONDRIAL PROTEIN DEGRADATION | 78 | -2.79 | <0.0001 | <0.0001 | 2.33 | <0.0001 | 0.0002 |
| | M2 Reactome pathway | MITOCHONDRIAL TRANSLATION | 88 | -2.30 | <0.0001 | <0.0001 | 1.83 | <0.0001 | 0.0375 |
| | M5 GO Cellular Component | INNER MITOCHONDRIAL MEMBRANE PROTEIN COMPLEX | 131 | -2.20 | <0.0001 | 0.0002 | 1.97 | <0.0001 | 0.0203 |
| | M5 GO Biological Process | INNER MITOCHONDRIAL MEMBRANE ORGANIZATION | 38 | -2.15 | <0.0001 | 0.0006 | 1.97 | 0.0016 | 0.0202 |
| | M5 GO Biological Process | MITOCHONDRIAL RNA METABOLIC PROCESS | 53 | -2.10 | <0.0001 | 0.0014 | 1.96 | 0.0016 | 0.0208 |
| | M5 GO Cellular Component | MITOCHONDRIAL LARGE RIBOSOMAL SUBUNIT | 55 | -2.09 | <0.0001 | 0.0014 | 1.63 | 0.0081 | 0.1151 |
| | M5 GO Biological Process | MITOCHONDRIAL TRANSMEMBRANE TRANSPORT | 91 | -2.08 | <0.0001 | 0.0018 | 1.92 | <0.0001 | 0.0235 |
| | M5 GO Biological Process | MITOCHONDRIAL TRANSPORT | 175 | -1.95 | <0.0001 | 0.0088 | 1.77 | <0.0001 | 0.0555 |
| | M5 GO Cellular Component | MITOCHONDRIAL SMALL RIBOSOMAL SUBUNIT | 32 | -1.93 | <0.0001 | 0.0104 | 1.93 | <0.0001 | 0.0243 |
| | M5 GO Biological Process | MITOCHONDRIAL FISSION | 49 | -1.91 | <0.0001 | 0.0128 | 2.15 | <0.0001 | 0.0031 |
| | M5 GO Biological Process | MITOCHONDRIAL ELECTRON TRANSPORT NADH TO UBIQUINONE | 23 | -1.87 | 0.0019 | 0.0171 | 1.65 | 0.0123 | 0.1053 |
| | M5 GO Biological Process | MITOCHONDRIAL RESPIRATORY CHAIN COMPLEX ASSEMBLY | 91 | -1.87 | <0.0001 | 0.0172 | 1.81 | <0.0001 | 0.0450 |
| | M5 GO Biological Process | MITOCHONDRIAL CALCIUM ION TRANSMEMBRANE TRANSPORT | 19 | -1.88 | <0.0001 | 0.0173 | 1.50 | 0.0480 | 0.1879 |
| | M5 GO Biological Process | REGULATION OF AUTOPHAGY OF MITOCHONDRION | 31 | -1.86 | <0.0001 | 0.0202 | 1.45 | 0.0493 | 0.2203 |
| | M2 Reactome pathway | MITOCHONDRIAL BIOGENESIS | 28 | -1.86 | <0.0001 | 0.0204 | 1.88 | <0.0001 | 0.0290 |
| | M5 GO Biological Process | IMPORT INTO THE MITOCHONDRION | 48 | -1.84 | <0.0001 | 0.0230 | 1.48 | 0.0268 | 0.1952 |
| | M5 GO Biological Process | MITOCHONDRIAL MEMBRANE ORGANIZATION | 110 | -1.83 | <0.0001 | 0.0249 | 1.61 | 0.0044 | 0.1221 |
| | M5 GO Biological Process | MITOCHONDRIAL RNA PROCESSING | 20 | -1.82 | <0.0001 | 0.0255 | 1.75 | 0.0070 | 0.0653 |
| | M5 GO Biological Process | MITOCHONDRIAL FUSION | 35 | -1.80 | 0.0020 | 0.0291 | 1.50 | 0.0340 | 0.1821 |
| | M5 GO Biological Process | AUTOPHAGY OF MITOCHONDRION | 92 | -1.80 | <0.0001 | 0.0302 | 1.37 | 0.0462 | 0.2832 |
| | M5 GO Biological Process | PROTEIN LOCALIZATION TO MITOCHONDRION | 102 | -1.80 | <0.0001 | 0.0304 | 1.66 | <0.0001 | 0.1021 |
| | M2 Reactome pathway | MITOCHONDRIAL CALCIUM ION TRANSPORT | 17 | -1.75 | 0.0037 | 0.0426 | 1.63 | 0.0199 | 0.1124 |
| | M5 GO Biological Process | PROTEIN IMPORT INTO MITOCHONDRIAL MATRIX | 18 | -1.73 | 0.0081 | 0.0501 | 1.52 | 0.0357 | 0.1736 |
| | M5 GO Biological Process | REGULATION OF MITOCHONDRIAL FISSION | 36 | -1.71 | 0.0077 | 0.0584 | 2.02 | <0.0001 | 0.0114 |
| | M5 GO Biological Process | PROTEIN TARGETING TO MITOCHONDRION | 76 | -1.65 | 0.0019 | 0.0825 | 1.64 | 0.0091 | 0.1104 |
| | M5 GO Biological Process | POSITIVE REGULATION OF MITOCHONDRIAL FISSION | 23 | -1.62 | 0.0257 | 0.0952 | 1.81 | 0.0017 | 0.0442 |
| | M5 GO Biological Process | MITOCHONDRIAL TRANSCRIPTION | 22 | -1.58 | 0.0222 | 0.1205 | 1.51 | 0.0462 | 0.1807 |
| | M5 GO Cellular Component | MITOCHONDRIA ASSOCIATED ENDOPLASMIC RETICULUM MEMBRANE CONTACT SITE | 20 | -1.55 | 0.0292 | 0.1377 | 1.60 | 0.0153 | 0.1244 |
| | M5 GO Biological Process | REGULATION OF MITOCHONDRION ORGANIZATION | 139 | -1.45 | 0.0054 | 0.2082 | 1.30 | 0.0399 | 0.3548 |
| | M5 GO Cellular Component | MITOCHONDRIAL CRISTA | 16 | -1.45 | 0.0806 | 0.2095 | 1.60 | 0.0279 | 0.1292 |

**Table S3. Top 20 most up- or down-regulated gene sets in R403Q hearts and their normalization by CDD866.** Hearts of 28-week-old animals after 4 weeks of placebo or CDD866 treatment were used (n = 5 animals per group). Differential expression was assessed using the Wald test implemented in the DESeq2 R package. Gene sets were obtained from the Molecular Signatures Database (MSigDB), version 2024.1.Mm. FDR *q*, Benjamini-Hochberg false discovery rate-corrected *p*-value. NES, Normalized Enrichment Score. *p*, nominal *p*-value. FDR *q* < 0.05 was considered significant. Gene sets coordinately upregulated are depicted in red; gene sets coordinately downregulated are depicted in blue.

| 20 most DOWN-regulated pathways (by NES) in R403Q-placebo vs WT-placebo | | | R403Q-placebo vs WT-placebo | | | R403Q-CDD866 vs R403Q-placebo | | |
|---|---|---|---|---|---|---|---|---|
| Group | Gene Set Name | Size | NES | *p* | FDR *q* | NES | *p* | FDR *q* |
| MH Hallmark | OXIDATIVE PHOSPHORYLATION | 193 | -2.86 | <0.0001 | <0.0001 | 2.47 | <0.0001 | <0.0001 |
| M2 Reactome pathway | MITOCHONDRIAL PROTEIN DEGRADATION | 78 | -2.79 | <0.0001 | <0.0001 | 2.33 | <0.0001 | 0.0002 |
| M5 GO Cellular Component | MITOCHONDRIAL MATRIX | 289 | -2.78 | <0.0001 | <0.0001 | 2.09 | <0.0001 | 0.0057 |
| M2 Reactome pathway | AEROBIC RESPIRATION AND RESPIRATORY ELECTRON TRANSPORT | 218 | -2.77 | <0.0001 | <0.0001 | 2.37 | <0.0001 | 0.0000 |
| M5 GO Cellular Component | MITOCHONDRIAL PROTEIN CONTAINING COMPLEX | 259 | -2.57 | <0.0001 | <0.0001 | 2.24 | <0.0001 | 0.0005 |
| M2 WikiPathways pathway | TCA CYCLE | 30 | -2.50 | <0.0001 | <0.0001 | 2.21 | <0.0001 | 0.0006 |
| M2 Reactome pathway | BRANCHED-CHAIN AMINO ACID CATABOLISM | 20 | -2.49 | <0.0001 | <0.0001 | 2.34 | <0.0001 | 0.0003 |
| M5 GO Biological Process | BRANCHED-CHAIN AMINO ACID METABOLIC PROCESS | 25 | -2.47 | <0.0001 | <0.0001 | 2.20 | <0.0001 | 0.0008 |
| M5 GO Biological Process | BRANCHED-CHAIN AMINO ACID CATABOLIC PROCESS | 21 | -2.47 | <0.0001 | <0.0001 | 2.25 | <0.0001 | 0.0003 |
| M2 Reactome pathway | CITRIC ACID CYCLE (TCA CYCLE) | 33 | -2.45 | <0.0001 | <0.0001 | 2.13 | <0.0001 | 0.0034 |
| M5 GO Biological Process | MITOCHONDRIAL GENE EXPRESSION | 165 | -2.44 | <0.0001 | <0.0001 | 1.96 | <0.0001 | 0.0208 |
| M5 GO Biological Process | MITOCHONDRIAL TRANSLATION | 128 | -2.41 | <0.0001 | <0.0001 | 1.81 | <0.0001 | 0.0439 |
| M2 WikiPathways pathway | AMINO ACID METABOLISM | 92 | -2.40 | <0.0001 | <0.0001 | 1.96 | <0.0001 | 0.0205 |
| M5 GO Biological Process | CELLULAR RESPIRATION | 221 | -2.39 | <0.0001 | <0.0001 | 1.81 | <0.0001 | 0.0453 |
| M5 GO Biological Process | AEROBIC RESPIRATION | 174 | -2.38 | <0.0001 | <0.0001 | 1.81 | <0.0001 | 0.0455 |
| M2 WikiPathways pathway | FATTY ACID BETAOXIDATION | 33 | -2.37 | <0.0001 | <0.0001 | 1.85 | <0.0001 | 0.0325 |
| M5 GO Biological Process | AMINO ACID CATABOLIC PROCESS | 88 | -2.37 | <0.0001 | <0.0001 | 1.92 | <0.0001 | 0.0251 |
| M2 Reactome pathway | PYRUVATE METABOLISM | 42 | -2.35 | <0.0001 | <0.0001 | 2.28 | <0.0001 | 0.0002 |
| M2 Reactome pathway | MITOCHONDRIAL FATTY ACID BETA OXIDATION | 36 | -2.35 | <0.0001 | <0.0001 | 1.30 | 0.1119 | 0.3498 |
| M5 GO Cellular Component | OXIDOREDUCTASE COMPLEX | 104 | -2.34 | <0.0001 | <0.0001 | 1.77 | 0.0015 | 0.0557 |

| 20 most UP-regulated pathways (by NES) in R403Q-placebo vs WT-placebo | | | R403Q-placebo vs WT-placebo | | | R403Q-CDD866 vs R403Q-placebo | | |
|---|---|---|---|---|---|---|---|---|
| Group | Gene Set Name | Size | NES | *p* | FDR *q* | NES | *p* | FDR *q* |
| M5 GO Cellular Component | COLLAGEN CONTAINING EXTRACELLULAR MATRIX | 362 | 2.86 | <0.0001 | <0.0001 | -2.01 | <0.0001 | 0.0375 |
| M5 GO Cellular Component | EXTERNAL ENCAPSULATING STRUCTURE | 476 | 2.85 | <0.0001 | <0.0001 | -2.05 | <0.0001 | 0.0214 |
| M5 GO Molecular Function | EXTRACELLULAR MATRIX STRUCTURAL CONSTITUENT | 138 | 2.69 | <0.0001 | <0.0001 | -1.82 | <0.0001 | 0.1048 |
| M2 WikiPathways pathway | CYTOPLASMIC RIBOSOMAL PROTEINS | 76 | 2.69 | <0.0001 | <0.0001 | -2.68 | <0.0001 | <0.0001 |
| MH Hallmark | EPITHELIAL MESENCHYMAL TRANSITION | 192 | 2.66 | <0.0001 | <0.0001 | -1.50 | <0.0001 | 0.3025 |
| M2 Reactome pathway | COLLAGEN CHAIN TRIMERIZATION | 41 | 2.48 | <0.0001 | <0.0001 | -1.65 | 0.0080 | 0.2152 |
| M5 GO Cellular Component | COLLAGEN TRIMER | 77 | 2.44 | <0.0001 | <0.0001 | -1.56 | 0.0060 | 0.2599 |
| M5 GO Biological Process | TRANSLATION AT SYNAPSE | 44 | 2.42 | <0.0001 | <0.0001 | -2.46 | <0.0001 | <0.0001 |
| M2 Reactome pathway | EUKARYOTIC TRANSLATION INITIATION | 111 | 2.41 | <0.0001 | <0.0001 | -2.28 | <0.0001 | 0.0004 |
| M5 GO Molecular Function | COLLAGEN BINDING | 70 | 2.40 | <0.0001 | <0.0001 | -1.59 | 0.0055 | 0.2466 |
| M5 GO Molecular Function | FIBRONECTIN BINDING | 36 | 2.39 | <0.0001 | <0.0001 | -1.33 | 0.0698 | 0.4034 |
| M5 GO Molecular Function | EXTRACELLULAR MATRIX STRUCTURAL CONSTITUENT CONFERRING TENSILE STRENGTH | 44 | 2.38 | <0.0001 | <0.0001 | -1.56 | 0.0144 | 0.2596 |
| M2 Reactome pathway | ASSEMBLY OF COLLAGEN FIBRILS AND OTHER MULTIMERIC STRUCTURES | 47 | 2.36 | <0.0001 | <0.0001 | -1.28 | 0.1080 | 0.4355 |
| M2 Reactome pathway | EXTRACELLULAR MATRIX ORGANIZATION | 237 | 2.36 | <0.0001 | <0.0001 | -1.91 | <0.0001 | 0.0948 |
| M2 Reactome pathway | COLLAGEN DEGRADATION | 46 | 2.35 | <0.0001 | <0.0001 | -1.33 | 0.0698 | 0.4038 |
| M2 Reactome pathway | COLLAGEN BIOSYNTHESIS AND MODIFYING ENZYMES | 61 | 2.32 | <0.0001 | 0.0001 | -1.93 | <0.0001 | 0.0827 |
| M5 GO Biological Process | EXTRACELLULAR STRUCTURE ORGANIZATION | 310 | 2.31 | <0.0001 | 0.0001 | -1.48 | <0.0001 | 0.3089 |
| M5 GO Cellular Component | BASEMENT MEMBRANE | 110 | 2.29 | <0.0001 | 0.0001 | -1.64 | <0.0001 | 0.2150 |
| M5 GO Cellular Component | CYTOSOLIC LARGE RIBOSOMAL SUBUNIT | 52 | 2.28 | <0.0001 | 0.0001 | -2.35 | <0.0001 | <0.0001 |
| M2 Reactome pathway | NONSENSE MEDIATED DECAY NMD INDEPENDENT OF THE EXON JUNCTION COMPLEX EJC | 86 | 2.28 | <0.0001 | 0.0001 | -2.54 | <0.0001 | <0.0001 |

**Table S4. Top 20 most up- or down-regulated gene sets by CDD866 in R403Q hearts.** Hearts of 28-week-old animals after 4 weeks of placebo or CDD866 treatment were used (n = 5 animals per group). Differential expression was assessed using the Wald test implemented in the DESeq2 R package. Gene sets were obtained from the Molecular Signatures Database (MSigDB), version 2024.1.Mm. FDR *q*, Benjamini-Hochberg false discovery rate-corrected p-value. NES, Normalized Enrichment Score. *p*, nominal *p*-value. FDR *q* < 0.05 was considered significant. Gene sets coordinately upregulated are depicted in red; gene sets coordinately downregulated are depicted in blue.

| 20 most DOWN-regulated pathways (by NES) in R403Q-CDD866 vs R403Q-placebo | | | R403Q-placebo vs WT-placebo | | | R403Q-CDD866 vs R403Q-placebo | | |
|---|---|---|---|---|---|---|---|---|
| Group | Gene Set Name | Size | NES | *p* | FDR *q* | NES | *p* | FDR *q* |
| M2 WikiPathways pathway | CYTOPLASMIC RIBOSOMAL PROTEINS | 76 | 2.69 | <0.0001 | *<0.0001* | -2.68 | <0.0001 | *<0.0001* |
| M2 Reactome pathway | NONSENSE MEDIATED DECAY NMD INDEPENDENT OF THE EXON JUNCTION COMPLEX EJC | 86 | 2.28 | <0.0001 | *0.0001* | -2.54 | <0.0001 | *<0.0001* |
| M5 GO Biological Process | TRANSLATION AT SYNAPSE | 44 | 2.42 | <0.0001 | *<0.0001* | -2.46 | <0.0001 | *<0.0001* |
| M5 GO Cellular Component | CYTOSOLIC LARGE RIBOSOMAL SUBUNIT | 52 | 2.28 | <0.0001 | *0.0001* | -2.35 | <0.0001 | *<0.0001* |
| M2 Reactome pathway | NONSENSE MEDIATE DECAY NMD | 105 | 2.03 | <0.0001 | *0.0063* | -2.31 | <0.0001 | *<0.0001* |
| M2 Reactome pathway | EUKARYOTIC TRANSLATION INITIATION | 111 | 2.41 | <0.0001 | *<0.0001* | -2.28 | <0.0001 | *0.0004* |
| M2 Reactome pathway | MAJOR PATHWAY OF RRNA PROCESSING IN THE NUCLEOLUS AND CYTOSOL | 167 | 2.26 | <0.0001 | *<0.0001* | -2.26 | <0.0001 | *0.0005* |
| M5 GO Cellular Component | CYTOSOLIC RIBOSOME | 112 | 2.16 | <0.0001 | *0.0010* | -2.24 | <0.0001 | *0.0006* |
| M5 GO Cellular Component | CYTOSOLIC SMALL RIBOSOMAL SUBUNIT | 42 | 2.21 | <0.0001 | *0.0002* | -2.14 | <0.0001 | *0.0063* |
| M5 GO Biological Process | NEUTROPHIL MEDIATED IMMUNITY | 40 | -0.52 | 0.9945 | *1.0000* | -2.08 | <0.0001 | *0.0172* |
| M2 Reactome pathway | TELOMERE C STRAND LAGGING STRAND SYNTHESIS | 33 | 1.02 | 0.4053 | *0.6533* | -2.07 | <0.0001 | *0.0179* |
| M5 GO Cellular Component | EXTERNAL ENCAPSULATING STRUCTURE | 476 | 2.85 | <0.0001 | *<0.0001* | -2.05 | <0.0001 | *0.0214* |
| M5 GO Cellular Component | COLLAGEN CONTAINING EXTRACELLULAR MATRIX | 362 | 2.86 | <0.0001 | *<0.0001* | -2.01 | <0.0001 | *0.0375* |
| M2 Reactome pathway | POLYMERASE SWITCHING ON THE C STRAND OF THE TELOMERE | 25 | 1.15 | 0.2490 | *0.4918* | -2.01 | <0.0001 | *0.0365* |
| M5 GO Biological Process | RIBOSOMAL SMALL SUBUNIT ASSEMBLY | 18 | 1.48 | 0.0556 | *0.2000* | -1.97 | <0.0001 | *0.0618* |
| M2 Reactome pathway | DNA STRAND ELONGATION | 24 | 0.95 | 0.5560 | *0.7517* | -1.97 | 0.0023 | *0.0591* |
| M2 Reactome pathway | ACTIVATION OF THE MRNA UPON BINDING OF THE CAP BINDING COMPLEX AND EIFS AND SUBSEQUENT BINDING TO 43S | 59 | 2.26 | <0.0001 | *0.0001* | -1.95 | <0.0001 | *0.0650* |
| M2 Reactome pathway | COLLAGEN BIOSYNTHESIS AND MODIFYING ENZYMES | 61 | 2.32 | <0.0001 | *0.0001* | -1.93 | <0.0001 | *0.0827* |
| M2 Reactome pathway | HEME DEGRADATION | 16 | 1.74 | 0.0079 | *0.0691* | -1.92 | <0.0001 | *0.0900* |
| M2 WikiPathways pathway | TYROBP CAUSAL NETWORK IN MICROGLIA | 57 | 1.30 | 0.0970 | *0.3325* | -1.91 | <0.0001 | *0.0909* |

| 20 most UP-regulated pathways (by NES) in R403Q-CDD866 vs R403Q-placebo | | | R403Q-placebo vs WT-placebo | | | R403Q-CDD866 vs R403Q-placebo | | |
|---|---|---|---|---|---|---|---|---|
| Group | Gene Set Name | Size | NES | *p* | FDR *q* | NES | *p* | FDR *q* |
| MH Hallmark | OXIDATIVE PHOSPHORYLATION | 193 | -2.86 | <0.0001 | *<0.0001* | 2.47 | <0.0001 | *<0.0001* |
| M2 Reactome pathway | AEROBIC RESPIRATION AND RESPIRATORY ELECTRON TRANSPORT | 218 | -2.77 | <0.0001 | *<0.0001* | 2.37 | <0.0001 | *<0.0001* |
| M2 Reactome pathway | BRANCHED-CHAIN AMINO ACID CATABOLISM | 20 | -2.49 | <0.0001 | *<0.0001* | 2.34 | <0.0001 | *0.0003* |
| M2 Reactome pathway | MITOCHONDRIAL PROTEIN DEGRADATION | 78 | -2.79 | <0.0001 | *<0.0001* | 2.33 | <0.0001 | *0.0002* |
| M2 Reactome pathway | PYRUVATE METABOLISM | 42 | -2.35 | <0.0001 | *<0.0001* | 2.28 | <0.0001 | *0.0002* |
| M2 Reactome pathway | SYNTHESIS OF ACTIVE UBIQUITIN ROLES OF E1 AND E2 ENZYMES | 30 | -1.69 | 0.0158 | *0.0680* | 2.28 | <0.0001 | *0.0002* |
| M5 GO Biological Process | BRANCHED-CHAIN AMINO ACID CATABOLIC PROCESS | 21 | -2.47 | <0.0001 | *<0.0001* | 2.25 | <0.0001 | *0.0003* |
| M5 GO Cellular Component | MITOCHONDRIAL PROTEIN CONTAINING COMPLEX | 259 | -2.57 | <0.0001 | *<0.0001* | 2.24 | <0.0001 | *0.0005* |
| M2 WikiPathways pathway | TCA CYCLE | 30 | -2.50 | <0.0001 | *<0.0001* | 2.21 | <0.0001 | *0.0006* |
| M5 GO Biological Process | BRANCHED-CHAIN AMINO ACID METABOLIC PROCESS | 25 | -2.47 | <0.0001 | *<0.0001* | 2.20 | <0.0001 | *0.0008* |
| M5 GO Cellular Component | ALPHA-KETOACID DEHYDROGENASE COMPLEX | 15 | -2.32 | <0.0001 | *<0.0001* | 2.16 | <0.0001 | *0.0030* |
| M5 GO Biological Process | MITOCHONDRIAL FISSION | 49 | -1.91 | <0.0001 | *0.0128* | 2.15 | <0.0001 | *0.0031* |
| M5 GO Cellular Component | PHOSPHATASE COMPLEX | 49 | -1.21 | 0.1870 | *0.4281* | 2.14 | <0.0001 | *0.0033* |
| M5 GO Molecular Function | NUCLEAR LOCALIZATION SEQUENCE BINDING | 23 | -1.50 | 0.0252 | *0.1724* | 2.13 | <0.0001 | *0.0034* |
| M2 Reactome pathway | CITRIC ACID CYCLE (TCA CYCLE) | 33 | -2.45 | <0.0001 | *<0.0001* | 2.13 | <0.0001 | *0.0034* |
| M2 Reactome pathway | REGULATION OF PYRUVATE METABOLISM | 32 | -2.30 | <0.0001 | *<0.0001* | 2.12 | <0.0001 | *0.0042* |
| M2 Reactome pathway | PROTEIN UBIQUITINATION | 71 | -1.30 | 0.0771 | *0.3273* | 2.09 | <0.0001 | *0.0053* |
| M5 GO Cellular Component | MITOCHONDRIAL MATRIX | 289 | -2.78 | 0.0000 | *0.0000* | 2.09 | <0.0001 | *0.0057* |
| M2 BioCarta pathway | IGF1-MTOR PATHWAY | 18 | -1.34 | 0.1141 | *0.2963* | 2.06 | <0.0001 | *0.0082* |
| M5 GO Biological Process | POSITIVE REGULATION OF INTERFERON-ALPHA PRODUCTION | 28 | -1.87 | <0.0001 | *0.0179* | 2.05 | <0.0001 | *0.0092* |

**Table S5. Oppositely regulated proteins (DOWN in R403Q, UP by CDD866) from the Venn diagram in Figure S13A.** Of total 119 overlapping proteins between the effects of R403Q mutation in placebo-treated animals and CDD866 in R403Q hearts (n = 5-6 animals per group), 62 proteins were regulated in opposite directions. Of those 62 oppositely regulated proteins, 47 were downregulated by R403Q mutation and upregulated by CDD866. Proteins with significant differential abundance across comparisons were identified using two-sided independent-samples Student's t-tests for each protein. mean FC, mean fold change; p-value, nominal *p*-value; BH p-val, Benjamini-Hochberg-corrected p-value. $p<0.05$ was considered significant. Proteins coordinately upregulated are depicted in red; Proteins coordinately downregulated are depicted in blue.

| Oppositely regulated proteins: DOWN in R403Q and UP by CDD866 | | | R403Q-placebo vs WT-placebo | | | R403Q-CDD866 vs R403Q-placebo | | |
|---|---|---|---|---|---|---|---|---|
| **Protein full name** | **Protein symbol** | **UniProt ID** | **Mean FC** | **p-value** | **BH p-val** | **Mean FC** | **p-value** | **BH p-val** |
| FXYD domain-containing ion transport regulator 4 | FXYD4 | P59646 | 0.5922 | ***0.0122*** | *0.9978* | 2.4127 | **0.0005** | *0.1527* |
| Isovaleryl-CoA dehydrogenase, mitochondrial | IVD | P26440 | 0.7029 | ***0.0347*** | *0.9978* | 1.4687 | **0.0392** | *0.4201* |
| Protein Wnt-16 | WNT16 | Q9UBV4 | 0.7160 | ***0.0078*** | *0.9978* | 2.3927 | **0.0432** | *0.4368* |
| N-terminal EF-hand calcium-binding protein 3 | NECA3 | Q96P71 | 0.7343 | ***0.0318*** | *0.9978* | 1.5078 | **0.0178** | *0.3388* |
| cAMP-dependent protein kinase type I-beta regulatory subunit | KAP1 | P31321 | 0.7600 | ***0.0177*** | *0.9978* | 1.3347 | **0.0166** | *0.3324* |
| Dipeptidyl peptidase 8 | DPP8 | Q6V1X1 | 0.7639 | ***0.0104*** | *0.9978* | 1.5782 | **0.0046** | *0.2611* |
| Ras-related C3 botulinum toxin substrate 1 | RAC1 | P63000 | 0.7988 | ***0.0161*** | *0.9978* | 1.7349 | **0.0039** | *0.2440* |
| [Pyruvate dehydrogenase (acetyl-transferring)] kinase isozyme 1, mitochondrial | PDK1 | Q15118 | 0.7993 | ***0.0036*** | *0.9978* | 1.2701 | ***<0.0001*** | *0.1462* |
| cAMP-dependent protein kinase type I-alpha regulatory subunit | KAP0 | P10644 | 0.7997 | ***0.0137*** | *0.9978* | 1.3277 | **0.0081** | *0.2880* |
| GMP reductase 1 | GMPR1 | P36959 | 0.8067 | ***0.0248*** | *0.9978* | 1.2217 | **0.0247** | *0.3778* |
| NF-kappa-B inhibitor-interacting Ras-like protein 1 | KBRS1 | Q9NYS0 | 0.8098 | ***0.0024*** | *0.9978* | 1.2378 | **0.0058** | *0.2759* |
| Diphosphoinositol polyphosphate phosphohydrolase 3-beta | NUD11 | Q96G61 | 0.8262 | ***0.0055*** | *0.9978* | 1.1146 | **0.0176** | *0.3388* |
| Polyadenylate-binding protein 1 | PABP1 | P11940 | 0.8296 | ***0.0082*** | *0.9978* | 1.1343 | **0.0413** | *0.4311* |
| Polymerase delta-interacting protein 2 | PDIP2 | Q9Y2S7 | 0.8372 | ***0.0448*** | *0.9978* | 1.2434 | **0.0120** | *0.3167* |
| N-myc-interactor | NMI | Q13287 | 0.8386 | ***0.0385*** | *0.9978* | 1.2525 | **0.0051** | *0.2684* |
| Steroid receptor RNA activator 1 | SRA1 | Q9HD15 | 0.8404 | ***0.0053*** | *0.9978* | 1.1631 | **0.0303** | *0.3965* |
| Protein Wnt-7a | WNT7A | O00755 | 0.8426 | ***0.0209*** | *0.9978* | 1.6209 | **0.0369** | *0.4155* |
| Neural Wiskott-Aldrich syndrome protein | WASL | O00401 | 0.8488 | ***0.0020*** | *0.9978* | 1.2032 | **0.0010** | *0.1832* |
| NEDD8-conjugating enzyme Ubc12 | UBC12 | P61081 | 0.8520 | ***0.0026*** | *0.9978* | 1.3398 | **0.0001** | *0.1462* |
| Activating transcription factor 7-interacting protein 2 | MCAF2 | Q5U623 | 0.8551 | ***0.0426*** | *0.9978* | 1.2584 | **0.0059** | *0.2759* |
| Nuclear receptor coactivator 1 | NCOA1 | Q15788 | 0.8641 | ***0.0023*** | *0.9978* | 1.2405 | **0.0157** | *0.3290* |
| Type I inositol 1,4,5-trisphosphate 5-phosphatase | I5P1 | Q14642 | 0.8688 | ***0.0052*** | *0.9978* | 1.3993 | **0.0387** | *0.4194* |
| tRNA (cytosine(38)-C(5))-methyltransferase | TRDMT | O14717 | 0.8713 | ***0.0293*** | *0.9978* | 1.2905 | ***<0.0001*** | *0.1462* |
| Arf-GAP domain and FG repeat-containing protein 2 | AGFG2 | O95081 | 0.8810 | ***0.0391*** | *0.9978* | 1.2931 | **0.0266** | *0.3802* |
| Eukaryotic translation initiation factor 5A-1 | eIF-5A-1 | P63241 | 0.8873 | ***0.0356*** | *0.9978* | 1.1993 | **0.0208** | *0.3595* |
| Sorting nexin-27 | SNX27 | Q96L92 | 0.8915 | ***0.0332*** | *0.9978* | 1.1980 | **0.0031** | *0.2339* |
| 40S ribosomal protein S5 | RS5 | P46782 | 0.8932 | ***0.0167*** | *0.9978* | 1.1292 | **0.0055** | *0.2753* |
| 26S proteasome non-ATPase regulatory subunit 4 | PSMD4 | P55036 | 0.8941 | ***0.0484*** | *0.9978* | 1.0875 | **0.0261** | *0.3778* |
| Prefoldin subunit 3 | PFD3 | P61758 | 0.8943 | ***0.0005*** | *0.9978* | 1.1257 | **0.0233** | *0.3730* |
| Bridging integrator 3 | BIN3 | Q9NQY0 | 0.8949 | ***0.0492*** | *0.9978* | 1.1578 | **0.0257** | *0.3778* |
| SOSS complex subunit B1 | SOSB1 | Q9BQ15 | 0.8973 | ***0.0170*** | *0.9978* | 1.1670 | **0.0178** | *0.3388* |
| Cancer/testis antigen family 45 member A6 | CT456 | P0DMU7 | 0.9017 | ***0.0181*** | *0.9978* | 1.0857 | **0.0234** | *0.3730* |
| Protein SCO2 homolog, mitochondrial | SCO2 | O43819 | 0.9074 | ***0.0429*** | *0.9978* | 1.1411 | **0.0363** | *0.4154* |
| Cytokine receptor-like factor 1 | CRLF1 | O75462 | 0.9076 | ***0.0036*** | *0.9978* | 1.0801 | **0.0090** | *0.2929* |
| Ras-related protein Rab-3D | RAB3D | O95716 | 0.9101 | ***0.0181*** | *0.9978* | 1.1362 | **0.0004** | *0.1466* |
| HIRA-interacting protein 3 | HIRP3 | Q9BW71 | 0.9179 | ***0.0235*** | *0.9978* | 1.0490 | **0.0436** | *0.4368* |
| SOSS complex subunit B2 | SOSB2 | Q96AH0 | 0.9210 | ***0.0473*** | *0.9978* | 1.1902 | **0.0063** | *0.2773* |
| Protein max | MAX | P61244 | 0.9338 | ***0.0022*** | *0.9978* | 1.0525 | **0.0355** | *0.4127* |
| Prostasin | Prostasin | Q16651 | 0.9390 | ***0.0159*** | *0.9978* | 1.0444 | **0.0344** | *0.4110* |
| Probable RNA-binding protein EIF1AD | EIF1A | Q8N9N8 | 0.9409 | ***0.0076*** | *0.9978* | 1.0643 | **0.0170** | *0.3336* |
| Eukaryotic translation initiation factor 4E-binding protein 1 | 4EBP1 | Q13541 | 0.9414 | ***0.0114*** | *0.9978* | 1.1171 | **0.0092** | *0.2942* |
| Phospholipase A-2-activating protein | PLAA | Q9Y263 | 0.9432 | ***0.0425*** | *0.9978* | 1.0821 | **0.0003** | *0.1463* |
| Sulfite oxidase, mitochondrial | SUOX | P51687 | 0.9435 | ***0.0174*** | *0.9978* | 1.0853 | **0.0138** | *0.3211* |
| Ubiquitin-conjugating enzyme E2 Q1 | UB2Q1 | Q7Z7E8 | 0.9457 | ***0.0331*** | *0.9978* | 1.2088 | **0.0087** | *0.2920* |
| Interferon beta | IFN-b | P01574 | 0.9525 | ***0.0238*** | *0.9978* | 1.0475 | **0.0405** | *0.4269* |
| Coiled-coil domain-containing protein 97 | CCD97 | Q96F63 | 0.9563 | ***0.0353*** | *0.9978* | 1.1405 | **0.0004** | *0.1472* |
| DNA excision repair protein ERCC-8 | ERCC8 | Q13216 | 0.9602 | ***0.0116*** | *0.9978* | 1.0727 | **0.0010** | *0.1814* |

**Table S6. Oppositely regulated proteins (UP in R403Q, DOWN by CDD866) from the Venn diagram in Figure S13A.** Of total 119 overlapping proteins between the effects of R403Q mutation in placebo-treated animals and CDD866 in R403Q hearts (n = 5-6 animals per group), 62 proteins were regulated in opposite directions. Of those 62 oppositely regulated proteins, 15 were upregulated by R403Q mutation and downregulated by CDD866. Proteins with significant differential abundance across comparisons were identified using two-sided independent-samples Student's t-tests for each protein. mean FC, mean fold change; p-value, nominal *p*-value; BH p-val, Benjamini-Hochberg-corrected p-value. p<0.05 was considered significant. Proteins coordinately upregulated are depicted in red; Proteins coordinately downregulated are depicted in blue.

| Oppositely regulated proteins: UP in R403Q and DOWN by CDD866 | | | R403Q-placebo vs WT-placebo | | | R403Q-CDD866 vs R403Q-placebo | | |
|---|---|---|---|---|---|---|---|---|
| **Protein full name** | **Protein symbol** | **UniProt ID** | **Mean FC** | **p-value** | **BH p-val** | **Mean FC** | **p-value** | **BH p-val** |
| Cofilin-2 | COF2 | Q9Y281 | 1.8373 | ***0.0165*** | *0.9978* | 0.5944 | ***0.0214*** | *0.3618* |
| UBX domain-containing protein 10 | UBX10 | Q96LJ8 | 1.2786 | ***0.0050*** | *0.9978* | 0.8446 | ***0.0442*** | *0.4384* |
| Cdc42 effector protein 3 | BORG2 | Q9UKI2 | 1.1579 | ***0.0252*** | *0.9978* | 0.8638 | ***0.0090*** | *0.2932* |
| Citrate synthase, mitochondrial | CISY | O75390 | 1.1454 | ***0.0219*** | *0.9978* | 0.8707 | ***0.0326*** | *0.4030* |
| WAP four-disulfide core domain protein 6 | WFDC6 | Q9BQY6 | 1.0882 | ***0.0213*** | *0.9978* | 0.9407 | ***0.0485*** | *0.4509* |
| Bcl-2-modifying factor | BMF | Q96LC9 | 1.0761 | ***0.0178*** | *0.9978* | 0.9241 | ***0.0442*** | *0.4384* |
| ATP-dependent RNA helicase DDX3Y | DDX3Y | O15523 | 1.0725 | ***0.0255*** | *0.9978* | 0.9428 | ***0.0289*** | *0.3920* |
| Isoform L-VEGF165 | L-VEGF165 | P15692 | 1.0627 | ***0.0138*** | *0.9978* | 0.9593 | ***0.0441*** | *0.4384* |
| TLC domain-containing protein 1 | TLCD1 | Q96CP7 | 1.0626 | ***0.0367*** | *0.9978* | 0.9493 | ***0.0465*** | *0.4451* |
| Protein FAM10A4 | F10A4 | Q8IZP2 | 1.0590 | ***0.0058*** | *0.9978* | 0.9494 | ***0.0122*** | *0.3191* |
| Low-density lipoprotein receptor-related protein 1, soluble:Cytoplasmic domain | sLRP1:CD | Q07954 | 1.0574 | ***0.0053*** | *0.9978* | 0.9696 | ***0.0342*** | *0.4110* |
| 7-methylguanosine phosphate-specific 5'-nucleotidase | Cytosolic 5'-nucleotidase III-like protein | Q969T7 | 1.0495 | ***0.0450*** | *0.9978* | 0.9495 | ***0.0257*** | *0.3778* |
| Cadherin-12:Cytoplasmic domain | Cadherin-12:CD | P55289 | 1.0390 | ***0.0307*** | *0.9978* | 0.9401 | ***0.0115*** | *0.3137* |
| NmrA-like family domain-containing protein 1 | NMRL1 | Q9HBL8 | 1.0351 | ***0.0487*** | *0.9978* | 0.9609 | ***0.0100*** | *0.3038* |
| Zinc finger protein 582 | ZN582 | Q96NG8 | 1.0343 | ***0.0336*** | *0.9978* | 0.9501 | ***0.0076*** | *0.2880* |

**Table S7. Top 20 proteins up- or down-regulated by R403Q in placebo-treated animals.** Hearts of 28-week-old animals after 4 weeks of placebo or CDD866 treatment were used **(**n = 5-6 animals per group). Proteins with significant differential abundance across comparisons were identified using two-sided independent-samples Student's t-tests for each protein. mean FC, mean fold change; p-value, nominal *p*-value; BH p-val, Benjamini-Hochberg-corrected p-value. p<0.05 was considered significant. Proteins coordinately upregulated are depicted in red; Proteins coordinately downregulated are depicted in blue.

| 20 most DOWN-regulated proteins (by mean FC) in R403Q-placebo vs WT-placebo | | | R403Q-placebo vs WT-placebo | | |
|---|---|---|---|---|---|
| **Protein full name** | **Protein symbol** | **UniProt ID** | **Mean FC** | **p-value** | **BH p-val** |
| Tektin-3 | TEKT3 | Q9BXF9 | 0.5708 | ***0.0267*** | *0.9978* |
| FXYD domain-containing ion transport regulator 4 | FXYD4 | P59646 | 0.5922 | ***0.0122*** | *0.9978* |
| HMG-1:Box A oxidized | HMG-1:Box A oxidized | P09429 | 0.6920 | ***0.0181*** | *0.9978* |
| Isovaleryl-CoA dehydrogenase, mitochondrial | IVD | P26440 | 0.7029 | ***0.0347*** | *0.9978* |
| Fatty aldehyde dehydrogenase | AL3A2 | P51648 | 0.7090 | ***0.0039*** | *0.9978* |
| Protein Wnt-16 | WNT16 | Q9UBV4 | 0.7160 | ***0.0078*** | *0.9978* |
| MARVEL domain-containing protein 2 | MALD2 | Q8N4S9 | 0.7224 | ***0.0001*** | *0.6302* |
| Succinyl-CoA ligase | SUCB1 | Q9P2R7 | 0.7286 | ***0.0244*** | *0.9978* |
| N-terminal EF-hand calcium-binding protein 3 | NECA3 | Q96P71 | 0.7343 | ***0.0318*** | *0.9978* |
| Translation initiation factor IF-3, mitochondrial | IF3M | Q9H2K0 | 0.7356 | ***0.0245*** | *0.9978* |
| Transcription factor A, mitochondrial | TFAM | Q00059 | 0.7575 | ***0.0015*** | *0.9978* |
| cAMP-dependent protein kinase type I-beta regulatory subunit | KAP1 | P31321 | 0.7600 | ***0.0177*** | *0.9978* |
| Dipeptidyl peptidase 8 | DPP8 | Q6V1X1 | 0.7639 | ***0.0104*** | *0.9978* |
| tRNA (adenine(58)-N(1))-methyltransferase non-catalytic subunit TRM6 | TRM6 | Q9UJA5 | 0.7693 | ***0.0040*** | *0.9978* |
| Thioredoxin-like protein AAED1 | AAED1 | Q7RTV5 | 0.7706 | ***0.0093*** | *0.9978* |
| EF-hand calcium-binding domain-containing protein 9 | EFCB9 | A8MZ26 | 0.7787 | ***0.0070*** | *0.9978* |
| 2-oxoisovalerate dehydrogenase subunit alpha, mitochondrial | ODBA | P12694 | 0.7825 | ***0.0037*** | *0.9978* |
| Twisted gastrulation protein homolog 1 | TWSG1 | Q9GZX9 | 0.7826 | ***0.0023*** | *0.9978* |
| E3 ubiquitin-protein ligase RNF31 | RNF31 | Q96EP0 | 0.7833 | ***0.0001*** | *0.6302* |
| Ras-related C3 botulinum toxin substrate 1 | RAC1 | P63000 | 0.7988 | ***0.0161*** | *0.9978* |

| 20 most UP-regulated proteins (by mean FC) in R403Q-placebo vs WT-placebo | | | R403Q-placebo vs WT-placebo | | |
|---|---|---|---|---|---|
| **Protein full name** | **Protein symbol** | **UniProt ID** | **Mean FC** | **p-value** | **BH p-val** |
| Ras-related protein Ral-B | RALB | P11234 | 1.9439 | ***0.0364*** | *0.9978* |
| Cofilin-2 | COF2 | Q9Y281 | 1.8373 | ***0.0165*** | *0.9978* |
| SHC SH2 domain-binding protein 1 | SHCBP | Q8NEM2 | 1.7508 | ***0.0378*** | *0.9978* |
| Beta-casein | Beta-casein | P05814 | 1.7236 | ***0.0239*** | *0.9978* |
| Protein RTF2 homolog | RTF2 | Q9BY42 | 1.6844 | ***0.0294*** | *0.9978* |
| Insulin-like growth factor II:Mature | IGF-II:Mature | P01344 | 1.6675 | ***0.0169*** | *0.9978* |
| Protein FAM19A1 | F19A1 | Q7Z5A9 | 1.6568 | ***0.0260*** | *0.9978* |
| Baculoviral IAP repeat-containing protein 2 | cIAP-1 | Q13490 | 1.6536 | ***0.0408*** | *0.9978* |
| Cytoskeleton-associated protein 4 | CKAP4 | Q07065 | 1.6497 | ***0.0330*** | *0.9978* |
| Forkhead box protein G1 | FOXGB | P55316 | 1.5974 | ***0.0383*** | *0.9978* |
| Dynactin subunit 6 | DCTN6 | O00399 | 1.5616 | ***0.0498*** | *0.9978* |
| Protein-arginine deiminase type-1 | PADI1 | Q9ULC6 | 1.5457 | ***0.0117*** | *0.9978* |
| Sarcolemmal membrane-associated protein | SLMAP | Q14BN4 | 1.5295 | ***0.0284*** | *0.9978* |
| Reticulon-4 receptor-like 2 | R4RL2 | Q86UN3 | 1.4605 | ***0.0361*** | *0.9978* |
| Creatine kinase U-type, mitochondrial | KCRU | P12532 | 1.4469 | ***0.0131*** | *0.9978* |
| MHC class I polypeptide-related sequence B | MICB | Q29980 | 1.4425 | ***0.0355*** | *0.9978* |
| NAD(P)H dehydrogenase [quinone] 1 | NAD(P)H dehydrogenase | P15559 | 1.4234 | ***0.0255*** | *0.9978* |
| Ras-related protein Rab-18 | RAB18 | Q9NP72 | 1.4135 | ***0.0350*** | *0.9978* |
| MORN repeat-containing protein 4 | MORN4 | Q8NDC4 | 1.4126 | ***0.0435*** | *0.9978* |
| Butyrophilin subfamily 3 member A1 | BT3A1 | O00481 | 1.3971 | ***0.0286*** | *0.9978* |

**Table S8. Top 20 proteins up- or down-regulated by CDD866 in R403Q hearts.** Hearts of 28-week-old animals after 4 weeks of placebo or CDD866 treatment were used **(**n = 5-6 animals per groups). Proteins with significant differential abundance across comparisons were identified using two-sided independent-samples Student's t-tests for each protein. mean FC, mean fold change; p-value, nominal *p*-value; BH p-val, Benjamini-Hochberg-corrected p-value. p<0.05 was considered significant. Proteins coordinately upregulated are depicted in red; Proteins coordinately downregulated are depicted in blue.

| 20 most DOWN-regulated proteins (by mean FC) in R403Q-CDD866 vs R403Q-placebo | | | R403Q-CDD866 vs R403Q-placebo | | |
|---|---|---|---|---|---|
| **Protein full name** | **Protein symbol** | **UniProt ID** | **Mean FC** | **p-value** | **BH p-val** |
| Heterogeneous nuclear ribonucleoprotein D-like | HNRDL | O14979 | 0.5670 | ***0.0340*** | *0.4106* |
| Cofilin-2 | COF2 | Q9Y281 | 0.5944 | ***0.0214*** | *0.3618* |
| Dynein heavy chain 11, axonemal | DYH11 | Q96DT5 | 0.6178 | ***0.0144*** | *0.3211* |
| Fibrinogen gamma chain | Fibrinogen g-chain dimer | P02679 | 0.6449 | ***0.0447*** | *0.4390* |
| Interleukin-18 | IL-18 | Q14116 | 0.6900 | ***0.0186*** | *0.3415* |
| Succinate dehydrogenase [ubiquinone] iron-sulfur subunit, mitochondrial | SDHB | P21912 | 0.7177 | ***0.0089*** | *0.2925* |
| Ubiquitin-related modifier 1 | URM1 | Q9BTM9 | 0.7218 | ***0.0040*** | *0.2440* |
| Peroxisomal multifunctional enzyme type 2 | DHB4 | P51659 | 0.7258 | ***0.0198*** | *0.3549* |
| Insulin-like growth factor-binding protein 2 | IGFBP-2 | P18065 | 0.7477 | ***0.0320*** | *0.4030* |
| Catalase | Catalase | P04040 | 0.7686 | ***0.0415*** | *0.4315* |
| Cardiac Troponin I-T-C Complex | Troponin I-T-C | P45379\|P63316\|P19429 | 0.7707 | ***0.0068*** | *0.2793* |
| Beta-enolase | ENOB | P13929 | 0.7736 | ***0.0183*** | *0.3402* |
| Isochorismatase domain-containing protein 1 | ISOC1 | Q96CN7 | 0.7777 | ***0.0145*** | *0.3211* |
| Proline-rich transmembrane protein 4 | PRRT4 | C9JH25 | 0.7852 | ***0.0133*** | *0.3211* |
| Nucleoporin p54 | NUP54 | Q7Z3B4 | 0.7911 | ***0.0295*** | *0.3937* |
| Calcium-binding tyrosine phosphorylation-regulated protein | CABYR | O75952 | 0.7967 | ***0.0211*** | *0.3595* |
| DNL-type zinc finger protein | DNLZ | Q5SXM8 | 0.8002 | ***0.0154*** | *0.3264* |
| Ras-related protein Rap-1A | RAP1A | P62834 | 0.8004 | ***0.0359*** | *0.4144* |
| MOB kinase activator 2 | MOB2 | Q70IA6 | 0.8007 | ***0.0250*** | *0.3778* |
| Troponin I, fast skeletal muscle | Troponin I, skeletal, fast twitch | P48788 | 0.8026 | ***0.0425*** | *0.4362* |

| 20 most UP-regulated proteins (by mean FC) in R403Q-CDD866 vs R403Q-placebo | | | R403Q-CDD866 vs R403Q-placebo | | |
|---|---|---|---|---|---|
| **Protein full name** | **Protein symbol** | **UniProt ID** | **Mean FC** | **p-value** | **BH p-val** |
| Glycerol-3-phosphate dehydrogenase [NAD(+)], cytoplasmic | GPDA | P21695 | 4.3397 | ***0.0108*** | *0.3080* |
| L-lactate dehydrogenase B chain | LDH-H 1 | P07195 | 3.2344 | ***0.0165*** | *0.3324* |
| L-lactate dehydrogenase A chain | LDHA | P00338 | 2.7858 | ***0.0055*** | *0.2753* |
| Reticulocalbin-1 | RCN1 | Q15293 | 2.4967 | ***0.0310*** | *0.3971* |
| FXYD domain-containing ion transport regulator 4 | FXYD4 | P59646 | 2.4127 | ***0.0005*** | *0.1527* |
| Cytosolic 5'-nucleotidase 1A | 5NT1A | Q9BXI3 | 2.4065 | ***0.0055*** | *0.2753* |
| Protein Wnt-16 | WNT16 | Q9UBV4 | 2.3927 | ***0.0432*** | *0.4368* |
| CASP8 and FADD-like apoptosis regulator | FLIP | O15519 | 2.2954 | ***0.0240*** | *0.3759* |
| Glyceraldehyde-3-phosphate dehydrogenase | GAPDH, liver | P04406 | 1.9446 | ***0.0036*** | *0.2440* |
| Oxidized low-density lipoprotein receptor 1 | OLR1 | P78380 | 1.8636 | ***0.0013*** | *0.1950* |
| Muscleblind-like protein 1 | MBNL1 | Q9NR56 | 1.8425 | ***0.0036*** | *0.2440* |
| ADP-ribosylation factor-like protein 14 | ARL14 | Q8N4G2 | 1.8349 | ***0.0012*** | *0.1928* |
| eIF-2-alpha kinase GCN2 | E2AK4 | Q9P2K8 | 1.8342 | ***0.0012*** | *0.1928* |
| Mediator of RNA polymerase II transcription subunit 21 | MED21 | Q13503 | 1.8271 | ***0.0023*** | *0.2259* |
| Troponin I, slow skeletal muscle | TNNI1 | P19237 | 1.8251 | ***0.0036*** | *0.2440* |
| Fibroblast growth factor 8 isoform B | FGF-8B | P55075 | 1.8166 | ***0.0044*** | *0.2523* |
| Glyceraldehyde-3-phosphate dehydrogenase | GAPDH, liver | P04406 | 1.8127 | ***0.0081*** | *0.2880* |
| Deoxyhypusine synthase | DHYS | P49366 | 1.8051 | ***0.0031*** | *0.2347* |
| Nuclear receptor 0B2 | SHP | Q15466 | 1.7957 | ***0.0024*** | *0.2263* |
| Muscleblind-like protein 2 | MBNL2 | Q5VZF2 | 1.7900 | ***0.0027*** | *0.2265* |

**Table S9. Integrated cardiac RNA and protein upstream regulator analysis.** Regulator names in bold identify entries included in Figure 4. IPA activation z-scores for all 88 regulator entries meeting nominal overlap $p < 0.05$ in both disease and treatment comparisons in each omics layer. No z-score magnitude or directional-concordance threshold was applied for inclusion. Disease denotes R403Q-placebo versus WT-placebo; CDD866 denotes R403Q-CDD866 versus R403Q-placebo. RNA denotes cardiac RNA-seq; protein denotes cardiac SomaScan proteomics. Overlap p-values were not adjusted for multiple testing. Rows proceed down the left block and then the right, ordered by directional pattern, with concordant disease-to-treatment sign reversals first. Red and blue indicate positive and negative z-scores, respectively. Bold scores have $|z| \geq 2$; smaller absolute scores represent weaker directional predictions. NA indicates that the regulator was listed but no activation z-score was reported. A dagger (†) identifies an IPA directional-bias flag in the RNA treatment export. Entries include genes, families, complexes and chemical/perturbation signatures; chemical entries do not imply that the compound was administered or measured. Opposite disease and treatment signs indicate potential counter-regulation, not complete normalization or causal mediation. No individual SomaScan proteins survived Benjamini-Hochberg FDR correction; the proteomic component and integrated regulator comparisons are exploratory.

**Supplementary Table 9. Shared IPA upstream-regulator predictions**

Cardiac RNA-seq and SomaScan proteomics (88 regulators)

| Regulator | Disease | | CDD866 | |
|---|---|---|---|---|
| | RNA | Protein | RNA | Protein |
| **HNF4A** | +0.07 | **+2.16** | -0.94 | -0.79 |
| CHUK | +0.86 | +0.73 | -0.97† | -0.11 |
| trans-hydroxytamoxifen | +1.21 | +1.89 | -0.38 | -1.34 |
| dactolisib | **+2.45** | +1.34 | **-2.00†** | -1.94 |
| diethylstilbestrol | **+2.24** | +1.80 | -1.25† | -1.26 |
| **MYC** | -1.06 | -0.56 | **+2.40†** | **+2.71** |
| **PPARG** | **-2.09** | -0.60 | +1.69 | +0.51 |
| beta-estradiol | -1.73 | -0.56 | **+2.86** | +1.52 |
| Z-LLL-CHO | -1.93 | -1.19 | +1.07† | **+2.02** |
| **insulin (family)** | -0.67 | -0.13 | +0.26 | +1.16 |
| **EGFR** | -1.02 | -0.11 | +1.36† | +1.31 |
| MEK | -0.16 | -1.13 | +1.07 | +0.52 |
| IL4 | -0.32 | -0.05 | **+2.03†** | +0.32 |
| dimethyl n-oxalyl-glycine | -1.68 | -0.49 | +0.90† | +1.63 |
| CD3 | -0.43 | -0.06 | **+3.15†** | +0.40 |
| **EP300** | -1.43 | **-2.19** | +1.34† | +0.60 |
| AGT | +0.80 | +0.43 | +0.44† | +1.86 |
| lipopolysaccharide | **+2.07** | +0.66 | **+3.71†** | **+2.37** |
| CG | +1.49 | +1.29 | +1.95† | +0.04 |
| IGF2BP1 | +1.34 | +1.85 | +0.05 | +1.74 |
| AR | +0.73 | +0.41 | +0.61 | +1.25 |
| HGF | +1.40 | +1.10 | +0.17† | **+2.14** |
| HRAS | +0.26 | +0.39 | +0.71 | +0.20 |
| IL2 | +0.63 | **+2.30** | +1.06† | +1.04 |
| ARID1A | **+2.25** | +0.30 | +1.41 | +1.13 |
| SMARCA4 | +0.90 | +0.96 | +1.98† | +0.25 |
| IKBKB | -0.44 | -1.58 | -1.51† | -0.11 |
| estrogen receptor | -0.34 | -1.33 | **-2.33** | **-2.24** |
| metformin | -1.75 | -1.79 | -0.75 | -1.52 |
| mir-8 (includes others) | -1.57 | -1.10 | -1.34† | -1.06 |
| mir-802 | -1.27 | 0.00 | **-2.83†** | -1.41 |
| LH | +1.51 | -0.07 | +0.24† | **+2.12** |
| FSH | +0.06 | -0.12 | +0.90 | +0.44 |
| doxorubicin | +0.05 | -0.25 | +0.53 | +0.70 |
| TGFB1 | **+2.44** | -0.76 | +1.45 | +1.13 |
| poly rI:rC-RNA | +0.08 | -0.47 | +1.28† | +0.76 |
| CD44 | **+2.90** | -0.85 | +1.34† | +1.34 |
| EPAS1 | +0.03 | -0.55 | +0.98† | +1.31 |
| IL1B | +0.31 | -0.29 | +0.31 | +0.41 |
| CREBBP | +0.01 | -0.39 | **+2.20†** | +0.28 |
| dexamethasone | -1.02 | +0.54 | +1.50 | +1.33 |
| **PPARGC1A** | **-5.35** | +0.69 | +0.60 | +1.32 |
| rosiglitazone | **-5.12** | +1.75 | +1.52 | +1.54 |
| SP1 | -1.45 | +1.13 | +0.28† | +1.26 |

| Regulator (continued) | Disease | | CDD866 | |
|---|---|---|---|---|
| | RNA | Protein | RNA | Protein |
| **NFE2L2** | -0.65 | +1.35 | **+2.14** | +0.59 |
| tetradecanoylphorbol acetate | -0.03 | +0.94 | +0.85† | +0.65 |
| NR3C1 | **-2.01** | +1.28 | +0.64 | +0.35 |
| ERBB3 | -0.91 | +0.29 | +0.82 | +0.19 |
| RELA | -1.41 | +0.47 | +1.58† | +0.82 |
| ERG | -1.29 | +1.63 | +0.52† | +1.00 |
| IFNG | -0.23 | +0.17 | +1.11 | **+2.13** |
| PTEN | -1.29 | +0.42 | -0.14 | **-2.44** |
| calcitriol | **-2.26** | +0.02 | -1.76 | -0.10 |
| thymoquinone | -1.24 | +1.91 | -1.00 | -1.00 |
| KRAS | +0.81 | +0.09 | +1.30 | -0.31 |
| FGF2 | +0.21 | +0.67 | +1.22† | -1.27 |
| p38 MAPK | +1.23 | +0.07 | +1.83† | -0.32 |
| FGFR2 | +0.34 | +0.13 | **+2.21** | -0.58 |
| CTNNB1 | **+3.32** | +0.07 | +1.03† | -0.12 |
| SIRT1 | +0.07 | +0.26 | -0.01 | +0.66 |
| methylprednisolone | -0.85 | -1.06 | +0.57 | -0.52 |
| ERBB2 | -0.26 | **-2.06** | +0.48 | -1.42 |
| hydrogen peroxide | -0.36 | -1.39 | +0.96† | -0.03 |
| mir-17 (includes others) | -0.17 | -0.11 | **-2.17†** | +0.60 |
| MYCN | -1.69 | -1.44 | -1.36 | +1.09 |
| IGF1R | +0.15 | -0.29 | +0.08† | -0.64 |
| TK1 | +0.09 | -0.56 | +0.24 | -0.10 |
| cisplatin | +0.73 | -0.06 | +1.91 | **-2.04** |
| TP53 | **+2.47** | -1.08 | -1.01 | +0.66 |
| bisindolylmaleimide I | +1.51 | -1.43 | -0.71† | **+2.21** |
| ESR1 | -1.17 | +0.92 | **+2.50** | -0.13 |
| palmitic acid | -0.21 | +0.30 | +1.90 | -0.87 |
| APP | -0.37 | +0.98 | +0.32 | -0.69 |
| FOS | -0.93 | +0.30 | +0.57 | -0.65 |
| nitric oxide | -0.34 | +0.51 | +1.73 | -0.89 |
| JUN | -1.20 | +0.68 | -0.01† | +0.34 |
| Immunoglobulin | -0.81 | +0.78 | -0.25 | +0.85 |
| ESRRG | -0.79 | *NA* | -0.13† | **+2.19** |
| NFKBIA | +1.06 | +0.44 | -0.54 | 0.00 |
| raloxifene | +1.26 | +0.38 | -0.82 | 0.00 |
| HDAC4 | +0.39 | *NA* | *NA* | +1.06 |
| CDH11 | +1.66 | *NA* | *NA* | +0.90 |
| HTT | +1.50 | *NA* | *NA* | -0.93 |
| EIF2AK4 | -0.17 | *NA* | -0.76 | *NA* |
| SLC1A5 | -0.76 | *NA* | *NA* | *NA* |
| RYR1 | *NA* | *NA* | *NA* | *NA* |
| Pick1 | *NA* | *NA* | *NA* | *NA* |
| HOMER1 | *NA* | *NA* | *NA* | *NA* |

**Supplementary Videos**

**Video S1. A representative 3D micro-CT rendering of lower hindlimb muscle volume in WT-placebo.**

**Video S2. A representative 3D micro-CT rendering of lower hindlimb muscle volume in R403Q-placebo.**

**Video S3. A representative 3D micro-CT rendering of lower hindlimb muscle volume in R403Q-CDD866.**

## References


[1] J.Z. Lin, N. Rabhi, S.R. Farmer. Myocardin-Related Transcription Factor A Promotes Recruitment of ITGA5+ Profibrotic Progenitors during Obesity-Induced Adipose Tissue Fibrosis. Cell Rep. 23 (2018) 1977–1987.

[2] R.M. Weisbrod, T. Shiang, L. Al Sayah, J.L. Fry, S. Bajpai, C.A. Reinhart-King, et al. Arterial stiffening precedes systolic hypertension in diet-induced obesity. Hypertension 62 (2013) 1105–1110.

[3] B. van der Heyden, W.R.P.H. van de Worp, A. van Helvoort, J. Theys, A.M.W.J. Schols, R.C.J. Langen, et al. Automated CT-derived skeletal muscle mass determination in lower hind limbs of mice using a 3D U-Net deep learning network. J. Appl. Physiol. 128 (2020) 42–49.

[4] L. Pasetto, D. Olivari, G. Nardo, M. Chiara Trolese, C. Bendotti, R. Piccirillo, et al. Micro-computed tomography for non-invasive evaluation of muscle atrophy in mouse models of disease. PLoS One 13 (2018) e0198089.

[5] A. Bonetto, D.C. Andersson, D.L. Waning. Assessment of muscle mass and strength in mice. Bonekey Rep. 4 (2015) 732.

[6] J.J. Munier, J.T. Pank, A. Severino, H. Wang, P. Zhang, L. Vergnes, et al. Simultaneous monitoring of mouse grip strength, force profile, and cumulative force profile distinguishes muscle physiology following surgical, pharmacologic and diet interventions. Sci. Rep. 12 (2022) 16428.

[7] R.M.J. Deacon. Measuring the strength of mice. J. Vis. Exp. (2013) 2610.

[8] A. Aartsma-Rus, M. van Putten. Assessing functional performance in the mdx mouse model. J. Vis. Exp. (2014) 51303.

[9] S.M. Klein, J. Vykoukal, P. Lechler, K. Zeitler, S. Gehmert, S. Schreml, et al. Noninvasive in vivo assessment of muscle impairment in the mdx mouse model--a

comparison of two common wire hanging methods with two different results. J. Neurosci. Methods 203 (2012) 292–297.

[10] A. Biolo, R. Greferath, D.A. Siwik, F. Qin, E. Valsky, K.C. Fylaktakidou, et al. Enhanced exercise capacity in mice with severe heart failure treated with an allosteric effector of hemoglobin, myo-inositol trispyrophosphate. Proc. Natl. Acad. Sci. U. S. A. 106 (2009) 1926–1929.

[11] F. Qin, S. Lennon-Edwards, S. Lancel, A. Biolo, D.A. Siwik, D.R. Pimentel, et al. Cardiac-specific overexpression of catalase identifies hydrogen peroxide-dependent and-independent phases of myocardial remodeling and prevents the progression to overt heart failure in Gαq-overexpressing transgenic mice. Circ. Hear. Fail. 3 (2010) 306–313.

[12] D. Croteau, I. Luptak, J.M. Chambers, I. Hobai, M. Panagia, D.R. Pimentel, et al. Effects of Sodium-Glucose Linked Transporter 2 Inhibition With Ertugliflozin on Mitochondrial Function, Energetics, and Metabolic Gene Expression in the Presence and Absence of Diabetes Mellitus in Mice. J. Am. Heart Assoc. 10 (2021) e019995.

[13] T. Baka, J. Moore, F. Qin, S.R. Yurista, A. Zhang, H. He, et al. Empagliflozin enhances metabolic efficiency and improves left ventricular hypertrophy in a hypertrophic cardiomyopathy mouse model. Eur. Heart J. 46 (2025) 4105–4119.

[14] P.F. Pedro, A. Tsakmaki, G.A. Bewick. The Glucose Tolerance Test in Mice. Methods Mol. Biol. 2128 (2020) 207–216.

[15] C. Moro, C. Magnan. Revisited guidelines for metabolic tolerance tests in mice. Lab Anim. 2024 541 54 (2024) 16–23.

[16] F. Qin, D.A. Siwik, I. Luptak, X. Hou, L. Wang, A. Higuchi, et al. The polyphenols resveratrol and S17834 prevent the structural and functional sequelae of diet-induced metabolic heart disease in mice. Circulation 125 (2012) 1757–1764.

[17] I. Luptak, M. Shen, H. He, M.F. Hirshman, N. Musi, L.J. Goodyear, et al. Aberrant activation of AMP-activated protein kinase remodels metabolic network in favor of cardiac glycogen storage. J. Clin. Investig. 117 (2007) 1432–1439.

[18] I. Luptak, J. Yan, L. Cui, M. Jain, R. Liao, R. Tian. Long-term effects of increased glucose entry on mouse hearts during normal aging and ischemic stress. Circulation 116 (2007) 901–909.

[19] I. Luptak, J.A. Balschi, Y. Xing, T.C. Leone, D.P. Kelly, R. Tian. Decreased contractile and metabolic reserve in peroxisome proliferator-activated receptor-α-null hearts can be rescued by increasing glucose transport and utilization. Circulation 112 (2005) 2339–2346.

[20] C.R. Malloy, A.D. Sherry, H. Jeffrey. Evaluation of Carbon Flux and Substrate Selection through Alternate Pathways Involving the Citric Acid Cycle of the Heart by 13C NMR Spectroscopy. J. Biol. Chem. 263 (1988) 6964–6971.

[21] C. Stringer, T. Wang, M. Michaelos, M. Pachitariu. Cellpose: a generalist algorithm for cellular segmentation. Nat. Methods 18 (2021) 100–106.

[22] J. Candia. SomaScan Bioinformatics: Normalization, Quality Control, and Assessment of Pre-Analytical Variation. Methods Mol. Biol. 2929 (2025) 107–127.

[23] J. Candia, G. Fantoni, F. Delgado-Peraza, N. Shehadeh, T. Tanaka, R. Moaddel, et al. Variability of 7K and 11K SomaScan Plasma Proteomics Assays. J. Proteome Res. 23 (2024) 5531–5539.

[24] P. Virtanen, R. Gommers, T.E. Oliphant, M. Haberland, T. Reddy, D. Cournapeau, et al. SciPy 1.0: fundamental algorithms for scientific computing in Python. Nat. Methods 17 (2020) 261–272.

[25] Y. Benjaminit, Y. Hochberg. Controlling the False Discovery Rate: A Practical and Powerful Approach to Multiple Testing. J. R. Stat. Soc. Ser. B Stat. Methodol. 57

(1995) 289–300.

[26] H. Heberle, V.G. Meirelles, F.R. da Silva, G.P. Telles, R. Minghim. InteractiVenn: a web-based tool for the analysis of sets through Venn diagrams. BMC Bioinformatics 16 (2015) 169.
